# Experimental Design Signatures Emerge from Topic Modeling of Graphene Oxide Literature

**DOI:** 10.1021/acsnano.6c04548

**Published:** 2026-10-17

**Authors:** Henry J. Kobs, Henrique Ferreira, Liu Yang, Amauri J. Paula, Carlos E. Andrade, Andreia F. Faria

**Affiliations:** † Engineering School of Sustainable Infrastructure & Environment, Department of Environmental Engineering Sciences, 3463 University of Florida, Gainesville, 32611-6580, Florida, United States; ‡ Solid-Biological Interface Group (SolBIN), 74362 Aeronautics Institute of Technology − ITA, São José dos Campos, 12228-900, SP, Brazil; § AT&T Laboratories Research, 1405 US Hwy 206, Bedminster, 07921, New Jersey, United States

**Keywords:** graphene oxide, topic modeling, information extraction, large language models, machine learning, CO_2_ adsorption

## Abstract

The literature on graphene oxide (GO) comprises hundreds of thousands of publications spanning synthesis, characterization, and applications in diverse domains. Advancing GO materials requires a structured knowledge map derived from systematic mining of this extensive corpus. However, extracting meaningful insights from this vast, heterogeneous, sparse, and unstructured data remains a challenge. To address this challenge, Natural Language Processing techniques, including topic modeling, community discovery, and parameter extraction, are here employed to establish a framework. Latent Dirichlet Allocation (LDA) was applied to the full text of more than 120,000 articles, enabling the identification of eight macro-domains within the GO literature, and neural topic modeling was subsequently employed to characterize the granularity and heterogeneity of these domains. Parameter extraction was performed using Large Language Models (LLMs) on a subset of 8000 full-text articles, with a proto-ontology incorporated into the extraction prompt. Even with a broad prompt, an F1 score of 94% was achieved against manual validation. The extracted parameters were then clustered through community modeling and compared with the LDA-derived topics. At this intermediate resolution of the literature, we demonstrate the application of a recommendation system for relevant parameters according to the material family and application domain. The applicability of the framework at higher resolutions was further demonstrated through the construction of a data set of CO_2_ adsorption for graphene- and GO-based materials. This data set was subsequently employed in a downstream predictive case study using an Extremely Randomized Trees Regressor, with different training-test splitting strategies evaluated to assess in-domain and out-of-domain generalization at article-level resolution.

## Graphical abstract

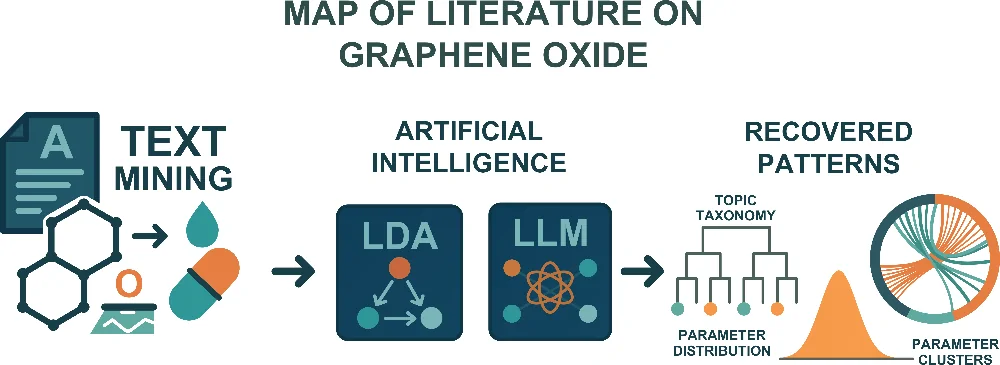

Graphene oxide (GO) offers a cost-effective and scalable approach to producing materials that exhibit graphene-like properties.^1–12^ GO is synthesized by oxidizing graphite and exfoliating it into thin graphene sheets that are functionalized with oxygen-containing groups. This oxidation process allows for the processing of GO in water while preserving many of the relevant properties of graphene.^1–4^ The diversity of these oxygen-containing functionalities confers GO sheets with tunable surface chemistry, enhanced interfacial adhesion, and aqueous dispersibility, supporting applications in water purification, membrane technology, catalysis, sensing, polymer reinforcement, and flexible electronics.^5–12^ Throughout this work, the GO literature is treated operationally as a GO-based material family, including graphite oxide, GO, reduced GO (rGO), functionalized GO, GO composites, GO-derived films, membranes, fibers, and related hybrid materials when they appear in the source corpus.

The widespread utilization of GO is influenced by the carbon-to-oxygen ratio, the distribution of oxygen-containing functional groups (e.g., hydroxyl and carboxyl), defect density, flake size, and hydrophilicity.^3,4,13^ Due to the interchangeable nature of these features and diverse reporting in the GO literature, isolating structure–property correlations for properties such as permeability, conductivity, mechanical strength, and adsorption capacity remains challenging.^14,15^ As a result, after decades of rapid growth, the GO literature is large and heterogeneous,^4,14^ preventing the inference of robust structure–property correlations. Variations in synthesis methods, characterization protocols, lack of well-defined stoichiometry, and nonstandardized terminologies impede rigorous cross-domain comparisons,^15–17^ thus complicating the identification of consistent trends or knowledge gaps.

Addressing these challenges requires a systematic map of the GO literature that operates at three nested levels: macrotaxonomy (what are the major research domains?), within-domain organization (how does each domain subdivide into coherent subtopics?), and experimental design structure (which parameters are coreported, revealing characteristic protocols?). Conventional bibliometric approaches fail at all three levels because they rely on titles and abstracts, which lack the technical details necessary to distinguish closely related application areas or to extract experimental parameters.

To address these challenges, we use Natural Language Processing (NLP), a field in computer science and artificial intelligence that provides the text-mining tools required to build a literature-scale map from unstructured scientific documents.^18–20^ Several studies have already applied NLP-based approaches to analogous problems in materials science, including the extraction of inorganic synthesis recipes and synthesis procedures,^21–23^ magnetocaloric materials data and property records,^24^ semiconductor and photovoltaic materials data,^25,26^ CO_2_ reduction electrocatalyst and synthesis information,^27–29^ and nanomaterial toxicity patterns and property extraction.^30–32^ Beyond literature mining, Large Language Models (LLMs) can also be used more directly in inverse design and constrained generation, including prompt-guided molecular design, drug-like molecule generation, and materials optimization under user-specified objectives or constraints.^33–35^ Together, these developments have opened a practical path for extracting structure from large scientific corpora and for using that structure to support downstream discovery. These topic-defined domains then provide the context for experimental analysis, where parameters extracted from full text are used to construct co-occurrence networks whose community structure reveals domain-specific experimental signatures.

We apply NLP methods capable of analyzing the full-text scientific literature on a large scale. Our workflow consists of three stages (Figure 1). We first constructed a full-text corpus by indexing, downloading, and preprocessing more than 120,000 GO publications. This corpus enables modeling of topic structure at two complementary resolutions: latent Dirichlet allocation (LDA) recovers a stable macrotaxonomy of the field, while neural topic modeling resolves subtopic landscapes within each macrodomain. This macrotaxonomic map, recovered with LDA and refined within each macrodomain using neural topic modeling,^36^ supports hierarchical analysis and shows that application areas exhibit systematically different internal fragmentation patterns. Further, a parameter layer then changes the role of text mining from literature classification to experimental structure discovery: instead of asking only which articles belong to which topic, it asks which reported variables co-occur as experimental designs and which contextual descriptors must be retained for property-specific data construction. Together, these results provide a structured framework for navigating the GO literature, prioritizing variables from experimental protocols, and identifying opportunities for cross-domain knowledge transfer that are inaccessible to conventional bibliometric analysis.

**Figure 1:**
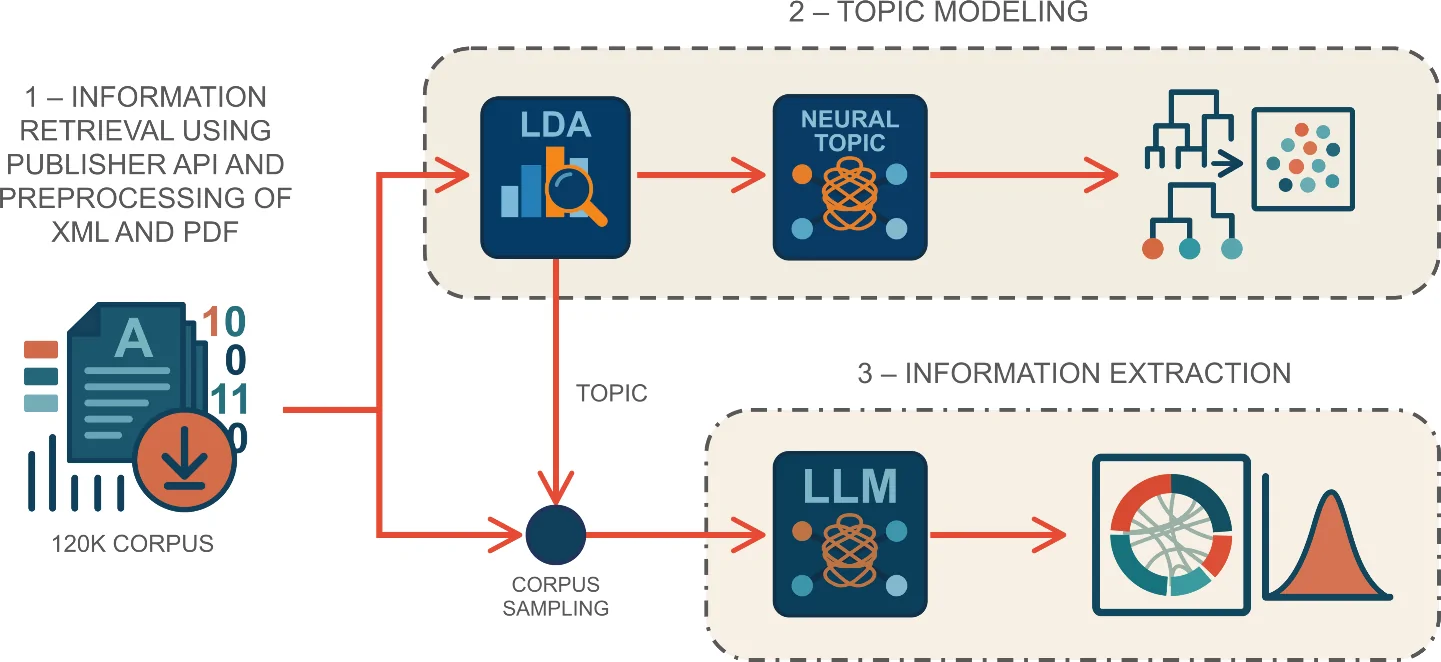
Three main stages workflow. First, we assemble the corpus by indexing, downloading, and preprocessing full-text GO publications. Second, we apply topic modeling at two scales: Latent Dirichlet allocation (LDA) for macro-taxonomy and neural topic modeling for subtopic landscapes within each macro-domain. Third, normalized full-text parameters from topic-level extraction sets are converted into an all-topic communities graph.

## Results and Discussion

### Topics Modeling

#### Topic Macrostructure

Here, the body of literature on GO comprises more than 120,000 publications. However, their overarching structure remains poorly understood. To perform a comprehensive analysis of experimental practices or relationships between structures and properties, it is essential to establish a reliable classification system. Traditional bibliometric methods for developing these classifications typically rely on examination of titles and abstracts. Unfortunately, these sources often lack the technical details needed to accurately distinguish among related application domains and do not provide sufficient information to effectively extract the experimental parameters. This situation raises an important question: Can a detailed analysis of full-text publications yield a more coherent, distinct macrotaxonomy?

Because the corpus was assembled from both XML (Extensible Markup Language)^37^ and PDF (Portable Document Format)^38^ files, as exemplified in Figure 1, full-text extraction required two complementary processing routes before all documents could enter a unified preprocessing pipeline. XML articles provided the most direct route because their author-structured content could be parsed and transferred directly to subsequent text-cleaning and normalization steps. PDF articles required more careful handling, because their internal representation varied substantially between publishers and files. Native PDFs, which contained machine-readable text, allowed the direct extraction of text from the document layer, preserving the content of the article for subsequent processing. However, some PDFs contained very little embedded text because visible page content was stored primarily as scanned or publisher-generated images rather than as selectable characters. Therefore, these image-based documents required an optical character recognition (OCR) stage to recover their textual content, which we performed primarily with GOT-OCR 2.0.^39^ By combining direct parsing of XML files, direct extraction of native PDFs, and optical character recognition (OCR) of image-based PDFs, this workflow converted heterogeneous source documents to a consistent full-text representation suitable for large-scale downstream analysis.

Having extracted the full text for a corpus of 120,000 articles, we applied Latent Dirichlet Allocation (LDA) to both the full text and title abstract corpora and treated the number of topics *K* as a tunable hyperparameter. We trained models over a range of *K* values (1–50) and compared topic quality using *C*_*v*_ coherence^40^ and topic separability. Full-text models consistently outperformed title–abstract models in all *K* evaluated, with a roughly constant difference. The maximum for full-text occurred when *K* = 8, producing the best-performing and most interpretable macro-taxonomy (coherence *C*_*v*_ = 0.727 for full text vs *C*_*v*_ = 0.647 for title–abstract; Figure 2a). This difference reflects the richer and semantically unique vocabulary present in the full articles. At *K* = 8 the model resolved eight application-centered domains: (1) Catalysis, (2) Energy Storage, (3) Polymer & Composite Reinforcement, (4) Soft Matter & Scaffolds, (5) Biomedical & Drug Delivery, (6) Water Treatment & Membrane, (7) Nanosensors, and (8) Films & Coatings. Details of this analysis can be found in the Supporting Information (SI), Section B.

**Figure 2:**
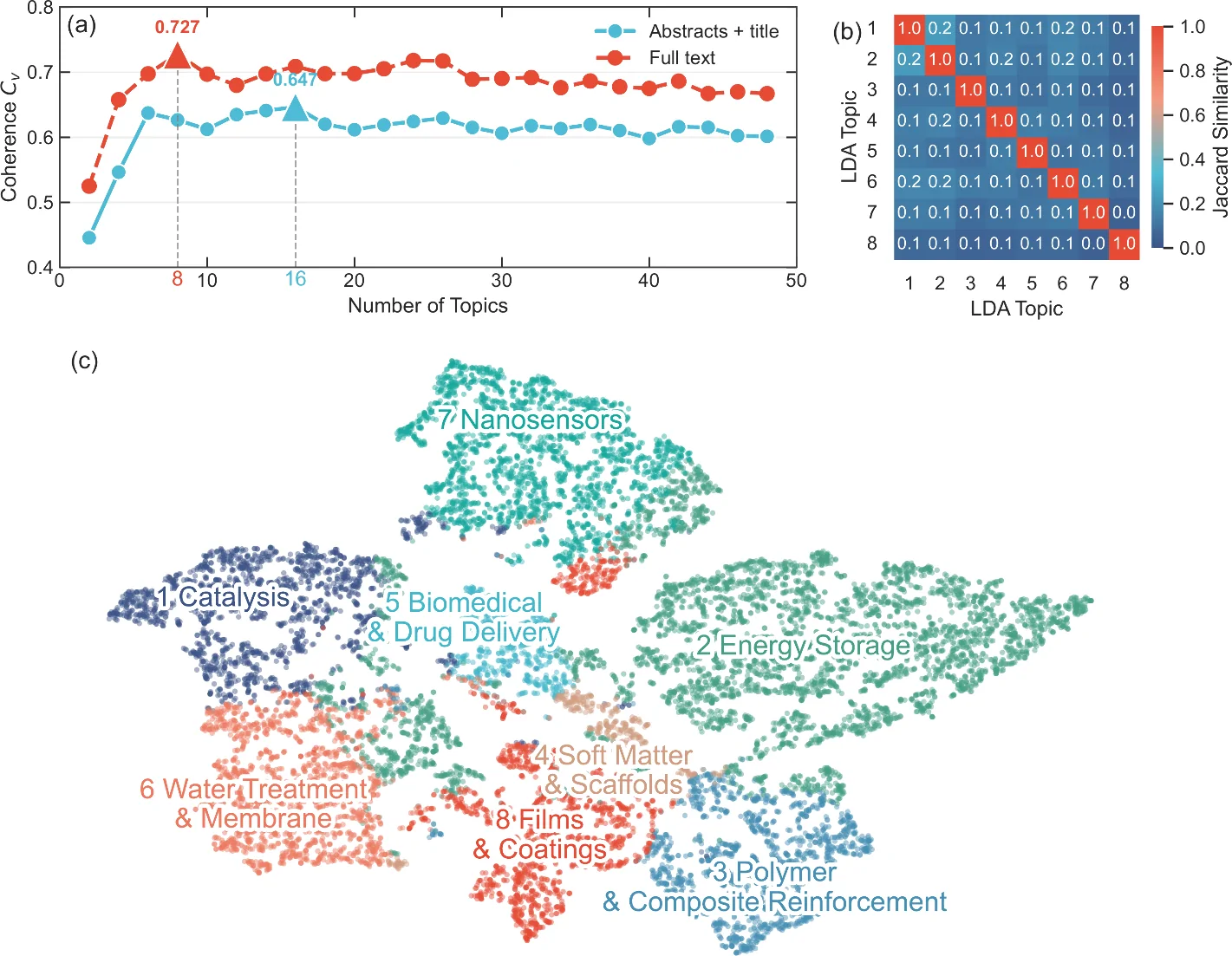
Topics macrostructure. (a) Topic coherence *C*_*v*_ as a function of the number of LDA topics *K* for models trained on full texts (red) versus title + abstract only (blue), highlighting the improved interpretability of full-text models and the selected solution (*K* = 8). (b) Keyword-level Jaccard similarity matrix computed between the eight topic vocabularies, showing minimal cross-topic leakage. (c) Two-dimensional t-SNE projection of document TF–IDF vectors for the full-text corpus, colored by the predominant LDA topic; labeled regions correspond to the eight major application domains identified in the GO literature.

The selected eight-topic solution exhibits limited vocabulary overlap, as quantified by Jaccard similarity at the keyword level: off-diagonal similarities remain at or below 0.23, with the largest overlap observed between Catalysis and Energy Storage (Figure 2b). This separability supports the interpretation of the topics as lexically distinct scientific domains. To construct a graphical representation, we applied t-Distributed Stochastic Neighbor Embedding (t-SNE)^41^ of the Term Frequency-Inverse Document Frequency (TF–IDF)^42^ document vector of each article. The t-SNE projection of the TF–IDF document vectors, colored by the predominant LDA topic, reveals distinct semantic regions corresponding to the eight major application domains (Figure 2c). The spatial arrangement reflects lexical similarity, with proximate topics sharing vocabulary and research foundations, while the overall separation validates the macro-taxonomic structure.

#### Subtopic Heterogeneity

Although the macrotaxonomy describes the principal application areas, it tends to consolidate a variety of distinct research communities under a single class. This consolidation is pertinent because researchers navigate their domains through the intricate networks of their specific subfields rather than relying solely on broad domain labels. In addition, the different domains exhibit diverse internal structures. Therefore, a single narrative cannot comprehensively account for the unique attributes of all of the areas. In some domains, distinctions are made among a few large groups based on function, while in others, a multitude of smaller clusters exists, categorized by technique or target. To effectively capture these complexities, a representation that articulates the subtopics within each macrodomain is imperative.

A neural topic model alone can recover fine-grained structures, but it does not reliably provide the macrolevel structure needed for domain comparison. At the corpus scale, neural clusters can be pulled together by shared methodological language that cuts across applications, mixing distinct domains into the same cluster, and blurring the boundaries one later wants to compare. That same sensitivity makes the global partition less stable and less interpretable as a domain index, thereby weakening any claim about how fragmentation differs across domains.

To address this challenge, we performed a two-stage topic modeling strategy that intentionally separates the global organization from the details intrinsic to each domain. In this paradigm, LDA is utilized to create a coherent macrotaxonomy that classifies the corpus into broad application areas, which will subsequently serve as a foundational framework for cross-domain comparisons. Although this macrolevel partition proves beneficial, it inevitably compresses fine-grained variations, as the remaining differences within a domain are often driven by mechanisms, device classes, assays, and protocol families that do not clearly separate based on word co-occurrence alone. Neural topic modeling complements this limitation by recovering subtopic neighborhoods within each macrodomain, enabling internal structure to be compared without confounding from cross-domain method vocabulary.

To characterize within-domain structure, we trained a neural topic modeling pipeline separately within each LDA macrotopic^36,43^ using Qwen-3-Embedding representations^44^ and projected the resulting clusters into Uniform Manifold Approximation and Projection (UMAP) space.^45^ In these projections, gray points denote the full set of documents assigned to a given macrodomain, whereas the colored islands mark representative subtopic regions summarized by automatically inferred labels (Figure 3). This approach renders the internal geography of each domain directly visible, making subtopics interpretable as coherent semantic neighborhoods. It further reveals that fragmentation is not uniform across the literature: the spatial structure of the UMAP maps changes systematically across application areas, indicating that each domain is organized along its own dominant semantic axis.

**Figure 3:**
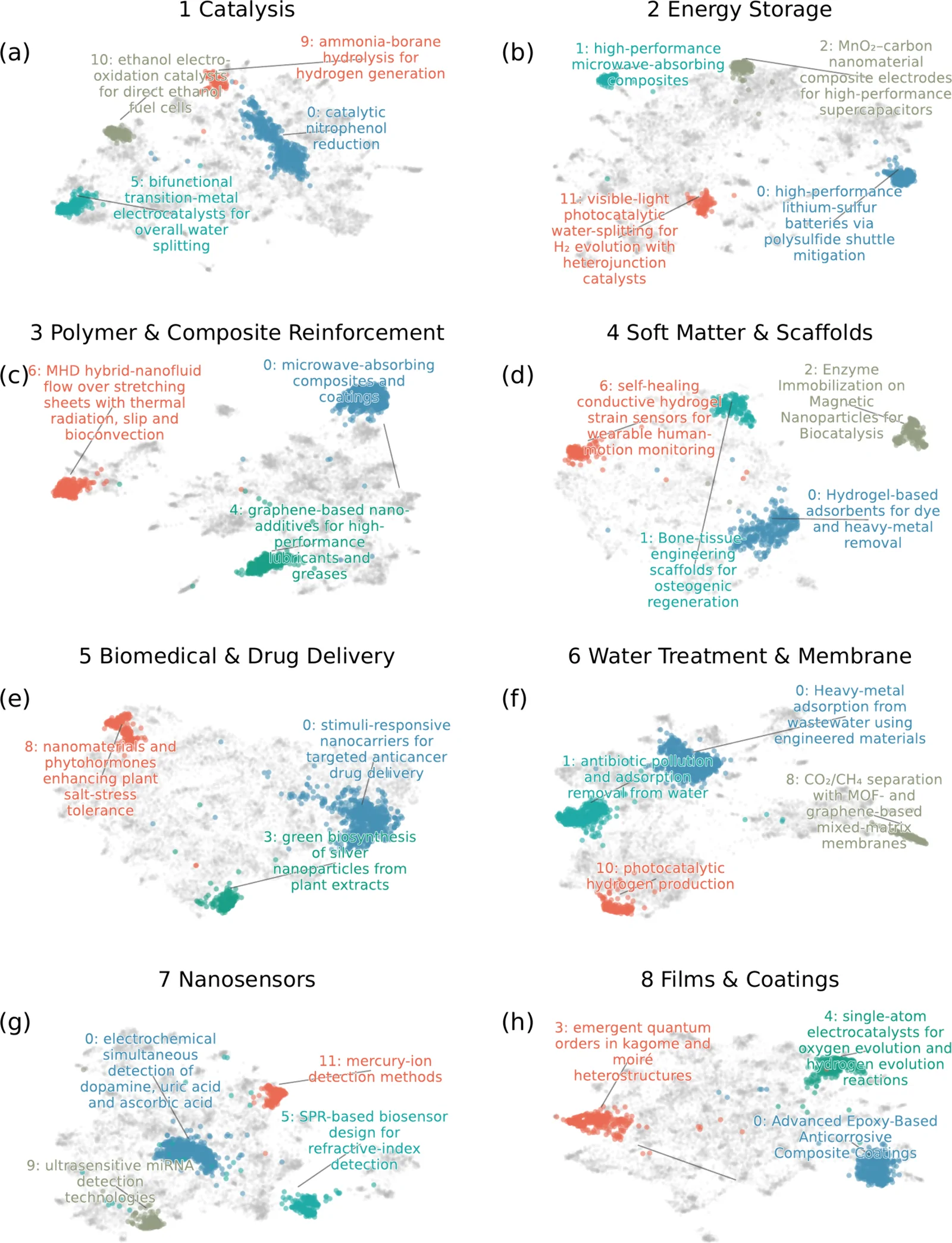
Subtopic landscape. (a–h) UMAP projections of Neural Topic Modeling document embeddings computed separately within each LDA macro-topic. Each point represents a document; gray points indicate the full topic-specific document set, and highlighted clusters mark a representative Neural Topic Modeling subtopic. Labels denote the automatically inferred thematic summaries of the highlighted subtopics.

Catalysis and Energy Storage illustrate this point clearly. In Catalysis, the map resolves a set of relatively compact islands centered on reaction-specific electrochemical and catalytic functions, including general water splitting, reduction of nitrophenols, electro-oxidation of ethanol, and hydrolysis of ammonia–borane for hydrogen generation (Figure 3a). The resulting geometry suggests that this domain is primarily structured by the reaction class and the catalytic mechanism rather than by a single generic catalysis narrative. Energy Storage shows a related but broader organization. Its dominant islands are anchored by supercapacitor electrodes and lithium–sulfur battery studies, but the same map also contains neighboring regions associated with microwave-absorbing composites and photocatalytic water-splitting materials (Figure 3b). This broader spread indicates that the domain is held together by a shared electrochemical and functional-materials vocabulary, even when the end applications differ. In other words, the internal landscape of Energy Storage is less narrowly defined by a single device class than by a broader language of charge transport, interfaces, and performance engineering.

Water Treatment & Membrane is organized differently because its subtopic regions are separated by treatment mechanism. One major neighborhood focuses on wastewater heavy-metal adsorption, another on antibiotic adsorption and removal, and another on photocatalytic routes, while a more isolated branch captures mixed metal–organic framework and graphene-based matrix membranes for CO_2_/CH_4_ separation (Figure 3f). The key pattern is that this domain does not form a single, continuous remediation cluster. Instead, it divides into adsorption-centered, reaction-driven, and separation-centered neighborhoods, each with its own local vocabulary. The UMAP structure, therefore, shows that water-related GO research is organized more strongly by the treatment strategy than by pollutant identity alone.

A different type of fragmentation appears in Nanosensors and Biomedical & Drug Delivery. In Nanosensors, the map breaks into several clearly separated islands corresponding to simultaneous electrochemical detection, surface plasmon resonance (SPR)-based biosensor design, mercury-ion detection, and ultrasensitive microRNA detection (Figure 3g). This pattern indicates a highly fractured domain in which platform technology and the analyte class act as the main organizing variables. The category label “sensor” is very broad to describe the actual semantic structure of the field because the local neighborhoods are defined by distinct transduction modes and targets. Biomedical & Drug Delivery is also heterogeneous, but the source of its heterogeneity is different. Its highlighted regions include stimuli-responsive nanocarriers for targeted anticancer delivery, green biosynthesis of silver nanoparticles from plant extracts, and nanomaterial–phytohormone strategies to enhance salt-stress tolerance in plants (Figure 3e). Rather than converging on a single biomedical core, this map spreads across therapeutic delivery, biologically derived nanomaterial production, and bioenabled agricultural applications. Therefore, the internal landscape is structured by the experimental workflow and the biological use context, not by a single unified nanomedicine theme.

Polymer & Composite Reinforcement, Soft Matter & Scaffolds, and Films & Coatings show yet another family of organizing principles. In Polymer & Composite Reinforcement, the highlighted islands correspond to microwave-absorbing composites and coatings, graphene-based nanoadditives for high-performance lubricants and greases, and hybrid nanofluid flow systems with thermal-radiation and slip effects (Figure 3c). These regions are less linked by a common host matrix than by distinct performance targets such as electromagnetic response, tribology, and thermal transport. Soft Matter & Scaffolds, by contrast, is unified by form factor and matrix chemistry. Its map contains neighborhoods for bone-tissue engineering scaffolds, hydrogel-based adsorbents for dye and heavy-metal removal, enzyme immobilization on magnetic nanoparticles for biocatalysis, and self-healing conductive hydrogels for wearable monitoring (Figure 3d). Here, hydrated, porous, and biofunctional material formats provide a shared semantic framework, even when the application changes from regeneration to remediation or wearable devices. Films & Coatings is the most bifurcated of the three. One side of its map is anchored by advanced epoxy-based anticorrosive composite coatings, while other regions correspond to single-atom electrocatalysts for oxygen and hydrogen evolution and to emergent quantum orders in kagome and heterostructures moiré (Figure 3h). This split indicates that the domain contains two distinct narratives: one focused on surface protection and multifunctional coatings, and another focused on thin-film or interface-enabled device physics.

Taken together, the UMAP projections show that each macrodomain contains multiple coherent semantic neighborhoods, each structured differently depending on the specific domain. These observed patterns motivate a hierarchical representation of the GO literature, moving from broad application domains to finer subtopic neighborhoods within each domain. This Perspective reveals not only the breadth of research directions across the field but also the distinct structural dynamics that govern individual domain communities.

In Figure 4, for each LDA macrotopic, the upper panel shows a dendrogram built from neural subtopic representations, with the dashed line marking the threshold used to define higher-level clusters. The lower panel reports the document count for each subtopic, colored by its assigned higher level branch, and the numbers above the bars indicate the total document count captured by each branch. Together, these panels distinguish domains dominated by only a few large conceptual families from those distributed across several substantial branches. Thus, Figure 4 provides a compact readout of hierarchical heterogeneity in the GO literature, while Section B.5 of the SI clarifies which scientific dimensions drive the observed partitions.

**Figure 4:**
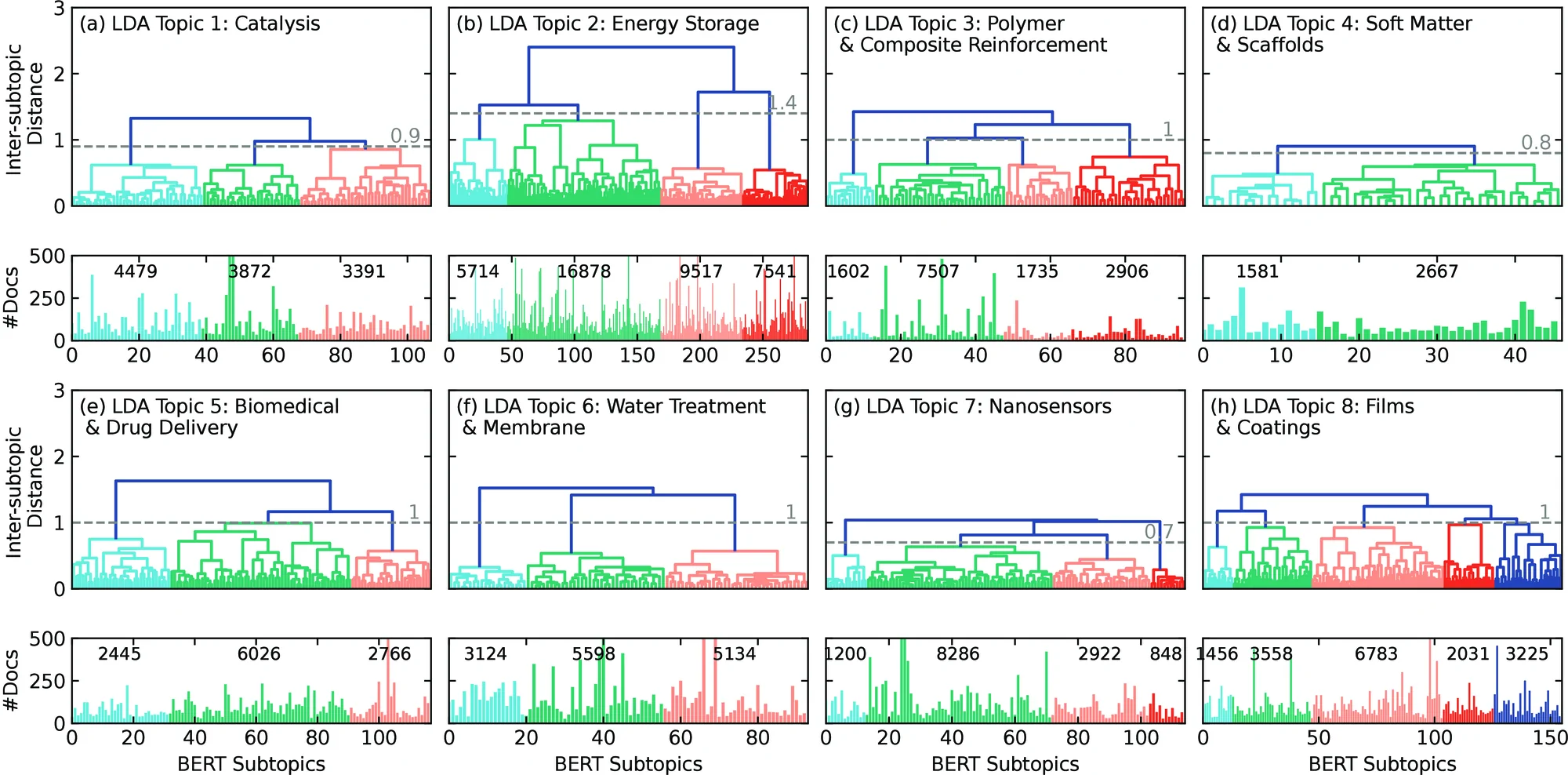
Hierarchical organization and heterogeneity of subtopics across GO application domains. For each LDA macro-topic (a–h), the upper panel shows a hierarchical dendrogram constructed from Neural Topic Modeling subtopic representations (intersubtopic distance), and the dashed line indicates the cut threshold used to define higher-level subtopic clusters. The lower panel shows the document count per subtopic, colored by the assigned higher-level cluster; numbers above the bars indicate the total document count aggregated within each cluster.

Some domains are notable for their conformational changes. Soft Matter & Scaffolds (Figure 4d) is the clearest example, resolving into only two higher-level branches containing 1581 and 2667 documents. Water Treatment & Membrane (Figure 4f) also remains compact, divided into three dominant branches containing 3124, 5598, and 5134 documents. Catalysis (Figure 4a) shows a similar structure, with three branches of broadly comparable size (4479, 3872, and 3391 documents). In each case, most of the subtopics collapse into a small number of large clusters, indicating that the literature is organized around a limited set of dominant research characteristics. As detailed in section B.5 of the SI, this consolidation reflects strong recurring organizing principles: matrix form and scaffold function in Soft Matter & Scaffolds, treatment mechanism in Water Treatment & Membrane, and reaction class and electrochemical context in Catalysis. Because these axes dominate the internal structure of each domain, many fine-grained subtopics remain close enough to merge into only a few major higher level families.

At the other extreme, several domains remain more broadly distributed. Films & Coatings (Figure 4h) resolves into five branches containing 456, 3558, 6783, 2031, and 3225 documents, giving the broadest partitioning among the eight macrotopics. Energy Storage (Figure 4b) also shows pronounced branching, with four large groups containing 5714, 16878, 9517, and 7541 documents, consistent with a macro-topic spanning multiple major electrochemical subareas. Nanosensors (Figure 4g) also separates into four branches, although one branch dominates strongly (8286 documents) relative to the others (2922, 1200, and 848 documents), indicating a large central core accompanied by narrower offshoots. As shown in section B.5 of the SI, these broader partitions arise because several technical configurations coexist within each domain. Films and Coatings separates the device-physics directions from surface functionality and interface-oriented work. Energy storage spans supercapacitors, batteries, and related electrochemical systems, and nanosensors divides simultaneously by analyte class and transduction modality. These domains, therefore, remain internally broader, because they are shaped by several partially independent research trajectories rather than by a single dominant organizing principle.

Polymer & Composite Reinforcement and Biomedical & Drug Delivery occupy an intermediate regime. Polymer & Composite Reinforcement (Figure 4c) divides into four branches, but two are comparatively small (1602 and 1735 documents) relative to the larger branches of 7507 and 2906 documents, indicating clear specialization without an even distribution across all groups. Biomedical & Drug Delivery (Figure 4e) is divided into three branches that contain 2445, 6026, and 2766 documents, again showing substantial internal diversity while remaining less dispersed than Films & Coatings or Energy Storage. As detailed in section B.5 of the SI, these intermediate patterns reflect domains organized by more than one strong axis but not by as many competing axes as in the most distributed cases. In Polymer & Composite Reinforcement, the main separations follow the host material family and the target performance function, whereas in Biomedical and Drug Delivery, they follow the experimental workflow, including delivery, therapy, antimicrobial platforms, and detection.

In general, the hierarchical patterns in Figure 4 show that GO research diversifies through distinct domain-specific pathways rather than through a single common structure. In some application areas, the literature converges into a few dominant branches, consistent with strong organizing principles that repeatedly govern how studies are framed. In others, the literature remains distributed across several substantial branches, indicating the coexistence of multiple technical directions within the same macrotopic. Intermediate cases preserve clear high-level divisions while still supporting meaningful internal specializations. Together, these results show that the internal architecture of the GO field is not uniform across applications but instead reflects the specific mechanistic, experimental, and conceptual axes that define each research area. This domain-dependent hierarchy strengthens the central conclusion of our literature-scale analysis: the full-text topic structure reveals application-specific signatures that would be obscured in a flatter or more uniform view of the GO literature.

### Parameter Extraction

#### Co-Occurrence Communities

Although hierarchical topic analysis reveals the conceptual organization of the existing GO literature, it does not explicitly capture the variability of experimental practices across different domains. This distinction is important for materials discovery because two articles can belong to the same macrotopic while reporting very different combinations of synthesis variables, material descriptors, characterization settings, and performance metrics. To derive reusable data-curation and modeling guidance from the literature, we need to understand which experimental parameters are commonly reported together and how these coreporting patterns change across application fields.

We performed information extraction using an LLM guided by a structured prompt template containing a proto-ontology (Section C.1 in the SI).^46^ Here, we analyzed all eight GO applications. The analysis used 383,135 extracted parameter rows from comparable topic-level extraction sets. A proxy validation of the evaluated extraction rows (284,453 rows) showed that 97.1% of extracted values were grounded in the source text, 98.5% of evidence snippets were grounded, and 95.7% of rows were fully grounded (Section C.4 in the SI).

Manual validation using human-annotated data indicates that the precision of the LLM-based extraction is high across all topics (≈92%). This suggests infrequent factual hallucinations occurred during the extraction process, such as the creation of nonexistent parameters or the assignment of incorrect values. In addition, the recall is ≈96% and the F1 score is ≈94%, suggesting that most of the parameters were correctly recalled. This interpretation is supported by the sampling-sufficiency analysis, which showed that the dominant parameter rankings and strongest coreporting edges remained stable at the sampling size used in this study (7569 articles; Section C.2). Therefore, the extracted parameter communities should be interpreted as conservative maps of the main experimental reporting patterns, rather than exhaustive inventories of all reported parameters.

Parameter co-occurrence analysis reveals distinct experimental signatures across the application domains. Chord plots illustrating recurrently displayed parameters for each domain (Figure 5) show that the GO literature is organized not only by the application studied but also by how experiments are assembled. It represents the top 125 parameters from each application domain, yielding 558 unique recurrent-parameter nodes. The extracted parameters span synthesis conditions, processing histories, material state descriptors, characterization settings, assay or device conditions, and functional responses. Some of these parameters are broadly shared, such as oxidation conditions, sonication, drying, concentration, particle size, and oxygen content descriptors. Biomedical & Drug Delivery is characterized by a biointerface and cell assay configurations containing parameters such as *zeta_potential*, *synthesis_ph*, *cell* *_seeding* *_density*, *glutaraldehyde*_*concentration*, *fixation* *_time*, *fbs_* *concentration*, and *cell* *_viability*. Water treatment and membrane separate adsorption parameters, including *adsorbent*_dosage, *mb*_*concentration*, *ph*_*range*, *reusability*_*cycles*, *removal*_*efficiency*, and *adsorption*_*capacity*, from membrane-transport parameters such as *water*_*contact*_*angle*, *effective*_*membrane*_*area*, *filtration*_*pressure*, *membrane*_*thickness*, *salt*_*rejection*, and *water*_*flux*.

**Figure 5:**
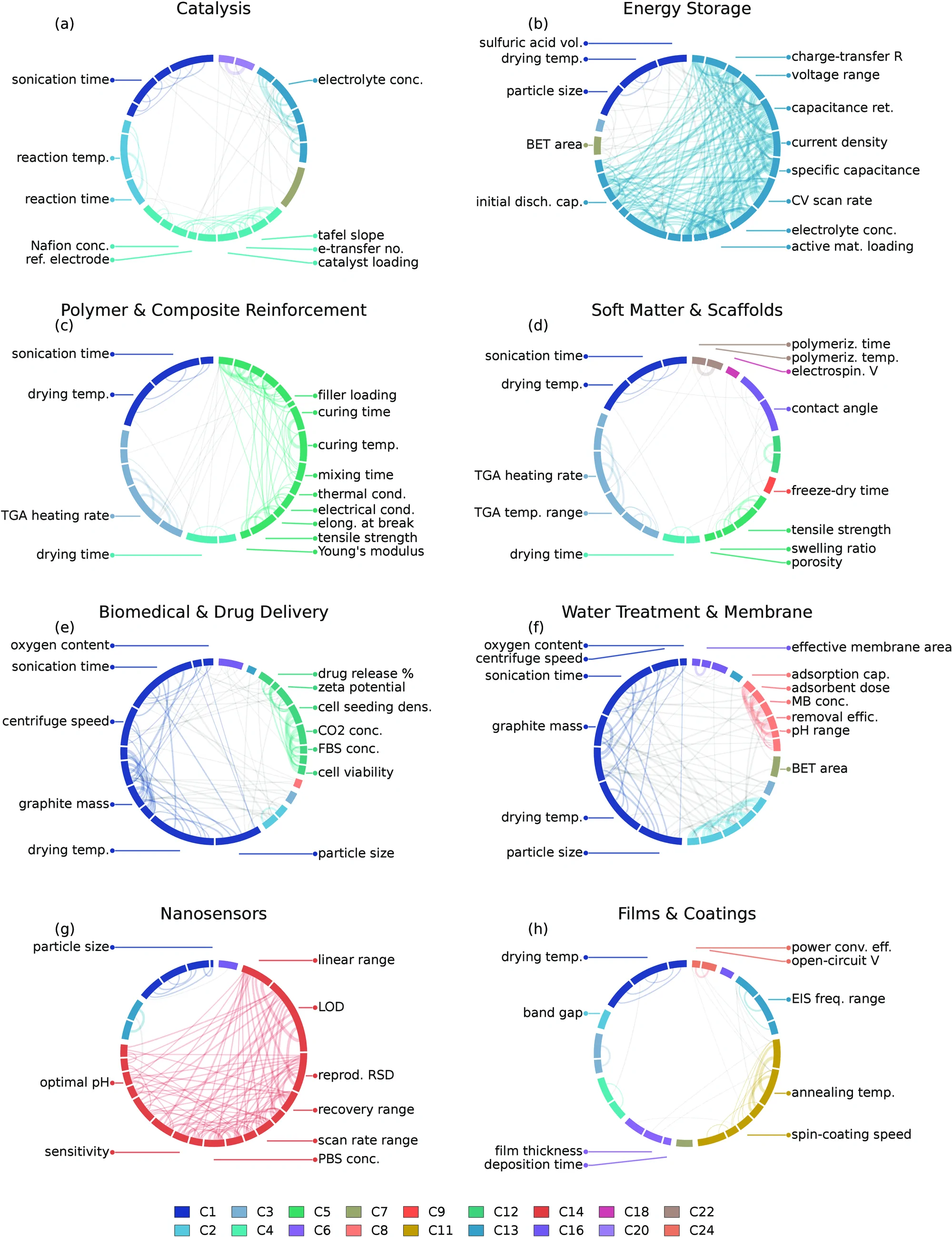
Domain-specific parameter co-occurrence communities across the GO literature. Nodes represent parameters, node size is proportional to independent-article frequency within the displayed domain, and node color indicates membership in the single global all-topic community partition (C1–C26). Edges represent parameter pairs that are statistically enriched for coreporting across independent articles.

The same structure appears in the other domains. Energy Storage concentrates around electrochemical testing and electrode/device descriptors, including *electrolyte*_concentration, *cv*_scan_*rate*, *voltage*_range, *current*_density, *active*_material_*loading*, *charge*_transfer_*resistance*, *specific*_capacitance, and *capacitance*_retention. Nanosensors emphasize calibration and assay performance through *limit*_*of*_*detection*, *linear*_*range*, *sensitivity*, *reproducibility_rsd*, *recovery_range*, *pbs_concentration*, and *optimal_ph*. Catalysis is dominated by reaction and electrocatalytic descriptors such as *catalyst_loading*, *reference_electrode*, *nafion*_c*oncentration*, *stability*_test_duration_,_ *tafel*_*slope*, and *electron* *_transfer_number*. Polymer & Composite Reinforcement articles report processing and structure to mechanical/electrical responses through *curing*_*temperature*, *curing*_*time*, *mixing*_*time*, *tensile*_*strength*, *youngs*_*modulus*, *elongation*_*at*_*break*, *porosity*, and *electrical*_*conductivity*. Soft matter and scaffolds and films and coatings further resolve freeze-drying, polymerization, electrospinning, annealing, spin coating, deposition, device geometry, and corrosion performance settings. These communities, therefore, recover the operational logic of experiments: what material state was prepared, what experimental environment was imposed, and what response was measured.

Across all domains, we identified a community, denoted as C1, that is characterized by broadly shared GO preparation parameters or common characterization. For a topic fingerprint, the other communities are more informative than the broad parameter community. C1 contains parameters such as *graphite*_*mass*, *hydrogen*_*peroxide*_*volume*, *sulfuric*_*acid*_*volume*, *potassium*_*permanganate*_*mass*, *sodium*_*nitrate*_*mass*, *drying*_*temperature*, *sonication*_*time*, *particle*_*size*, and *centrifugation*_*speed* (Figure S. 23 in the SI). These variables describe GO material synthesis configurations that are present across all of the domains.

The topic-community fingerprints show that each application area is a mixture of shared and specialized reporting programs rather than a single isolated module (Figure 6). Water Treatment & Membrane is strongly represented by molecular adsorption (C8), surface characterization (C7), optical response and reaction-condition descriptors (C2), and membrane transport (C16). Biomedical & Drug Delivery is centered on the biointerface, formulation, surface-charge, and cell-assay community (C12), but it also shares transport descriptors with water-treatment and membrane studies. Energy Storage is dominated by electrochemical testing and hydrothermal synthesis descriptors (C13), while Catalysis combines the reaction-condition community with electrochemical and photocatalytic/optical descriptors (C2, C4, and C13). Nanosensors is enriched in calibration and assay-performance parameters (C13 and C14). Polymer & Composite Reinforcement draws strongly on mechanical/curing configurations (especially C5 and C3, with C10 as a secondary processing community likely for composites). Soft Matter & Scaffolds is more associated with mechanical and structural (C5) and freeze-drying/formulation descriptors (C9), whereas Films & Coatings is more associated with film-growth, deposition, and annealing (C11) and hydrochemical processing (C4).

**Figure 6:**
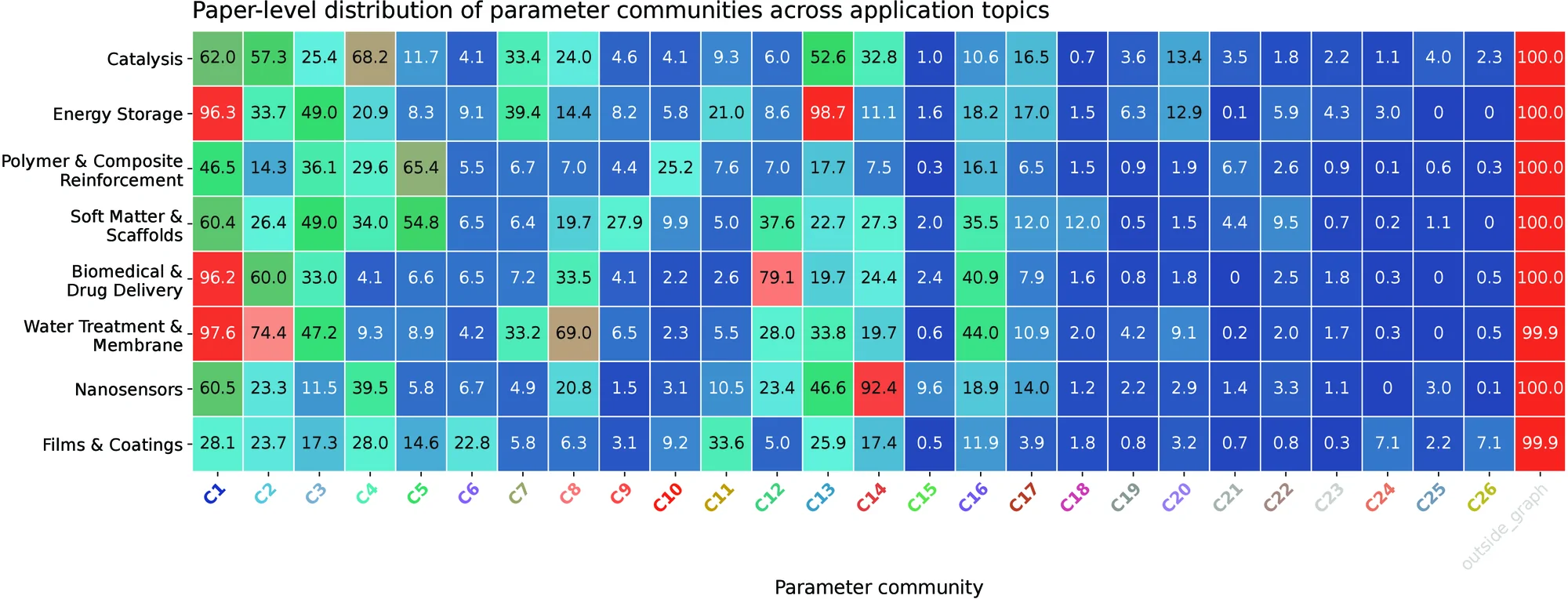
Percentage distribution of articles across parameter communities and LDA topics. Rows show LDA-derived application topics and columns show global parameter co-occurrence communities. Each cell reports the percentage of articles on that topic containing at least one extracted parameter from the corresponding community. The *outside*_*graph* column indicates articles containing extracted parameters outside the retained community-graph node set.

C1 (as well as C2 and C13) and the community fingerprints (e.g., C5, C6, and C9) play complementary roles. C1 defines the cross-domain GO foundation: oxidation chemistry, exfoliation, drying, particle dimensions, and descriptors that appear throughout the literature and do not belong to one application alone. In contrast, many highly specific parameters occur only in narrower experimental contexts or in specialized subfields. These local descriptors do not necessarily form a cross-shared community; therefore, they are decisive for interpreting a particular experimental program. For example, C5 encodes a mechanical experimental program and is shared between Polymer and Composite Reinforcement and Soft Matter and Scaffolds topics. Additionally, specific parameter co-occurrences explain why the communities are scientifically interpretable. In biomedical studies, *cell*_*seeding*_*density*, *co2*_*concentration*, *fbs*_*concentration*, and *cell*_*viability* co-occur because the cellular response to GO depends on culture density, incubation environment, serum content, and assay readout. *fixation*_*time* and *glutaraldehyde*_*concentration* co-occur because fixation quality depends jointly on exposure time and cross-linker concentration. In molecular adsorption/removal studies, *adsorbent*_*dosage*, *solution*_*ph*, *contact*_*time*, *mb*_*concentration*, *removal*_*efficiency*, *adsorption*_*capacity*, and *reusability*_*cycles* appear together because they define the uptake capacity, process efficiency, and cycling stability. In membrane studies, *water*_*contact*_*angle*, *effective*_*membrane*_*area*, *filtration*_*pressure*, *membrane*_*thickness*, *salt*_*rejection*, and *water*_*flux* jointly describe wetting, transport area, driving force, barrier thickness, selectivity, and permeability. Similar interpretive links appear in energy storage, where *electrolyte_concentration*, *cv*_*scanrate*, *current_density*, and *specific*_*capacitance* define the electrochemical testing state, and in nanosensors, where *limit*_*of_detection*, *linear_range*, *sensitivity*, *recovery_range*, and buffer conditions define analytical performance. The communities are not merely arbitrary graph clusters; instead, they reflect the physical, chemical, biological, and device level dependencies that organize experimental reporting.

Finally, we can conclude that the parameters observed in the literature are organized into three distinct structural layers. The first layer, represented by C1, is a cross-domain experimental program. It provides the common GO language needed to compare studies that differ in application but share similar synthesis and characterization practices. The second layer consists of domain-specific experimental configurations, such as adsorption/removal, membrane transport, biointerface assays, electrochemical storage, sensor calibration, catalysis, mechanical testing, and film/coating/device fabrication. These modules encode reporting-derived design signatures: which variables are normally controlled together and which responses are normally reported with them. The third layer consists of subtopic-specific niches characterized by *outside_graph*.

#### In-Depth Parameter Analysis

The prompt used for extraction contains within its template a protoontology that enables certain reasoning capabilities for the LLM model used. For example, the model is instructed to associate each numerical parameter of a measurement with the material to which it refers. We use the term protoontology here because these definitions are specified at the level of natural language text and are consequently subject to the in-context learning capabilities of the pretrained LLM. A fully developed ontology, by contrast, could be defined through a specific data structure such as the Resource Description Framework (RDF) triples. However, this would require exhaustive modeling that is highly dependent on the target domain. Below, we analyze these results by considering the extracted compounds along with three numerical parameters selected for the demonstration.

##### Material Taxonomy Categorization

GO-related materials comprise a heterogeneous family of materials, including graphite oxide, pristine GO, few-layer GO, rGO, and GO-based composites. Each material class may exhibit different physicochemical properties and an application-dependent performance. Although the prompt explicitly guided the LLM model to identify and classify the GO-based material category reported in each article, the extraction produced a very large number of distinct material-related terms, reaching tens of thousands of variants (Figure S.19 in the SI). This high terminological variability indicates that the model captured the material descriptions present in the text. However, they were unable to fully consolidate them into a stable and consistent set of GO-based material categories. To address this limitation, we applied a material classification strategy based on a well-established ontology of the GO-related engineered materials. This procedure allowed us to reduce the terminological variability produced during extraction and obtain a more consistent classification of the materials used in each article, enabling the subsequent analysis of material-category distributions across the LDA-derived topics, as shown in Figure 7.

**Figure 7:**
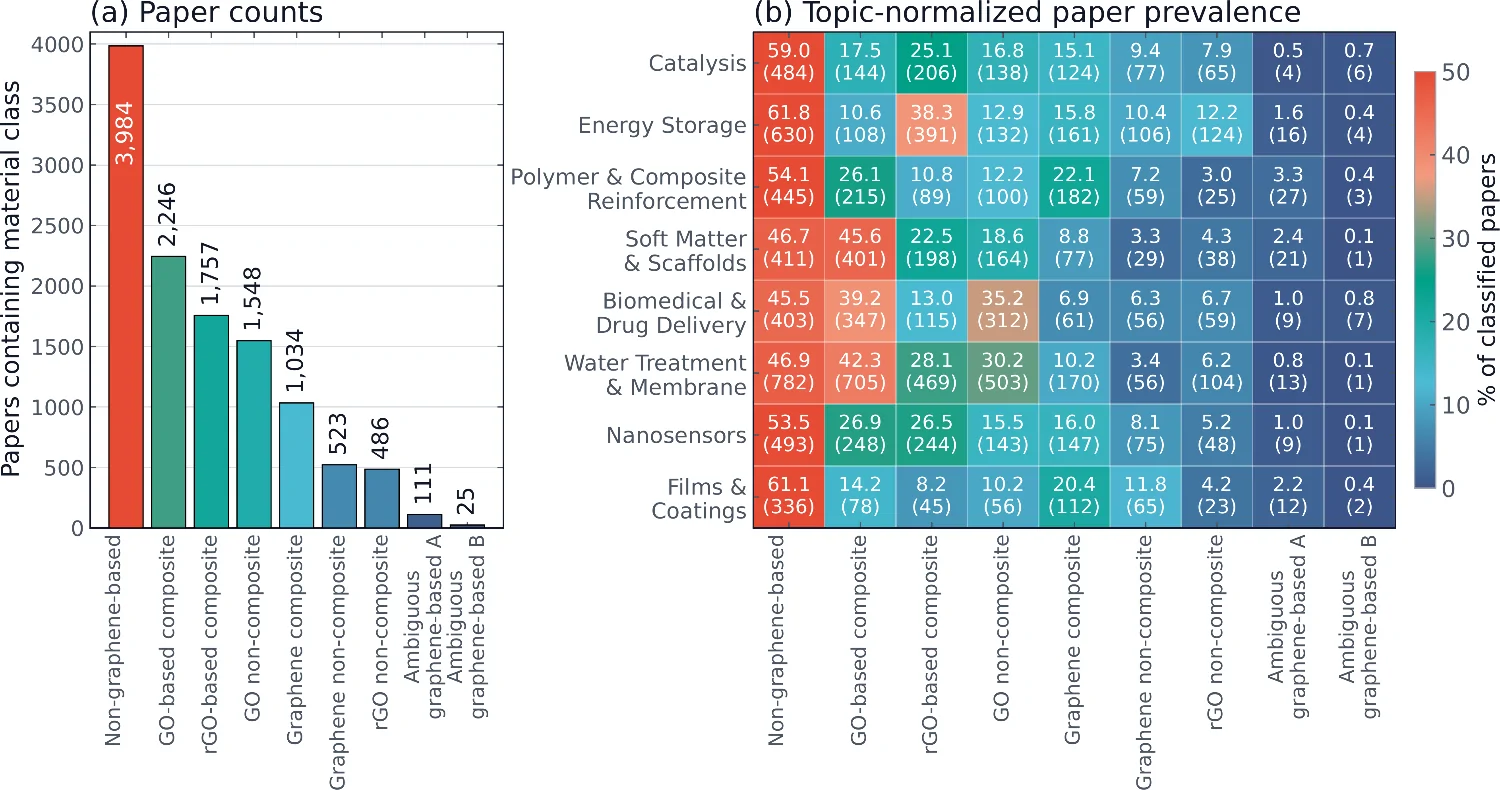
Material distribution across all eight topics. Panel (a) reports the total number of articles containing at least one material in each category. Panel (b) reports topic prevalence. An article contributes once to each extracted materials category, and each individual material entry is assigned to exactly one category. Cell labels show the percentage of classified articles and the corresponding article count in parentheses. “Ambiguous graphene-based A” is a graphene-based material with family unspecified, while “ambiguous graphene-based B” is a graphene-based material with family and composition unspecified.

The material-class distribution shows that the most frequent category is nongraphene-based materials, exceeding all graphene-family classes (Figure 7a). This large count is expected because many graphene-based material articles do not report only graphene, GO, or rGO as isolated products; instead, they describe composite and hybrid systems in which graphene-family materials are combined with other components. These nongraphene-based materials include polymers, metallic and inorganic nanoparticles, oxides, membranes, scaffolds, and other matrices or active phases that are synthesized, modified, or incorporated alongside the graphene component. Therefore, it reflects the strong prevalence of hybrid material designs in this literature. This result also supports the need for a taxonomic classification that separates graphene-family materials from associated nongraphene components, since both are central to understanding the material systems reported in the articles.

Each topic represents only one specific material type. GO-based composites are particularly visible in Biomedical & Drug Delivery (39.2% of classified articles), Water Treatment & Membrane (42.3%), and Soft Matter & Scaffolds (45.6%). Energy Storage is enriched in rGO-based composites (38.3%), consistent with electrochemical applications that often require conductive, reduced, or partially reduced materials. Nongraphene-based materials are the most predominant among the materials produced in the articles, including metallic nanoparticles, polymers, electrolytes, substrates, and devices (Figure 7b).

As observed, the majority of the GO-related literature focuses on composite materials or nanocomposite systems (Figure 7). Studies associated with the topics of Soft matter & Scaffolds, Biomedical & Drug Delivery, and Water Treatment & Membranes exhibit a greater prevalence of GO. In contrast, pure graphene appears more prominently in the Films & Coatings and Polymer & Composite Reinforcement literature. These findings highlight the particularities of each subfield with respect to the nature of GO and other carbon-based materials employed in the respective studies.

After stratifying the data by GO-based material type, we evaluated the similarity of parameter co-occurrence patterns within each application topic (Section C.7 in the SI). We observed that the topics “Energy Storage”, “Biomedical & Drug Delivery”, and “Water Treatment & Membrane” exhibit similar parameter co-occurrence patterns across several stratified GO-based materials, including GO noncomposites, GO-based composites, rGO noncomposites, rGO-based composites, and graphene composites and noncomposites. On the other hand, topics “Catalysis”, “Polymer & Composite Reinforcement”, “Soft Matter & Scaffolds”, and “Nanosensors” have similar parameter co-occurrences with specific GO-based materials, while topic “Film & Coatings” shows lower similarity of parameters co-occurrence with many of the stratified materials (Figure S.25).

By combining LDA-topic modeling, material taxonomic classification, and parameter community determination, we can develop a parameter-set recommendation model (Figure 8). Since parameter communities encode fingerprints of the research topics and material classes investigated, we can leverage high-level information about the intended research topic and material class to recommend the most relevant parameter communities (from the perspective of how the existing literature is organized). For instance, if a researcher intends to study rGO for Energy Storage applications, the system can identify the most relevant parameter communities as C13, C1, C14, C4, and C2. Some of these communities are cross-topic (C1 and C13), while others are more topic-dependent (C14, C4, and C2). Since each community corresponds to a set of parameters directly linked to an experimental workflow, the system efficiently transfers knowledge from a large body of literature into a practical recommendation – a parameter list that the researcher can readily adopt either as a starting point or as a means of complementing and validating their own experimental protocols. These results are available as a lookup table on Github.

**Figure 8:**
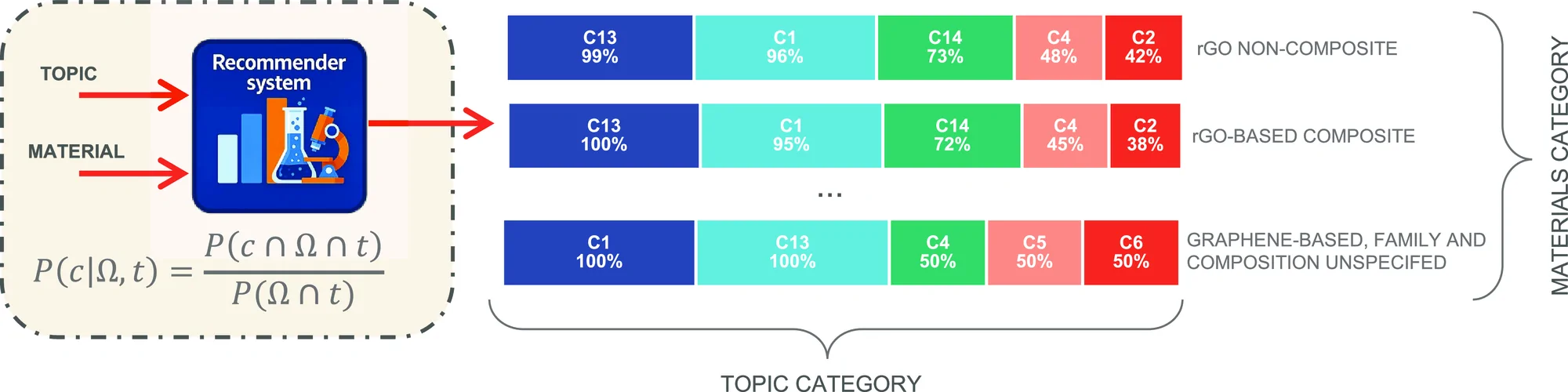
Knowledge transfer through a recommendation system. Given a topic *t* and a material category Ω, we can retrieve the relative frequency of articles reporting specific parameter communities *c*. Relevant communities can then be used to recommend sets of parameters (properties, characterizations, etc.).

Because the LDA-based topic separation primarily identified application-oriented domains in GO research, the resulting recommendation system focused on the application areas of GO-based materials. In practical terms, it can suggest which experimental, structural, physicochemical, or performance-related parameters communities are most likely to be relevant for a selected material class within a selected application topic. This enables the transfer of information across topics and material categories, supporting the identification of important reporting and characterization parameters in less-explored combinations of GO-based materials and applications. Finally, it is also possible to use this approach, especially the parameter community modeling, to improve proto-ontologies for future LLM-based information extraction and structure final ontologies for better database construction and representation.

##### Numerical Entities

Similar to material names, the extracted parameters also exhibit long-tailed term frequency distributions (Figure S.28 in the SI). In this case, however, we did not perform a prior knowledge-based normalization. In fact, it is precisely the heterogeneity of these terms that enables the discovery of distinct experimental configurations within each GO literature topic. Since this issue was addressed from a semantic perspective during the community analysis stage, we focus here on the range of numerical values that specific parameters may assume. We selected only three parameters to illustrate their topic dependence, namely, sorption capacity, BET surface area, and specific capacitance. The distributions of these values are presented in Figure 9. Although the merged term obtained after cosine-similarity-based postprocessing corresponds to adsorption capacity, we opted to use the broader term sorption because it encompasses both adsorption and absorption processes. This choice reflects the possibility that the models treated these properties as semantically equivalent because of their contextual similarity in the contexts in which they appear and the physical units they share. Moreover, in many cases, the exact physical mechanism was not explicitly specified or fully resolved in the source article.

**Figure 9:**
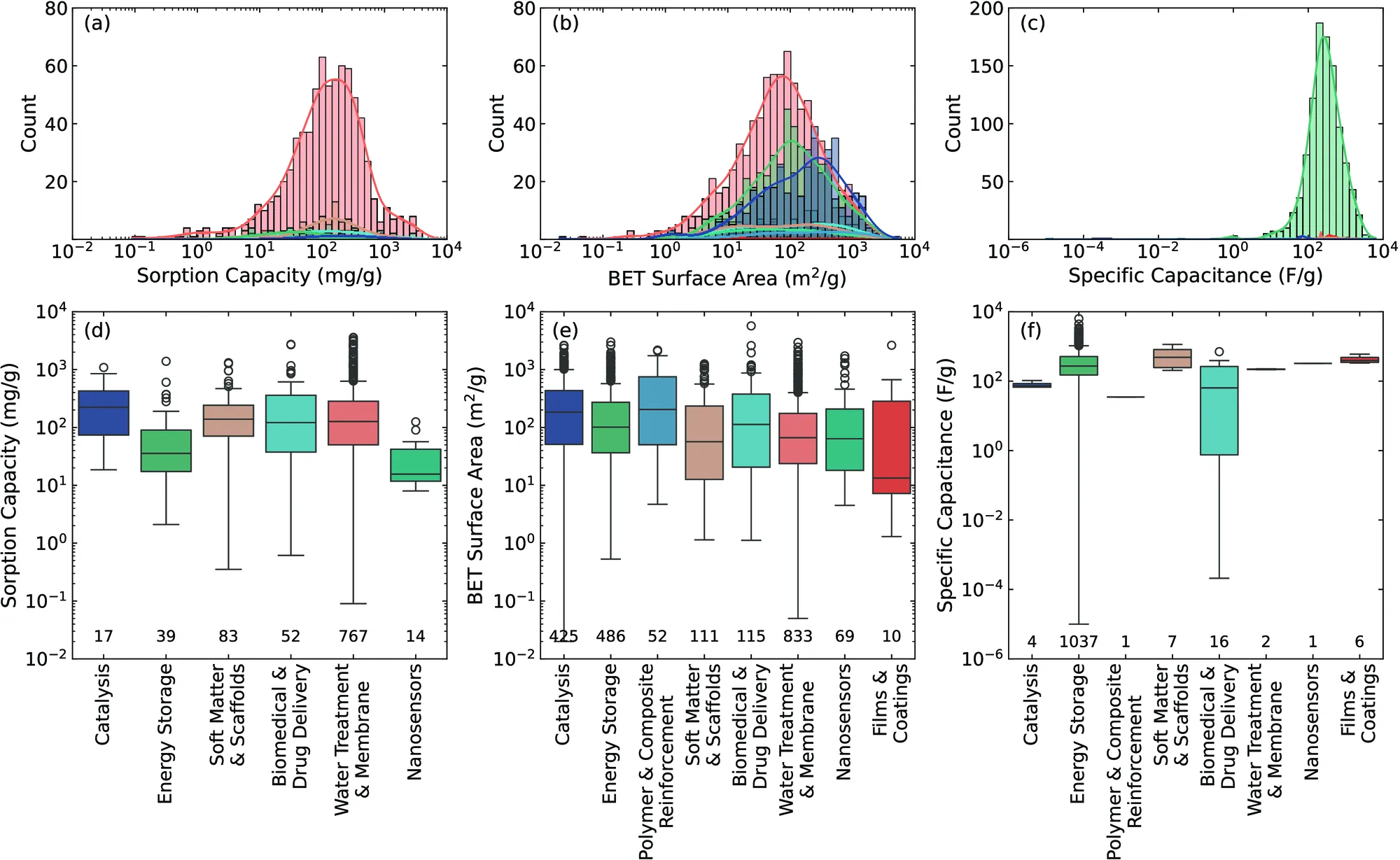
Distribution of numerical values for selected parameters. (a–c) Histograms of sorption capacity, BET surface area, and specific capacitance are shown in panels. Panels (d–f) present the distributions of sorption capacity, BET surface area, and specific capacitance as a function of the LDA topic. The numbers indicate the quantity of extracted parameter instances contained in the distributions for each topic.

We selected a subset of extreme sorption-capacity values for manual validation, confirming that the LLM correctly extracted both the numerical values and their corresponding units (section C.4 in the SI). This parameter was selected to illustrate inherent complexities. The *Adsorption Capacity* is influenced not only by the adsorbent material but also by the characteristics of the adsorbate. As shown in Figure S.21a, some extracted materials provide information about both components. For example, the designation *GO/1-OA for MG* indicates that the adsorbent is a GO composite with a tetrazolyl derivative and octadecylamine (OA), while the adsorbate is malachite green (MG).^47^ In other cases, additional information is extracted along with the adsorbent, including nanostructural characteristics, isotherm models, and solution pH. Indeed, the reported *adsorption capacity* may refer to *q*_*max*_ from an isotherm model (e.g., Langmuir or Freundlich) or the highest measured value under the tested conditions, denoted max­(*q*_*e*_), at a given time. Other pertinent data may include the type of kinetic model used (e.g., pseudo-first-order, pseudo-second-order, or intraparticle diffusion), the process temperature (relevant for thermodynamic or isotherm models), and the fitting parameters for these models. Essentially, extracting adsorption capacity values alone is not sufficient to fully characterize the phenomena of interest.

One potential solution to improve information extraction from LLMs is to refine prompts to explicitly specify which properties to retrieve with a precise terminology. However, deploying this approach on the large scale of text mining (120,000 articles) can be computationally intensive, and the informational yield may be limited, as only a fraction of the corpus contains data related to adsorption. Therefore, the trade-off should be considered at the level of the topics or subtopics. Focusing on outliers can reduce the search space and highlight extreme cases that may indicate opportunities for optimization and further investigations. Furthermore, these outliers can represent anomalies associated with emerging insights that have yet to gain traction in the literature, pointing to unexplored or underdeveloped research avenues.^48^

The results discussed in this section present a potential pathway to improve the size, diversity, and completeness of data set construction. It is important to clarify that our objective is not primarily to focus on the mechanics of parameter extraction but to show that literature-scale modeling of GO can provide the organizational structure needed to make such extraction more accurate, specific, and computationally tractable. This challenge is especially acute for GO because, unlike some inorganic materials that possess specific chemical structures and compositions, GO is highly heterogeneous, and its experimental information is reported with limited standardization, inconsistent terminology, and variable granularity. Under these conditions, ontology development is not only helpful but also necessary since reliable extraction depends on understanding how parameters, materials, and experimental descriptors relate across diverse contexts. Recent work has shown that LLMs encode substantial material knowledge and can support prediction and data-augmented synthesis planning.^49,50^

The discussion on adsorption capacity represents only one example of how the methodology proposed in this work can be applied to mapping the GO literature.

### Predictive Model

Integrating topic modeling and LLMs into a data extraction pipeline enables the development of structured data sets with a range of associated parameters. For example, it is possible to develop a table that includes the adsorption capacity alongside relevant parameters, such as the adsorbent, adsorbate, pressure, and temperature. Such structured data sets are highly valuable because they serve as both indexed knowledge and training data for machine learning (ML) models. These models could support the rational design of GO materials by enabling the prediction of target properties based on the novel combinations of input variables. For example, a materials engineer may be interested in assessing the extent to which adsorption capacity varies with pressure and temperature for a given adsorbent–adsorbate pair. Thus, the trained model could predict the adsorption capacity for new pressure and temperature conditions relevant to those pairs.

At a first level, the model can operate as a data-driven interpolator; i.e., the model draws from the extracted data set, allowing predictions between variables such as pressure and temperature and among new adsorbent–adsorbate combinations, as long as these inputs remain within the applicability range of the curated data. Consequently, experimental efforts can be directed toward testing the conditions prioritized by the model, thereby minimizing the need for empirical trial and error. At a second, more ambitious level, the model may support extrapolation beyond the immediate boundaries of the training data, helping identify promising materials, operating conditions, or adsorbent–adsorbate pairs that have not yet been experimentally explored. However, this extrapolative use is substantially more challenging, since predictions in poorly represented regions of the feature space are more vulnerable to bias, spurious correlations, and loss of physical meaning. Therefore, extrapolation requires careful assessment of model uncertainty, explicit definition of the applicability domain, and, ideally, integration with mechanistic knowledge or targeted experimental validation.

Some of these concepts have previously been explored in the adsorption literature. For example, Rashidi et al. utilized ML models trained on experimental data at various concentrations of adsorbents, pH levels, and contact times. However, their study was limited to the adsorbent GO/gallic acid (GO/GA) and silver particles as the adsorbate.^51^ Similarly, Al-Jamimi et al. trained models for the adsorption of acid red 1 dye and bisphenol A using polyethylenimine/GO/layered triple hydroxide as the adsorbent, yet their data set was limited to only 29 samples.^52^ Fathalian et al., in turn, studied different GO-based adsorbents for CO_2_ adsorption using 895 data points manually extracted from 17 articles in the literature, training ML models to predict adsorption capacity as a function of surface area, pore volume, temperature, and pressure.^53^ Also studying CO_2_ adsorption using data manually extracted from the literature, Zhu et al. focused on porous carbon-based adsorbents, compiling approximately 6244 data points for 155 different carbon materials.^54^ They emphasized the need to construct more comprehensive data sets to develop improved ML in future research.

Here, a CO_2_ adsorption case study provides a downstream test of this interpretation, as shown in Figure 10. We first used the GO topic/community map to locate the CO_2_ adsorption subliterature. This set contained 8 subtopics, concentrated mainly in water treatment and adsorption and surface characterization communities, with synthesis and material chemistry communities used to recover additional descriptors (section D in the SI). As can be seen in Figure 10a and Table 1, the 5-fold random-split CV strategy achieves the highest performance. This occurs because random splitting increases the likelihood that certain data ranges are represented as interpolation rather than as extrapolation to new observations. For the leave-one-DOI-out cross-validation, the data associated with each individual DOI were used exclusively as the test set, while all remaining data were used for training (Figure 10b). This procedure was repeated for all 37 DOIs in the data set. The rationale behind this approach is that each individual DOI may represent out-of-distribution (OOD) data with respect to the training set. Indeed, the predictive performance of the algorithm decreases in this setting when all predictions are considered collectively. However, the performance can vary substantially across individual papers, since a given paper may actually be in-distribution (ID) with respect to the training set (Table S.9 in the SI). This indicates that, while some studies propose new materials that are OOD with respect to the remainder of the data set, others reproduce previously reported materials/experiments or introduce only minor variations relative to materials/experiments that have already been reported.

**Figure 10:**
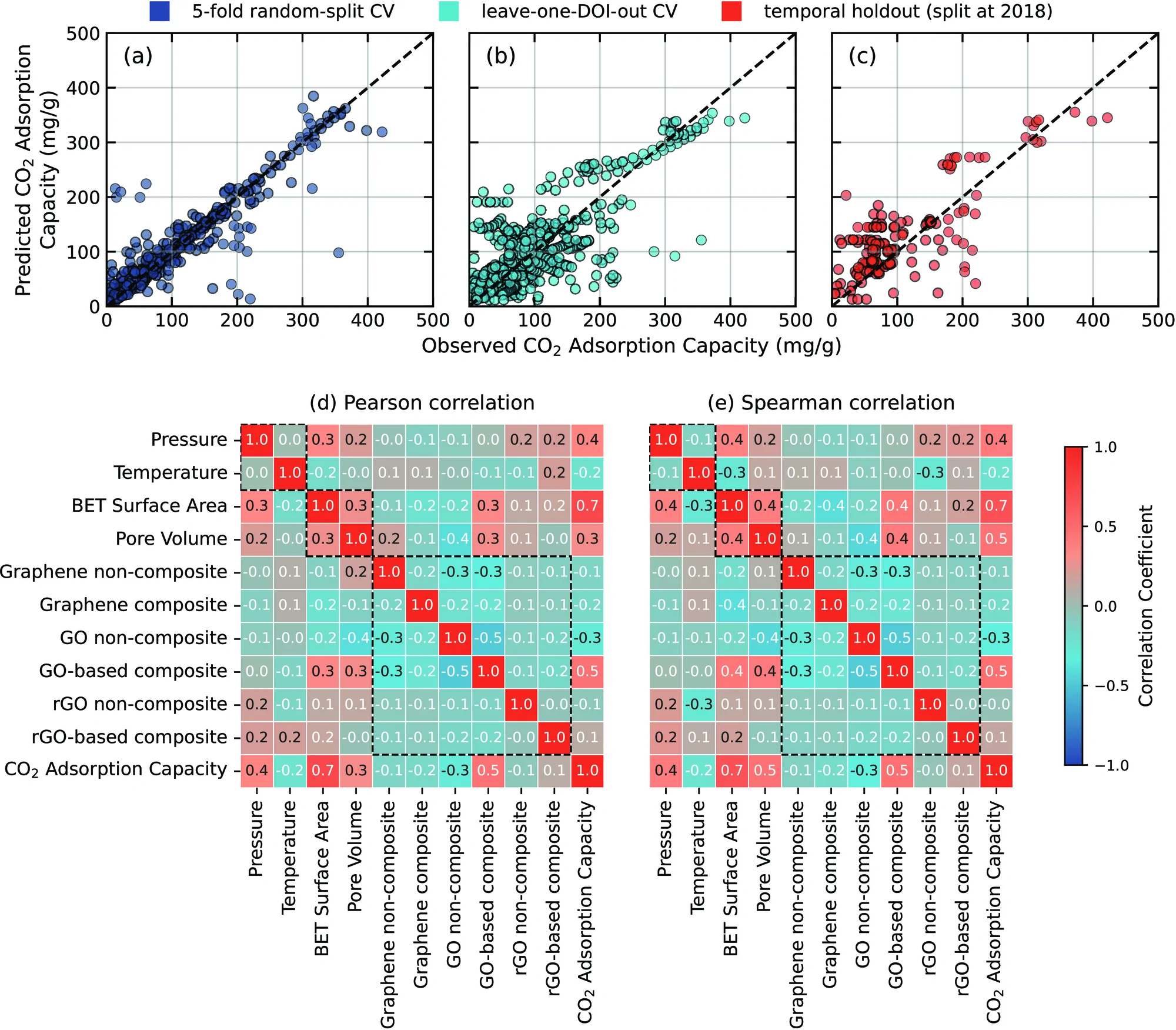
Performance evaluation of ExtraTrees-based predictive models. (a) Train-test splitting using 5-fold random-split cross-validation (CV). (b) Train-test splitting using leave-one-DOI-out CV. (c) Train-test splitting using temporal holdout (article year for training ≤ 2018; test >2018). (d) Pearson and (e) Spearman correlation between features of the data set used to train the predictive models. Along the diagonal, from top left to bottom right, three black dashed boxes separate features related to experimental thermodynamic conditions, morphological properties of the adsorbent material, and chemical descriptors of the adsorbent, respectively.

**Table 1:** Performance Metrics for ExtraTrees-Based Predictive Models^a^

Test Stratification	*R* ^2^	MAE (mg/g)	MAD: MAE	RMSE (mg/g)	STD:RMSE
5 fold random-split CV	0.856	10.8	5.0	28.1	2.6
leave-one-DOI-out CV	0.609	31.5	1.7	46.4	1.6
temporal holdout (split at 2018)	0.542	44.4	1.5	58.4	1.5

a *R* ^2^, MAE, MAD, RMSE, and STD represent the coefficient of determination, mean absolute error, mean absolute deviation, root mean squared error, and standard deviation, respectively. The metrics were calculated using the concatenated *y*_test_ and *y*_predicted_ vectors across all splits within each test stratification.

We also performed a train-test split based on the publication year of each paper (temporal holdout; Figure 10c). All papers published up to and including 2018 were assigned to the training set, while all papers published after 2018 were assigned to the test set. This resulted in an approximately 75%/25% split between training and test data, considering all instances in the data set. The purpose of this strategy is to enforce the ultimate criterion for temporal generalization: any ML model should be restricted to the data available at training time, and its applicability should be assessed by its predictive performance on future data. This split does not guarantee the absence of ID samples, since, as in the previous strategy, materials/experiments may be repeated in the literature at different points in time, thereby increasing redundancy within the data set.

It is important to note that distinguishing between the OOD and ID data is not a trivial problem. Here, we discuss two strategies to more robustly assess model performance in scenarios in which generalization becomes more challenging. We could also consider the OOD and ID with respect to specific materials or with respect to each individual feature. In the latter case, a statistical test could be used to assess whether an instance belongs to each of the individual training distributions. However, it would still be necessary to determine whether an instance should be classified as OOD only when all feature values fall outside the corresponding training distributions or whether a single feature falling outside its training distribution would be sufficient to classify the instance as OOD. What constitutes interpolation, extrapolation, or redundancy in the feature space of a predictive problem remains an active topic of discussion in materials informatics research.^55^ Moreover, previously reported ML studies on CO_2_ adsorption prediction do not clearly address this issue,^53,54^ likely relying on a validation strategy similar to the 5-fold random-split cross-validation employed in this work.

A clear differentiation must be done between *patterns reported in the literature*, *predictive interpolation within the represented data distribution*, *generalization to genuinely unseen materials*, and *mechanistic structure–property understanding*. This distinction is particularly important for models constructed from published experimental data because the literature is not a uniform sampling of materials space: unsuccessful experiments are systematically underreported, and historical experimental choices concentrate research within particular materials, synthesis routes, and conditions.^56,57^ The main limitation is that predictive accuracy within a literature-derived data set cannot, by itself, demonstrate extrapolative materials discovery. Recent materials-informatics studies have shown that distribution shift can substantially reduce performance on newly encountered materials,^58^ that similarity and redundancy between training and test data can lead to optimistic estimates of predictive performance,^59,60^ and that nominal out-of-distribution evaluations may still primarily measure interpolation within regions already represented by the training data.^61^ Similar considerations apply to literature-derived CO_2_ adsorption models, which have demonstrated strong predictive utility across porous carbons, MOFs, and graphene-oxide-based adsorbents,^62–64^ but remain constrained by the materials, experimental conditions, and descriptors represented in the underlying literature. Recent data sets have explicitly highlighted literature selection bias, heterogeneous experimental protocols, incomplete reporting, and restricted chemical coverage as important limitations to generalization.^65^

The data set used here to train these predictive models for CO_2_ adsorption included five features, four physically relevant ones that are widely reported, and one additional feature to account for the chemical characteristics of the adsorbent (Table S.8 in the SI). Only instances in which the adsorbent belongs to a family containing GO or graphene were included in the data set, according to the material taxonomy classification (Section C.5 and Figure S.26 in the SI). The prediction problem can be physically interpreted as follows: the features pressure and temperature address external thermodynamic conditions that are controlled during the experiment; the features BET surface area and pore volume describe morphological properties of the tested adsorbent material; and the feature material_taxonomy encodes a chemical description of the tested adsorbent material. Features representing external conditions do not show any correlation among them, since these variables are arbitrarily controlled during the experiment (Figure 10d and 10e). It can also be observed that an increase in pressure tends to increase the CO_2_ adsorption capacity (positive correlation), whereas an increase in temperature tends to decrease it (negative correlation). Although the group of morphological descriptors shows positive correlations with one another, this does not imply a simple linear relationship. Both features show a positive correlation with the adsorption capacity (Figure 10d and 10e). This trend is physically plausible for surface area since a larger accessible surface can increase the number of interaction sites available for CO_2_ adsorption. In contrast, the role of pore volume is expected to be more complex and not necessarily linear because its effect depends on how the pore volume is distributed across micro-, meso-, and macropores, as well as on its coupling with the accessible surface area. The relatively high Spearman correlation between pore volume and surface area suggests that, within this data set, pore volume may partly reflect broader textural characteristics of the adsorbents rather than acting as an independent linear descriptor of adsorption capacity. The internal correlations among the features in the group encoding the material should be interpreted in relation to those of the other features and the target. For example, GO-based composites show a positive correlation with the CO_2_ adsorption capacity. This is likely related to the positive correlation between these materials and the surface area. Therefore, in the data set explored, GO-based composites have a higher BET surface area and, consequently, exhibit higher CO_2_ adsorption capacity values. The opposite effect is observed for materials from the noncomposite GO family, which show a negative correlation with adsorption capacity, although with lower intensity.

In addition to this cascade approach, in which LLMs extract values and ML models perform downstream tasks on the extracted data set, it is also possible to use the embeddings learned by language models to make predictions directly in the latent space. Tshitoyan et al. were able to recover chemical knowledge about elements and compounds from material vectors learned in an unsupervised manner.^66^ Park et al. reviewed the use of these representations to generate new materials.^67^ More recently, with larger models, Venugopal et al. demonstrated capabilities across symbolic and numerical tasks.^49^ However, many challenges remain, such as the physical and chemical constraints of this space, which raise questions about how these models actually adhere to natural laws and address issues of interpretability and human explainability. For example, in the case presented here, we see that LLM-based extraction performed without an ontology and using a general prompt (protoontology) tends to produce several instances of the same parameter in a dispersed manner (i.e., different terms referring to the same concept) or, conversely, different parameters represented in the same way (e.g., maximum adsorption capacity from an isotherm model versus adsorption capacity measured under specific experimental conditions). Information linking adsorbents and adsorbates is also not always extracted jointly, even when the prompt explicitly requires materials to be associated with an extracted property. Improving these approaches remains an important direction for future research, whether through cascade-based extraction pipelines or methods operating directly in latent space. This limitation reinforces the importance of developing a dedicated ontology to support LLM-based information extraction from GO literature. Following knowledge engineering construction principles could enable automatic reasoning and produce more curated data sets.

It is also important to recognize that the application of NLP and AI, particularly LLMs, to materials science is still an emerging research area.^68^ Previous parameter extraction studies have relied on heuristic rules or ML models specifically trained for NER.^22,24,25^ In LLMs, NER arises as an emergent capability rather than as a task-specific objective. Likewise, transformer-based generative models for materials discovery have predominantly focused on crystalline inorganic materials^67,69,70^ or molecules,^34,35^ whereas our work lies at the interface between crystalline and amorphous materials, as well as between inorganic and organic systems. Similarly, training LLMs to directly predict material properties remains an open challenge.^49^ Current approaches typically require substantial constraints to achieve an acceptable performance, as predictive accuracy is still limited. The capabilities of LLMs for information extraction and summarization continue to be actively investigated in the computational literature, particularly given the potential for various forms of hallucination.^71^ We demonstrated here that the extracted information achieved high precision and high recall, indicating that a general prompt such as that used here induced very limited hallucinations and a high rate of information recovery.

Large-scale literature mining has already demonstrated that it is possible to recover latent chemical knowledge, identify meaningful material relationships, and generate hypotheses that are not readily apparent from individual publications.^72^ Likewise, text mining has transformed tens of thousands of synthesis descriptions into structured data sets that can be used to investigate synthesis rules and relationships across materials systems.^22,73^ These studies demonstrate that literature-derived relationships need not be causal to be scientifically useful, because they can identify recurring associations, prioritize variables, expose underexplored connections, and provide hypotheses for subsequent mechanistic or experimental investigation. Recent GO bibliometric work has demonstrated the value of mapping publication trends, keywords, and research themes across more than 14,000 publications.^74^ Our framework addresses a different and complementary question by asking how *materials, applications, experimental parameters, and characterization descriptors are connected within the full scientific record*. Instead of simply identifying frequently occurring keywords, the framework resolves research topics, material classes, parameter co-occurrence networks, and parameter communities, allowing relationships distributed across a highly heterogeneous literature to be examined systematically. The resulting map provides a data-driven representation of how GO has been synthesized, characterized, modified, and studied across research domains. The CO_2_ case study is, therefore, presented as a downstream proof-of-concept of the *utility of the literature-derived representation*. Specifically, it asks whether parameters identified from large-scale patterns in the GO literature can be translated into a structured property data set and whether those literature-prioritized descriptors remain informative across held-out publications. Finally, prospective synthesis and validation remain necessary to establish true extrapolative materials discovery, as demonstrated by closed-loop and autonomous materials workflows that couple literature-derived knowledge with active learning and new experiments.^75^

## Conclusion

This study presents a comprehensive map of the GO research landscape that operates at three nested levels: macrotaxonomy, within-domain hierarchy, and the structure of the reported experimental parameters. It is demonstrated that practices are not randomly distributed but rather exhibit domain-specific signatures. Full-text topic modeling resolves eight application-centered domains and shows that their internal subtopic landscapes differ in their branching depth and semantic organization. The parameter layer extends this map from literature classification to practical knowledge by showing that the reported variables in the articles organize into shared synthesis and characterization experimental designs and provide the variables needed for downstream ML modeling.

As a proof-of-concept of this framework, we demonstrate that the integration of topic modeling and LLM-assisted data extraction can generate structured, machine-learning-ready data sets from the adsorption literature. In the CO_2_ adsorption case study, the resulting data set enabled the development of predictive models that captured physically meaningful trends associated with pressure, temperature, surface area, pore volume, and adsorbent taxonomy. However, the comparison among random-split, leave-one-DOI-out, and temporal-holdout validation strategies shows that the apparent predictive performance depends strongly on the degree of similarity between training and test data. While literature-derived models can provide useful interpolation within the represented materials and experimental conditions, their extrapolative use for genuinely unseen adsorbents requires careful assessment of the distribution shift, applicability domain, uncertainty, and potential literature bias.

From an NLP perspective, topic modeling approaches, such as LDA and neural topic modeling, provide a large-scale overview useful for selecting relevant studies for further exploration by LLMs using specific prompts. Although general prompts enable cross-domain exploration, the complexity of certain features associated with measured parameters and synthesis techniques requires greater refinement to allow for more detailed extractions. Future research avenues may involve the development of ontologies to structure prompt templates and validate extractions, allowing for a more in-depth analysis of specific topics.

The framework presented here is generalizable beyond GO to other complex material systems with a large and heterogeneous literature. By systematically mapping macro-taxonomy, within-domain hierarchy, and experimental parameter structure, this approach provides a foundation for data-driven experimental design and cross-domain knowledge discovery. Finally, the pipeline includes full-text topic modeling to define the domain structure, neural topic modeling to resolve within-domain organization, and parameter communities to identify the experimental variables and outcomes that should be curated together. For GO, this reveals a literature organized not only by application area but also by recurring experimental designs that can guide more systematic data extraction, cross-domain comparison, and structure–property–performance modeling.

## Materials and Methods

### Data Collection and Preprocessing

Our workflow, Figure 1, begins with the collection of a large set of GO research articles using Application Programming Interface (API) connectors to bibliographic databases. This approach relies on information retrieval from the publishers’ search engines. Whenever possible, articles in XML format were preferred as they allow for more straightforward programmatic processing. In certain cases, PDF files were used instead. In such cases, OCR and language model-assisted extraction techniques were applied to process, standardize, and structure the retrieved texts. A detailed description of the data acquisition and preprocessing procedures is found in Section A of the SI.

### Topic Modeling Approaches

#### Latent Dirichlet Allocation for Macro-Taxonomy

Topic modeling used latent Dirichlet allocation (LDA), a technique that creates document representations as mixtures of latent topics, where each topic is a probability distribution over words.^76^ This generative framework uncovers recurring themes within the corpus, representing each article as a weighted combination of eight macrotopics. The resulting document-topic and topic-word distributions provide a structured overview of the literature. Details of model specification, hyperparameter selection, and validation are provided in detail in the SI, Section B.2.

#### Neural Topic Modeling for Within-Domain Subtopic Analysis

Within each LDA macrotopic, we applied neural topic modeling to obtain fine-grained semantic landscapes, as implemented in BERTopic.^36^ This algorithm uses transformer-based document embeddings and dimensionality reduction to identify coherent subtopics that represent specific techniques, goals, or mechanisms. The hierarchical clustering of these subtopics reveals the internal organizational structure (Section B.3 of the SI for a more detailed description).

#### Model Selection and Validation

We selected the number of LDA topics (*K* = 8) based on coherence scores *C*_*v*_, a measure of semantic consistency that correlates with human interpretability,^77,78^ along with the interpretability of the resulting topic labels. Topic separability was quantified via keyword-level Jaccard similarity between topic vocabularies. The macro-taxonomy was further inspected using t-SNE^41^ projections of TF–IDF^42^ document vectors, and within-domain hierarchy was summarized using dendrogram analysis with specified distance metrics and cut criteria. A full description of this methodology is provided in the SI, Section B.

### Parameter Extraction and Co-Occurrence Analysis

#### LLM-Based Extraction

We extracted experimental, processing, characterization, and performance parameters from 7,569 full-text articles using a structured LLM schema that specified parameter categories, naming conventions, unit handling, material associations, and evidence snippets. We sampled articles (approximately 1000 full-text articles per topic) with a high probability of belonging to that topic, making the sample size representative of that topic (more details in the SI, Section C.2). Extracted parameter names were normalized through synonym merging, embedding-assisted canonization, and unit normalization (Section C.3 in the SI), along with article-level deduplication. For community analysis, we constructed a binary article-parameter matrix and defined a topic-balanced node universe as the union of the top 125 parameters by independent-article frequency within each of the eight GO application topics. Parameter–parameter associations were counted at the independent-article level. For each pair, we calculated coreporting support, lift relative to marginal article frequencies, NPMI, and a hypergeometric enrichment *p* value with Benjamini–Hochberg correction. Edges were retained only when they satisfied support ≥ 3, lift ≥ 1.5, NPMI ≥ 0.03, and FDR *q ≤* 0.05. Weighted Louvain community detection was then applied to a sparsified version of the significant association graph. Extraction quality was assessed using a proxy validation of source grounding and numeric-value coverage. Complete prompt templates, graph settings, stability diagnostics, and validation details are provided in the SI, Section C.

#### Material Classification Taxonomy

We classified graphene-based materials extracted from the scientific articles using a two-level operational taxonomy adapted from established ontologies for GO-related engineered materials. First, each target material was described using detailed descriptors, including graphene-family identity, layer or dimensionality, chemical state, physical form or architecture, composition class, and component role, preserving distinctions such as GO, rGO, partially reduced GO, few-layer GO, graphene nanosheets, nanoplatelets, and graphene-based composites. These descriptors were then mapped to broader, mutually exclusive material categories, such as GO, rGO, GO-based composites, rGO-based composites, graphene composites, and other carbon-based systems using heuristic rules. When multiple material samples were reported in the same article, each sample was classified independently, but each individual material was assigned to only one category to avoid double-counting (details are in Section C.5 in the SI.)

#### Parameters Extraction Validation

For manual validation of the LLM-based extraction framework, we selected 40 articles corresponding to five articles from each LDA-derived topic and manually annotated all parameters related to material production (including GO, rGO, and GO-based composites, etc.), characterization, and property-related parameters. The manually extracted data resulted in a total of more than 3900 parameters that were checked with those extracted by the LLM. This manual validation was treated as the basis for the calculation of the precision and recall (details are in section C.8 in the SI).

#### Parameter-Community Recommendation

Using the taxonomic categorization of the materials reported in the articles and the topic-modeling analysis we can estimate the probability that a given parameter community occurs within a specific combination of GO-based material class and research topic. In this framework, the recommendation system receives as input a topic *t*_*i*_ ∈ {1,···,8} and a material category Ω_*j*_ ∈ {1,···,9} and returns the conditional probability *P*(*c*_*k*_ |*t*_*i*_,Ω_*j*_) that a parameter community *c*_*k*_ ∈ {1,···,32} is relevant, that is, present in that domain of the GO literature. The computed data supporting this framework are available on GitHub.

### Predictive Model

A CO_2_ adsorption prediction test used the parameter association map to identify adsorption/removal, surface-texture, and material-chemistry descriptor neighborhoods before model construction. CO_2_ uptake values were assembled from source tables and calibrated digitized isotherm curves, normalized to mmol g^–1^, and paired with descriptors available from the same source article. Before feature selection, DOI/source identifiers, raw material/name identifiers, target-derived fields, topic metadata, and scouting flags were removed. We adopted three different stratification strategies for the train-test split: a 5-fold random-split cross-validation strategy as a comparison, a leave-one-DOI-out cross-validation, and a temporal holdout. The ML models employed the ExtraTreesRegressor^79^ algorithm with five features, using a data set comprising 698 distinct instances. From the 50 articles initially identified with unique DOIs, data set instances were retrieved from 37 articles after applying the material-taxonomy classification filter to retain only entries corresponding to GO-based materials. More details are provided in the SI, Section D.

## Supplementary Material



## Notes

Data acquisition and preprocessing: indexing, download, and processing of downloaded files; topic modeling: text preprocessing pipeline, LDA modeling, neural topic modeling, LLM-assisted interpretation, and hierarchical visualization of subtopics; parameter extraction and postprocessing: LLM prompt, sampling sufficiency and stability, normalization postprocessing, extraction quality and validation, material classification taxonomy, parameter co-occurrence community construction, community stability across material categories, and manual validation; predictive model for CO_2_ adsorption capacity: data set construction, model construction, and model evaluation (PDF)
