# Supplementary material for: Experimental Design Signatures Emerge from Topic Modeling of Graphene Oxide Literature
Source: ACS Nano. 2026 Oct 17;20(43):28844–63. doi: 10.1021/acsnano.6c04548 (PMC13738791; doi:10.1021/acsnano.6c04548)
Supplement: Supplementary file 1 [file nn6c04548_si_001.pdf]

Supporting Information: Experimental Design Signatures Emerge from Topic Modeling of Graphene Oxide Literature

Henry J. Kobs,[†,§] Henrique Ferreira,[‡,§] Liu Yang,[†] Amauri J. Paula,[*,‡] Carlos E. Andrade,[¶] and Andreia F. Faria[*,†]

†*Engineering School of Sustainable Infrastructure & Environment, Department of Environmental Engineering Sciences, University of Florida, Gainesville, 32611-6580, FL, USA*

‡*Solid-Biological Interface Group (SolBIN), Aeronautics Institute of Technology – ITA, So Jos dos Campos, 12228-900, SP, Brazil*

¶*AT&T Labs Research, 1405 US HWY 206, Bedminster, 07921, NJ, USA*

§*These authors contributed equally to this work.*

E-mail: amaurijp@gmail.com; andreia.faria@essie.ufl.edu

Contents

A Data Acquisition and Preprocessing **S-3**

A.1 Indexing . . . S-3

A.2 Download . . . S-4

A.3 Processing the downloaded files . . . S-5

B Topic Modeling **S-6**

B.1 Text preprocessing pipeline . . . S-6

B.2 LDA Modeling . . . S-8

B.3 Neural Topic Modeling . . . S-14

B.4 LLM-Assisted Interpretation . . . S-19

B.5 Hierarchical visualization of subtopics . . . S-19

C Parameter Extraction and Post-processing **S-32**

C.1 LLM Prompt . . . S-33

C.2 Sampling Sufficiency and Stability . . . S-38

C.3 Normalization Post-processing . . . S-40

C.4 Extraction Quality and Validation . . . S-44

C.5 Material Classification Taxonomy . . . S-51

C.6 Parameter Co-occurrence Community Construction . . . S-57

C.7 Communities stability across materials categories . . . S-60

C.8 Manual validation . . . S-63

D Predictive Model for CO_2 Adsorption Capacity **S-66**

D.1 Dataset Construction . . . S-66

D.2 Model Construction . . . S-67

D.3 Model Evaluation . . . S-69

References **S-72**

A Data Acquisition and Preprocessing

We start by indexing articles from major databases (PubMed, Web of Science, Google Scholar, Scopus) using their Application Programming Interfaces (APIs) and Crossref. Then a journal-aware downloader (e.g., via Selenium) fetches the documents in Portable Document Format (PDF). We extract the full text from each PDF using Optical Character Recognition methods (OCR) when needed and convert it into a structured Extensible Markup Language (XML) format so that it is machine-readable.

With the full-text corpus ($\approx$ 120,000 articles), we normalize the text (lowercase, stop-word removal) and run Latent Dirichlet Allocation (LDA)[S1] to discover broad, high-level topics throughout the collection. For each broad topic, we then apply Neural Topic Modeling following the BERTopic approach[S2] to split it into finer "subtopics," capturing more specific themes and their prevalence.

We use a Large Language Model (LLM) to generate human-readable labels and short summaries for topics and subtopics. We organize them hierarchically (so related subtopics cluster together) and then perform expert materials analysis to interpret what these clusters mean for applications (e.g., sensors, water treatment), highlighting takeaways that matter to general readers.

A.1 Indexing

The initial step in building a comprehensive and high-quality corpus of GO-related literature involved identifying and indexing relevant publications from reputable bibliographic databases. Two primary sources were selected for this task: Crossref[S3] and Web of Science.[S4] Web of Science was used as the core bibliographic index due to its easy export, which is critical for future bibliometric and network-based analyzes. In parallel, the Crossref Metadata API was leveraged to retrieve additional articles.

The process of identifying relevant publications was driven by a domain-specific keyword

lexicon that was meticulously curated to encompass various terminological variations and application areas associated with GO. Search queries were formulated using Boolean logic operators (`AND`, `OR`, `NOT`) and proximity constraints to filter out false positives and capture documents from titles, abstracts, and author-defined keywords. The process is conceptually illustrated in Figure S.1, which showcases a typical query used to retrieve relevant literature from Web of Science, combining field-specific filters with structured logical conditions.

These queries were executed directly through the search interface of the website, the retrieval was automated using a Selenium-based Python script.[S5] This script efficiently navigated through the results pages and extracted the metadata of the retrieved articles, exporting the information directly into a structured Comma-Separated Values (CSV) file.

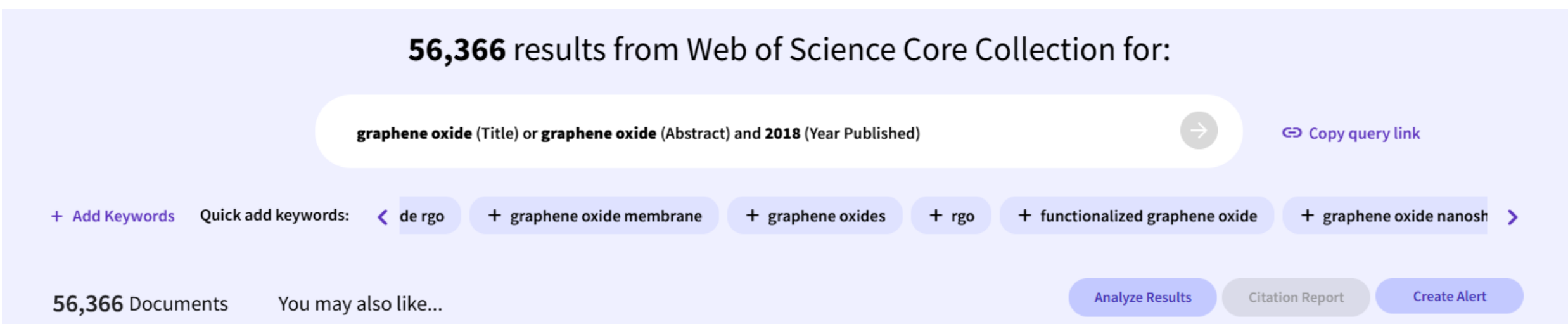

Figure S.1: Example query structure for the Web of Science, demonstrating the combination of keywords (e.g., "graphene oxide"), Boolean logic (`AND`, `OR`), and field specifiers (e.g., Title/Abstract/Keywords) used for indexing relevant publications.

A.2 Download

After indexing, the next critical task involved retrieving the full-text in PDF or XML formats of the identified publications. This task presented several challenges due to the variety of publisher platforms, inconsistent content structures, and access restrictions. To overcome these challenges, we developed a scalable automation strategy that employed a hybrid approach, combining API access with browser emulation techniques. Whenever possible, we utilized publisher APIs (such as those from Elsevier and Springer Nature)[S6,S7] to download documents in structured formats directly. Although these APIs offered stable and efficient

access, they often required authentication keys, rate-limit management, and compliance with content access restrictions.

For publishers lacking adequate APIs or requiring complex authentication procedures, a robust browser automation solution was implemented using Selenium WebDriver.[S5] This tool emulated human interaction in a headless browser (Google Chrome), allowing scripts to navigate login pages, resolve redirection chains, and interact with dynamic web content, including JavaScript-rendered download buttons.

Error-handling routines were implemented to capture and log instances of failed downloads, CAPTCHAs failures, or unexpected redirects, which triggered secondary workflows for retry or manual inspection. All retrieved PDFs were hashed and integrity was verified, while the associated metadata was cross-verified against the original indexing results to prevent duplication or corruption.

A.3 Processing the downloaded files

The downloaded text files were either XMLs files or PDFs. For XMLs, the processing is straightforward: we need to fetch the data written by the author and follow the text processing pipeline.

For PDF cases, there are two situations. Initially, the document is a native PDF that contains text and other elements, such as images. In such a case, we can extract the text directly. However, some documents contain less than 10% of pure text. Indeed, what appears to be text visually is actually an embedded image produced either by the publisher's pipeline or from "scanned images." From a processing viewpoint, such documents are a sequence of images. Therefore, we must extract the text from such "embedded images" using GOT-OCR 2.0[S8,S9] and, when applicable, Tesseract.[S10]

B Topic Modeling

The interconnectedness of the GO literature presents a complex analytical challenge. Identifying what is interconnected, how these connections occur, and when they emerge are foundational questions that inform the application of topic modeling. Clarifying these dimensions is essential before addressing the underlying reasons for such connections with precision and evidence.

Unsupervised topic modeling was used as the primary method to uncover emerging themes and latent structures within the GO literature corpus. The analytical pipeline combined Latent Dirichlet Allocation (LDA) for macro-level clustering with Neural Topic Modeling (BERTopic) applied within each topic identified by LDA.

B.1 Text preprocessing pipeline

Before LDA training, the text must be preprocessed. We build a single, lemmatized token corpus from XMLs and PDF-derived pure text (TXTs) and serialize one document per line (in JSONL format) for streaming into LDA. We extract the *entire* article text (titles, abstracts, body, captions, references) and normalize whitespace. Full-text ingestion typically increases tokens per document by $\sim$2–4$\times$ versus abstract-only pipelines; this broadens topical coverage but introduces boilerplate. Rather than exclude sections, we later control noise via filtering. We retain all documents that yield at least one token. Failed parses are counted and reported as a coverage rate (processed/discovered) $\approx$ 98%.

The tokenization uses NLTKs Punkt and `word_tokenize`,[S11] then lowercasing. We apply a strict *alphabetic-only* filter, which is our main vocabulary-size hyperparameter. This aggressively removes OCR debris, units, and mixed tokens, but it also excludes domain strings like "H2O," "GO-PEG," and "pH." In technical corpora, numerics and unit strings commonly account for a non-trivial fraction of raw tokens; therefore, this step can remove on the order of **20–40%** of tokens. If numerics or hyphens are essential, this hyperparameter can be relaxed to an alphanumeric/hyphen regex (e.g., `^[A-Za-z0-9-]+$`).

Stopwording is two-tiered. First, we remove the *standard NLTK English list* (≈180 terms, version-dependent). Second, we remove a *domain list of 40 tokens* (see Table S.1) to suppress generic reporting topics. The domain list was assembled via manual inspection of pilot models; this is ad hoc by design. We keep the list fixed across experiments and report separate removal shares for generic vs. domain stopwords. In practice, domain terms contribute an additional **5–15%** reduction of tokens beyond the generic list. URL-like residues starting with "http" are explicitly deleted.

To reduce sparsity without over-merging meanings, we perform *Part-Of-Speech (POS)-aware lemmatization*. The tokens are tagged with the averaged perceptron tagger of NLTK. The tags correspond to WordNets[S12] four POS classes (ADJ / VERB / NOUN / ADV), defaulting to NOUN due to ambiguity. This step typically shrinks the vocabulary by **10–25%** relative to the pre-lemmatized type count and improves the type–token ratio (TTR). We log pre- vs. post-lemmatization vocabulary sizes and the TTR shift. We did *not* include phrase modeling (bigrams/trigrams) in the core pipeline.

We process at an average of **50–100 documents per second**, depending on document length and the input/output data ratio on the server. To guarantee deterministic outputs across runs, we preload resources in each worker and centralize JSONL writing (one line = one document). Reproducibility hinges on fixed code, a fixed domain stopword list, and pinned NLTK versions; therefore, we emit version strings for `nltk`, `lxml`, and Python.[S13]

In short, the quantitative levers at preprocessing are: (i) token pattern (alphabetic-only vs. relaxed), which controls a **20–40%** discard band; (ii) stopword inventories (≈180 generic + 40 domain), which together remove another **30–50%** of remaining tokens; and (iii) POS-aware lemmatization, which contracts the vocabulary by **10–25%**. Choices outside these (full-text vs. abstracts, segmentation granularity, phrase modeling) were either fixed for stability or adjusted manually (ad hoc), and when changed, they were accompanied by reports of token retention, vocabulary size, and coherence/stability deltas.

Table S.1: Domain stopwords that are removed during the tokenization process.

```
et, al, figure, fig, table, also, data, using, study, result,
analysis, method, different, used, research, based, found, results,
conclusion, observed, indicate, significant, however, due, may,
well, within, can, could, should, might, would, new, one, two,
three, four, five, http, https
```

B.2 LDA Modeling

This study employs Latent Dirichlet Allocation (LDA), which represents documents as mixtures of latent topics, each topic characterized by a probability distribution over words.[S1] Formally, each document d is associated with topic proportions $\theta_d \sim \mathrm{Dir}(\alpha)$, and each topic k has a word distribution $\phi_k \sim \mathrm{Dir}(\eta)$. Here, α and η are hyperparameters that control the sparsity of the prior distributions. For each word w_{dn} in the document, a topic z_{dn} is first sampled from θ_d, followed by the word itself being sampled from $\phi_{z_{dn}}$

We perform phrase discovery, dictionary construction, token filtering, topic-number optimization by coherence, and final LDA training/evaluation over the preprocessed corpus. All operations are designed to stream data where possible, with explicit materialization only after Term Frequency – Inverse Document Frequency (TF–IDF) filtering.[S14]

The phrase modeling uses Gensim *Phrases*[S15] in two passes: a bigram phraser with `min_count=5` and `threshold=100`, followed by a trigram phraser trained on the bigrammed stream (same threshold; a lower `min_count` is commonly used in the second pass, but here

we rely on the threshold). Quantitatively, `min_count=5` suppresses idiosyncratic collocations that occur in < 5 documents, while `threshold=100` imposes a relatively strict scoring barrier. In practice, this yields a compact set of high-confidence phrases and typically increases the average document length in tokens by only a modest margin (phrases replace multiple unigrams). These values were chosen as conservative defaults from the pilot runs. Tuning them is ad hoc and is driven by observed coherence and qualitative topic interpretability.

We construct a dictionary directly from the transformed (bi/trigram) stream and then apply `dictionary.filter_extremes(no_below=5, no_above=0.5)`. The `no_below=5` cutoff removes terms that appear in fewer than five documents (reducing vocabulary noise and memory); `no_above=0.5` removes very common terms that occur in $> 50\%$ of documents (reducing background terms that degrade topic specificity). Together, these thresholds often shrink the type vocabulary by tens of percent relative to the raw phrase-augmented vocabulary. The precise effect is corpus-dependent and reported as the post-filter dictionary size in the logs.

Before modeling, we apply TF–IDF filtering to de-emphasize residual low-information terms. A TF–IDF model is fit on the Bag-of-Words corpus stream, and tokens with document-level TF–IDF weight < 0.03 are dropped (`tfidf_threshold=0.03`). This per-document gate typically removes an additional tail of common-but-not-globally frequent terms, yielding a filtered corpus and parallel filtered texts. The threshold is a single quantitative hyperparameter. We found 0.02–0.05 to be a reasonable band, with 0.03 adopted here. This choice is empirical (manual tuning) and trades topic sharpness for vocabulary coverage. Overly aggressive thresholds can collapse documents or bias topics toward rare terms.

To select the number of topics, we sweep `num_topics` from 2 to 50 in steps of 2, train an `LdaMulticore`[S15] model for each value and compute `c_v` coherence[S16] on the filtered texts. For training stability, we fix `PASSES=50` with a fixed seed (`random_state`), and use all available CPU cores (`workers`). We set `CHUNKSIZE=1000` to balance throughput and memory. Within this grid, the optimal topic count is the maximum argument of coherence. Practically,

the grid size is $(50-2)/2 = 24$ models; with `passes=50`, this is computationally expensive but yields smooth coherence curves. If memory pressure occurs, reducing `workers` or `passes` is the primary lever; chunking primarily affects throughput. All other LDA defaults (Dirichlet priors, decay) remain at Gensim defaults; when not explicitly tuned, they are de facto ad hoc defaults.

After identifying the optimal topic count, we retrain a final `LdaMulticore` model on the filtered corpus using the selected `num_topics` and the same `passes`, `chunksize`, and `random_state`. We report `c_v` coherence. Coherence is our model-selection metric, given its strong correlation with human interpretability for scientific text. We export top-10 word lists per topic as discussed below.

FiguresS.2–S.9 highlight the vocabulary most characteristic of each LDA topic, as identified by the final model trained on the fully preprocessed corpus. For each topic, we display the top N tokens ranked by the learned probability $P(w|k)$ where w is a word from the vocabulary and k is the topic number, indicating the strength of association between a term and its topic in the context of the entire corpus. Since only the most prominent terms are shown, the bars should be interpreted collectively; together, they should form a coherent scientific theme, such as an application area, material system, or method. Some overlap in general scientific vocabulary across topics is expected. If a topic is dominated by reporting language, consider reviewing the extreme-term pruning and domain-specific stopword list. The same value of N is used in all figures to ensure comparability.

The topic maps provide a coherent overview of key terms across different application areas. In *Catalysis* (Figure S.2), frequently occurring words include transition metals (Ni, Co, Fe, Cu), grapheneoxide supports (GO/rGO), and reactions such as activity, degradation, and oxidation. This pattern suggests two main lines of work: 1) electrocatalysis, which focuses on reactions such as oxygen reduction/evolution, hydrogen evolution, alcohol and urea oxidation, and applications in direct methanol or direct borohydride fuel cells (DMFC/DBFC), typically involving doped or carbon-supported metals; and 2) photocatalysis or advanced

oxidation processes aimed at degrading dyes and other organic contaminants, often utilizing heterojunction structures under visible light. The repeated references to composites, nanoparticles, and supports underscore the importance of material hybridization for optimizing performance.

For *Energy Storage* (Figure S.3), the most salient terms include device-related words (battery, supercapacitor, electrode, cycle, capacity, rate) alongside materials (MXene, graphene, polymers, electrolytes, separators). Safety and interface engineering come through in mentions of dendrites, stability, and solid or gel electrolytes, while references to ions, vanadium, and deionization link to vanadium redox flow batteries (VRFB) and capacitive deionization (CDI). Overall, this is a mature, engineering-driven space that spans electrodes, electrolytes, and integrated device design.

In the area of *Polymer & Composite Reinforcement* (Figure S.4), the most prominent terms relate to mechanics and transport, including composites, polymers, matrices, reinforcement, strength, and conductivity. The presence of words such as asphalt, cement, and concrete indicates applications in civil engineering, while tribology, wear, and friction emphasize surface durability. References to percolation, modeling, and simulation highlight the importance of structureproperty relationships in electrical and thermal networks, as well as in plate and beam mechanics. For *Soft Matter and Scaffolds* (Figure S.5), the vocabulary is dominated by terms such as hydrogel, aerogel, scaffold, and porous, often accompanied by crosslinking, biocompatibility, and regeneration, all of which point to the development of soft, biofunctional materials for tissue engineering. Related terms like fiber, ink, printed, and flexible are associated with advances in printed and flexible electronics. At the same time, membrane, wettability, and separation underscore innovations in interface-engineered films and selective transport.

In the *Biomedical & Drug Delivery* topic (Figure S.6), the most prominent terms include drug, delivery, therapy, cancer, and cell, alongside references to carriers and probes such as nanoparticles, functionalization, targeting, and imaging. Considerations of safety

and biological interactions are reflected in terms like toxicity, biocompatibility, and interaction, while device- and immune-related applications are captured by antibacterial, coatings, and vaccine/adjuvant. Collectively, these terms outline a progression from carrier design through to in vitro and in vivo studies. In *Water Treatment & Membranes* (Figure S.7), the vocabulary is distinctly separated by treatment method and contaminant type. Adsorption-related terms (adsorption, isotherm, capacity, sites, Langmuir) appear together with catalytic and photocatalytic descriptors (photocatalysis, degradation, visible) and membrane-specific language (membrane, flux, selectivity). The presence of pollutant markers (dyes, metals, arsenic, selenium, PFAS) and material indicators (porous, mixed-matrix, polyvinylidene fluoride (PVDF), MXene) highlights the intentional design of materials to target specific classes of contaminants.

In *Nanosensors* (Figure S.8), key terms such as sensor, electrochemical, signal, limit, and detection appear alongside biorecognition elements like aptamers, immunoassays, and enzymes, as well as various transduction methods including SERS (Surface-Enhanced Raman Spectroscopy), fluorescence, and ECL (Electrochemiluminescence). Common analytes, including glucose, dopamine, metal ions, and antibiotics, are frequently referenced, while terms like wearable, printed, and microfluidic reflect a range of deployment formats. Together, these terms illustrate a framework based on recognition strategy, transduction technique, and intended application. Finally, for *Films and Coatings* (Figure S.9), the vocabulary divides into protective and functional themes. Terms such as coating, film, corrosion, and barrier are associated with protective applications, while transparent, electrode, EMI (Electromagnetic Interference), and conductivity relate to electronic or multifunctional films. Additional references to reduction, annealing, layers, and composites highlight the importance of fabrication processes in determining performance. Terms like wettability, hydrophobicity, and anti-fouling underscore the role of surface engineering in specific operational environments.

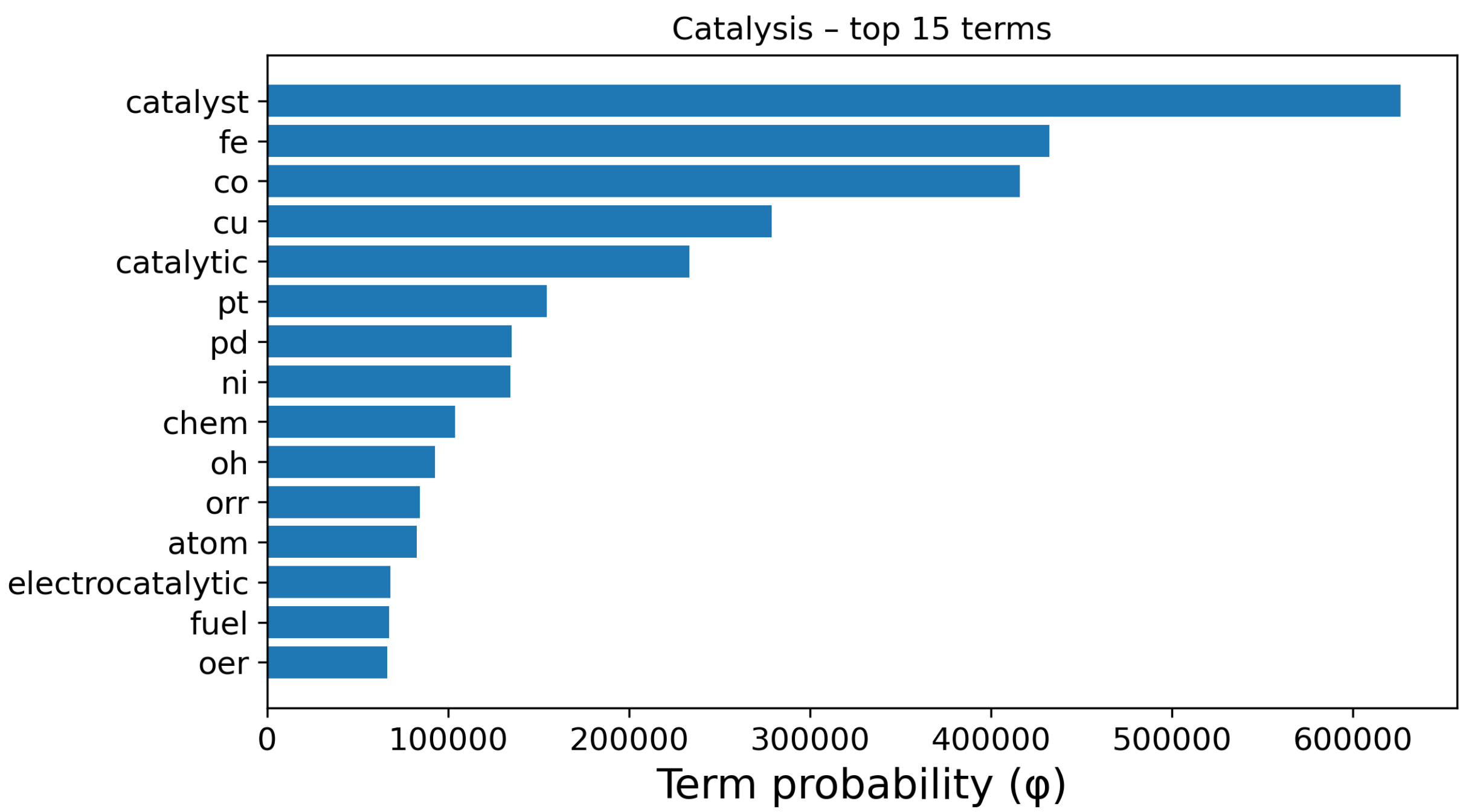

Figure S.2: Top 15 terms of the Catalysis macro-topic.

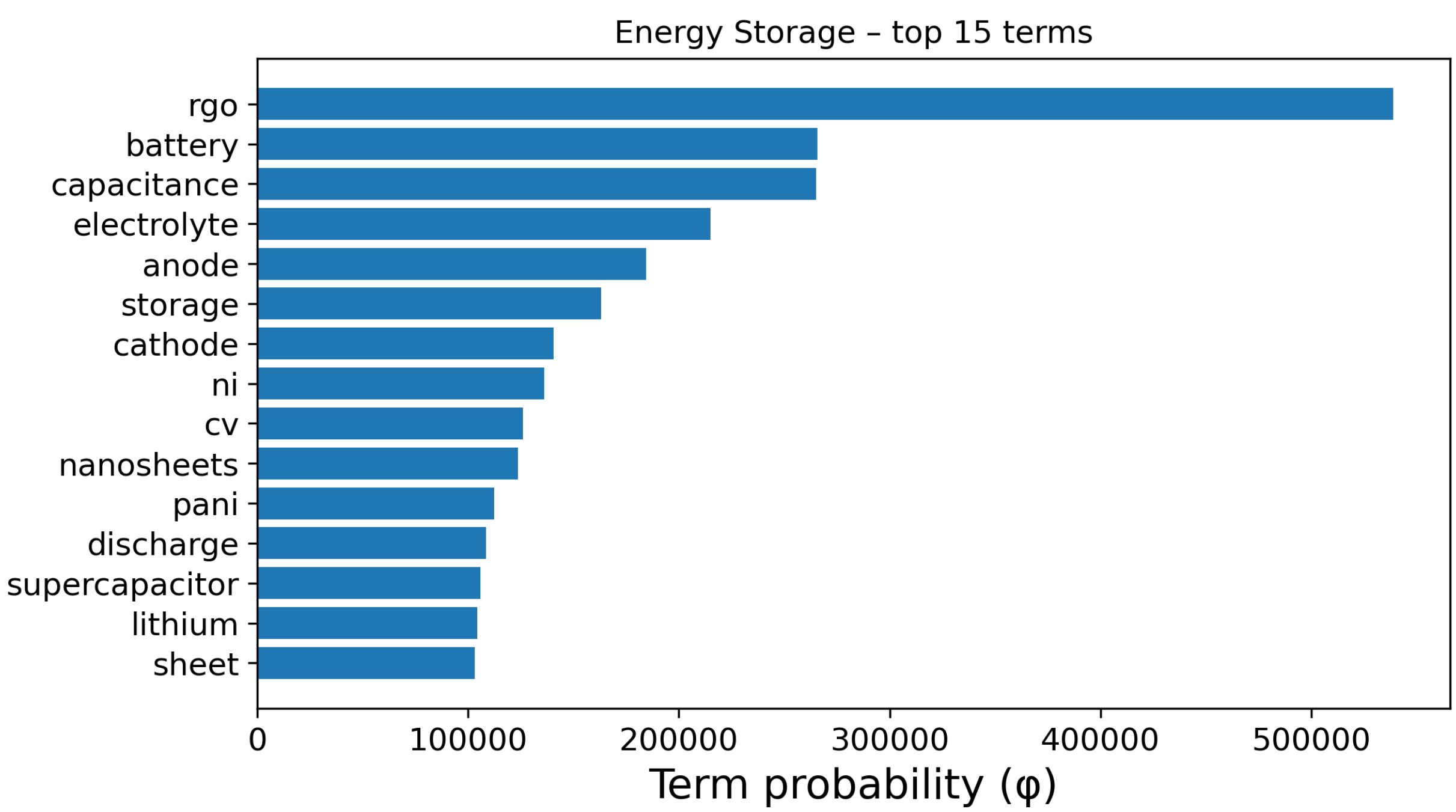

Figure S.3: Top 15 terms of the Energy Storage macro-topic.

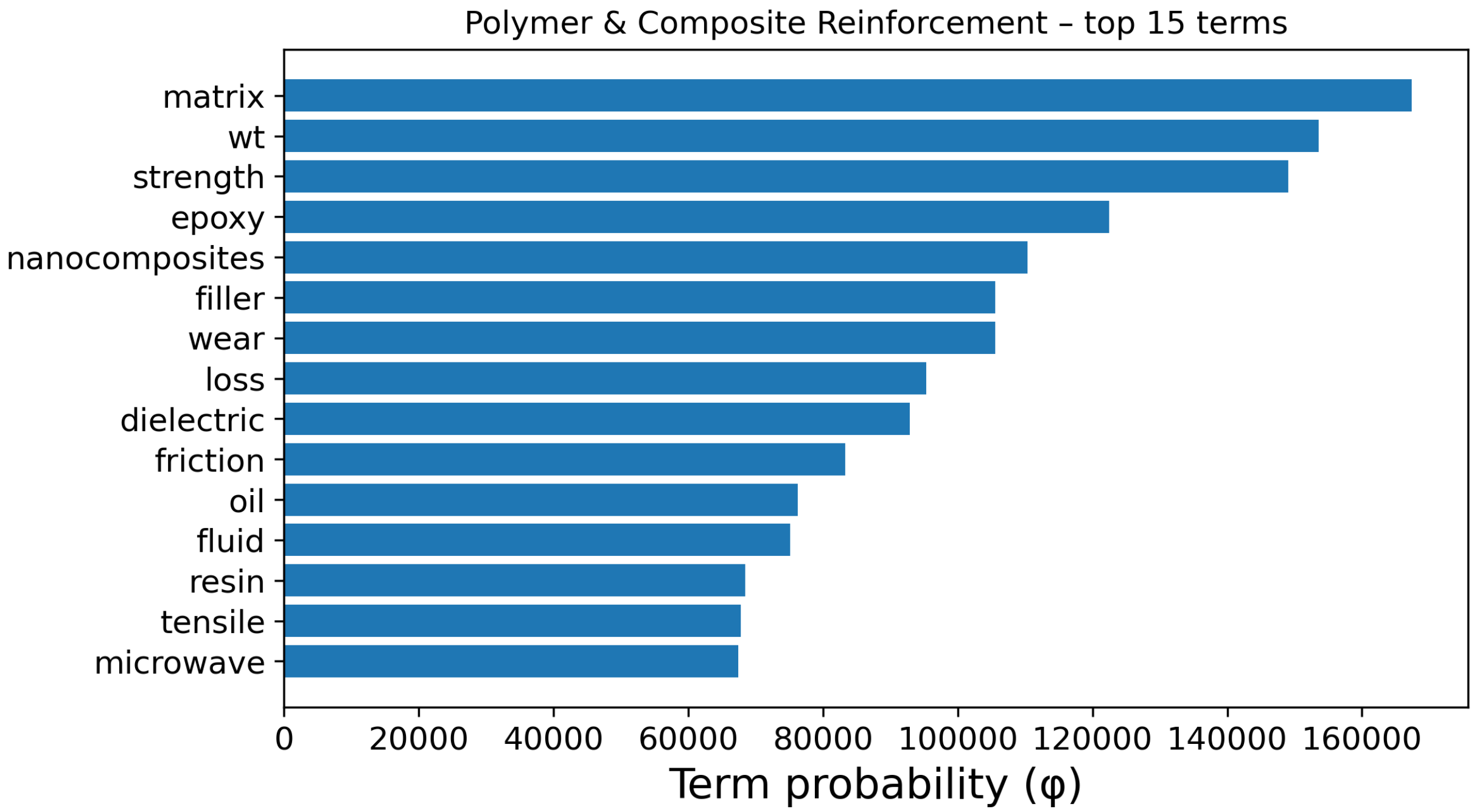

Figure S.4: Top 15 terms of the Polymer and Composite Reinforcement macro-topic.

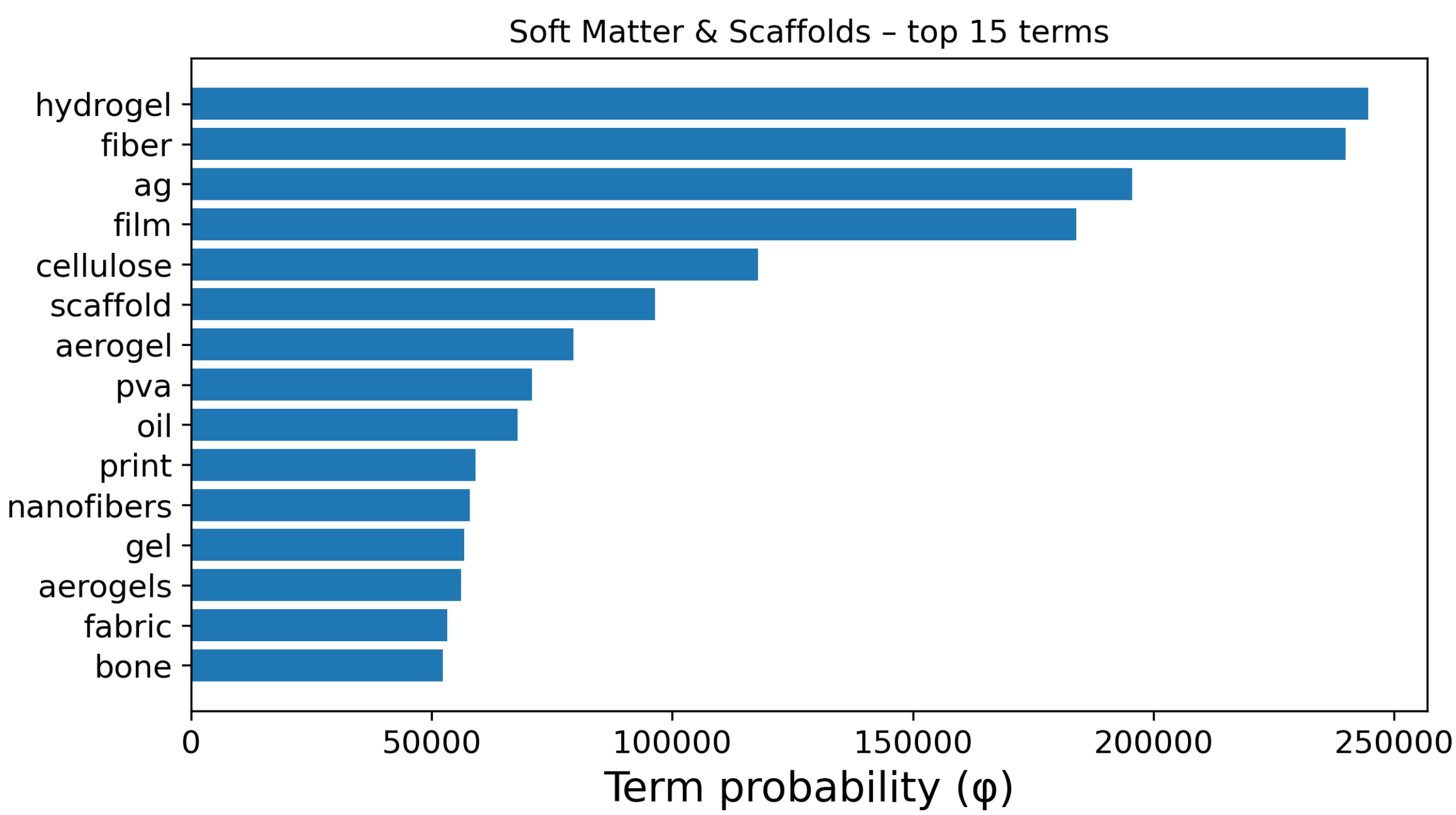

Figure S.5: Top 15 terms of the Soft Matter and Scaffolds macro-topic.

B.3 Neural Topic Modeling

We refine the LDA-discovered structure with a second-stage, embedding-based topic model (Neural Topic Modeling)[S2] applied *within* each LDA topic. The pipeline loads a preprocessed

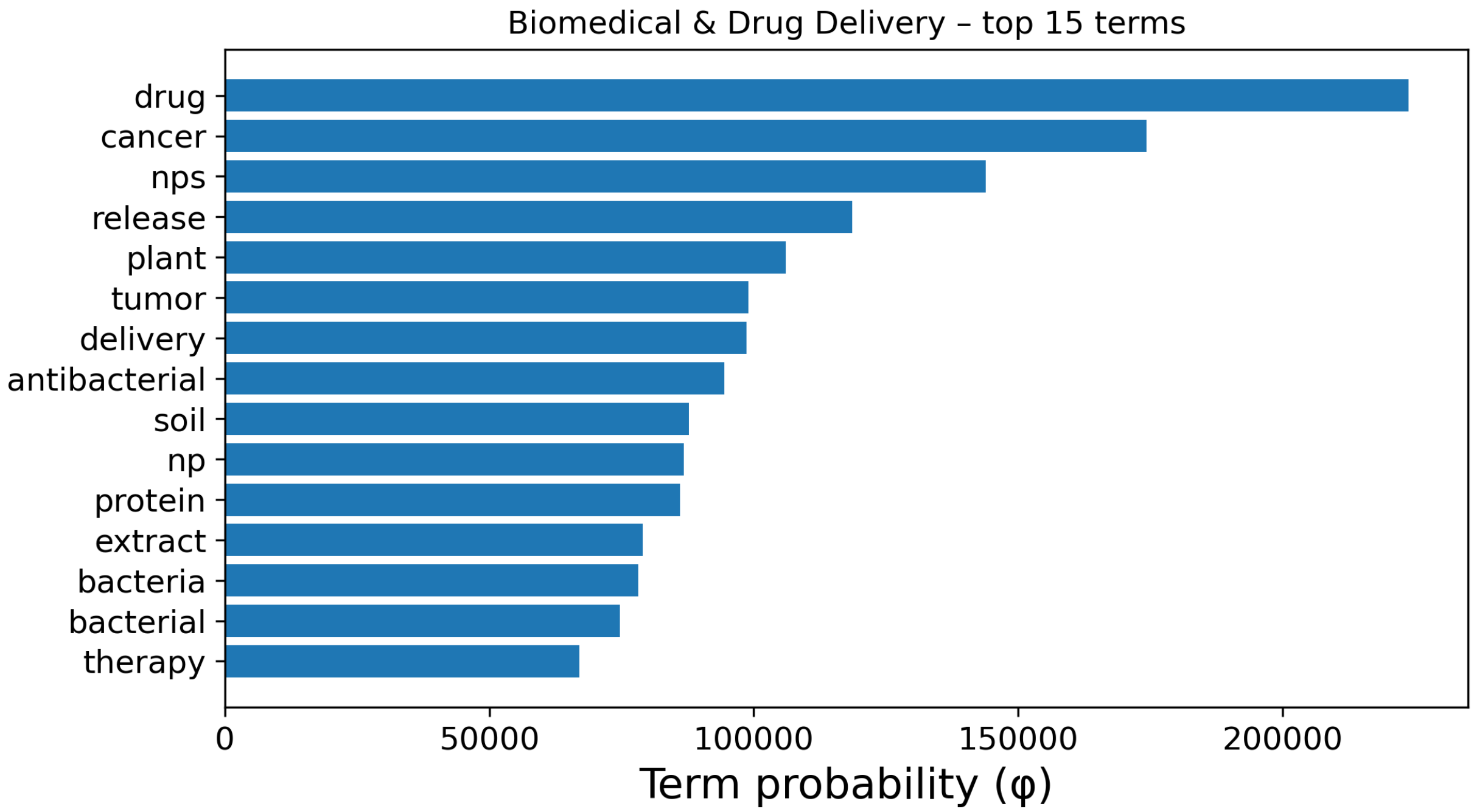

Figure S.6: Top 15 terms of the Biomedical and Drug Delivery macro-topic.

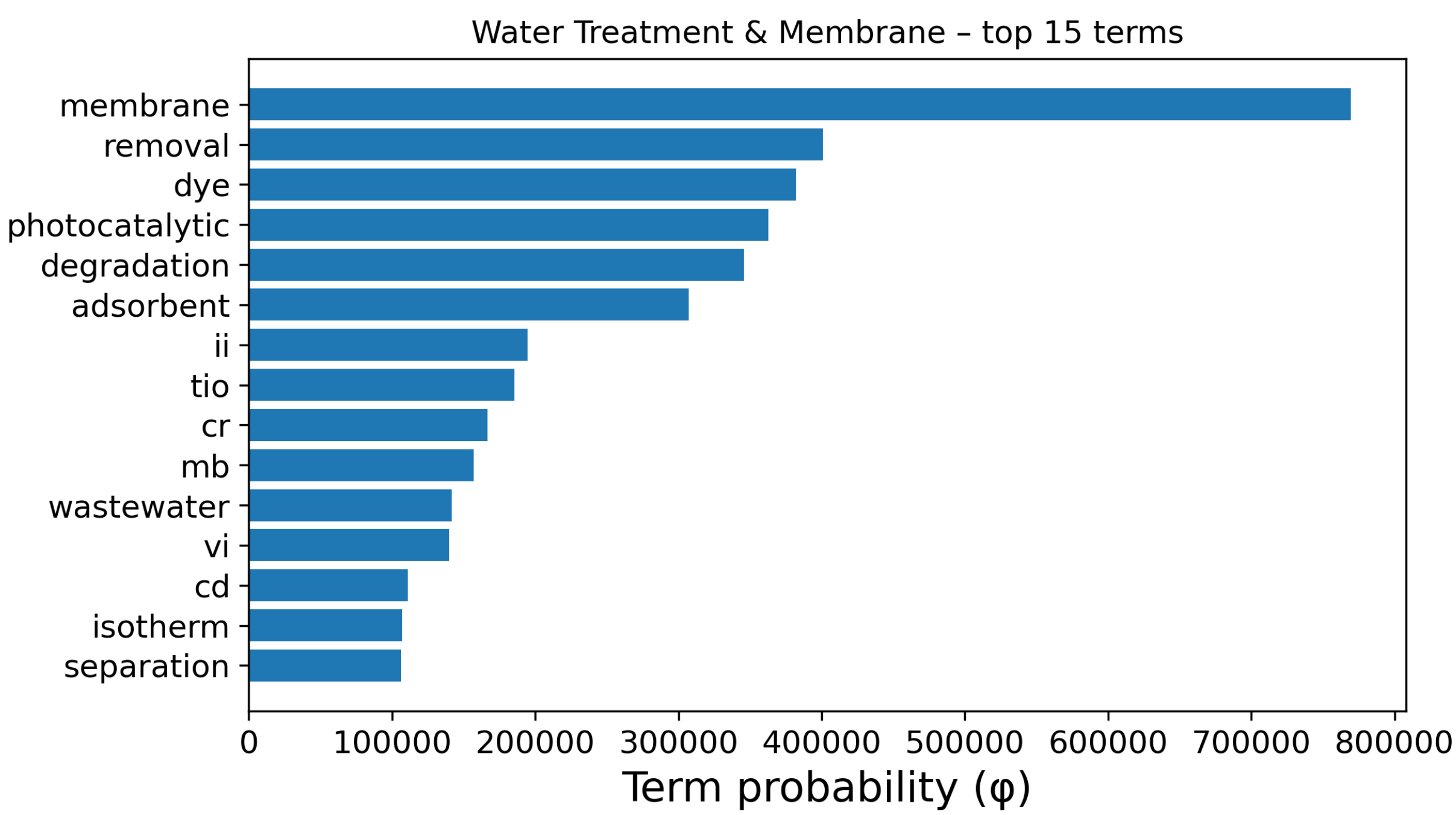

Figure S.7: Top 15 terms of the Water Treatment and Membrane macro-topic.

corpus and a trained LDA model, reconstructs the same phrase and dictionary settings used

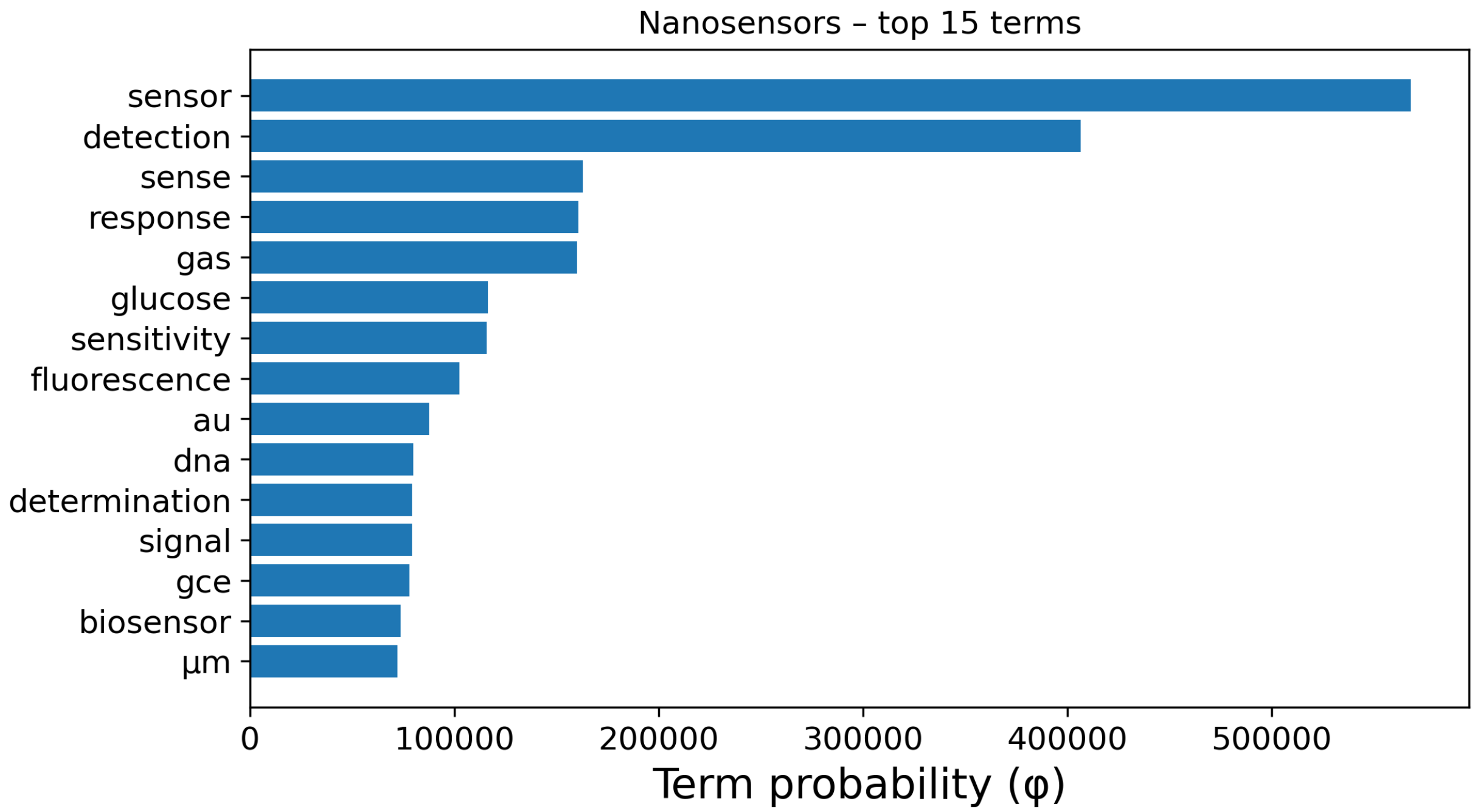

Figure S.8: Top 15 terms of the Nanosensors macro-topic.

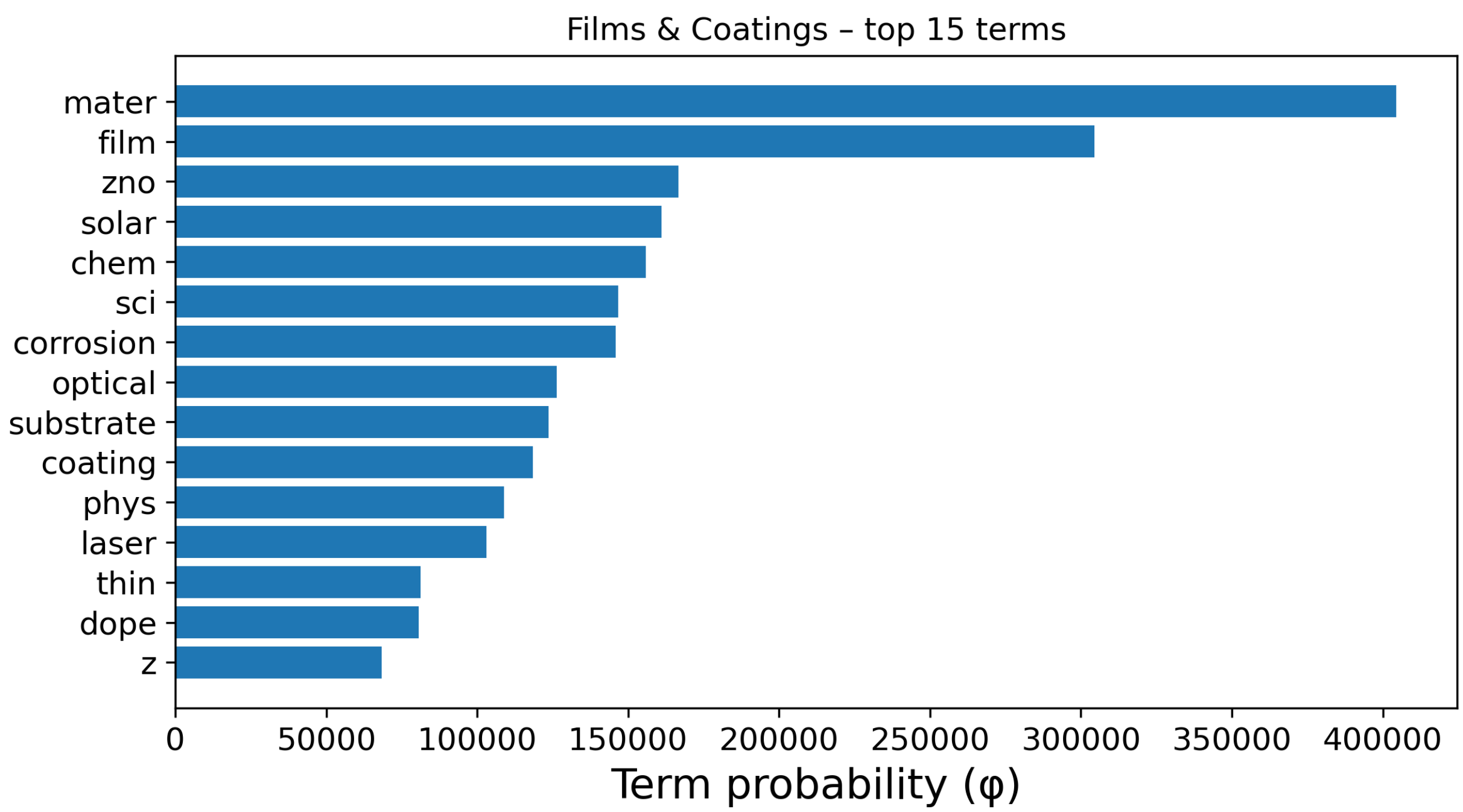

Figure S.9: Top 15 terms of the Films and Coatings macro-topic.

during LDA, filters tokens using TF–IDF, assigns each document to its dominant LDA topic, and then runs Neural Topic Modeling on the grouped documents.

To preserve compatibility with the LDA stage, we reapply phrase discovery with Gensim `Phrases`[S15] using `min_count=5` and `threshold=100` for both bigrams and trigrams (the same conservative barrier as the LDA pipeline). These values suppress collocations that are rare ($<$ 5 documents) or weakly associated (score $<$ 100), yielding a compact phrase vocabulary and stable downstream clusters. Their selection was ad hoc, guided by coherence and qualitative interpretability from the pilots. The dictionary is then rebuilt from the phrase tokens and pruned with `filter_extremes(no_below=5, no_above=0.5)`, removing terms that appear in fewer than five documents or in more than 50% of documents. In practice, these cutoffs reduce the type vocabulary by tens of percent while preserving task-relevant terms.

Before LDA assignment, we compute TF–IDF[S14] weights on the Bag-of-Words corpus and drop tokens with per-document TF–IDF < 0.03. This single quantitative gate typically removes a thin tail of low-information tokens that survive frequency pruning. A band of 0.02–0.05 worked reasonably well in pilots, and we adopt 0.03 for further experiments (empirical, ad hoc). Memory is managed explicitly to keep the footprint bounded as intermediate lists are discarded.

Document-to-topic assignment uses a `LdaMulticore` model: for each filtered document, we compute the topic distribution and take the maximum argument as the dominant LDA topic. We then concatenate the phrased tokens into a single string per document and group the texts by LDA topic identifier. The script reports group sizes (documents per LDA topic) and skips Neural Topic Modeling on undersized groups using the parameter `MIN_DOCS_FOR_BERTOPIC=10`.

Furthermore, Neural Topic modeling starts from a sentence-level encoder $f_{\text{enc}}(\cdot)$ that maps a document d (or section/paragraph) to an embedding $x_d \in \mathbb{R}^p$ where p is a fixed number of dimensions.[S17] Next, the dimensionality of these embeddings is reduced using Uniform Manifold Approximation and Projection (UMAP) $g : \mathbb{R}^p \to \mathbb{R}^q$, for $q < p$, to obtain $y_d = g(x_d)$.[S18] The projected points are then clustered using HDBSCAN $h(\cdot)$, an

algorithm that produces cluster labels $c_d = h(y_d)$ that are robust to noise and varying cluster densities.[S19] For each cluster c, Neural Topic Modeling (following BERTopic's approach) constructs a sparse topic representation using class-based Term Frequency-Inverse Document Frequency (TF-IDF, c-TF-IDF).[S2] If $f_{t,c}$ denotes the frequency of term t in the concatenated documents assigned to cluster c, $|d_c|$ the total token count of that concatenated "class document," N the number of classes, and n_t the number of classes in which term t appears, for embedding-based refinement, we instantiate Neural Topic Modeling with Qwen3-8B-Embedding[S17] to better capture domain semantics, and a UMAP reducer configured as `UMAP(n_neighbors=15, n_components=100, min_dist=0.0, metric=cosine)` with fixed seed.[S18] These hyperparameters emphasize local neighborhood fidelity (`n_neighbors=15`) while allowing dense low-dimensional representations (`n_components=100`) and tight cluster formation (`min_dist=0.0`) under a cosine metric. Neural Topic Modeling is run with `nr_topics="auto"` to merge clusters based on internal criteria until a coherence-informed optimum is reached. This automation is intentionally ad hoc and offloads fine-grained cluster-count tuning to the library.

Quantitatively, the key parameter values used are: phrase discovery (`min_count=5`, `threshold=100`), dictionary pruning (`no_below=5`, `no_above=0.5`), TF–IDF gating (`0.03`), the Neural Topic Modeling minimum-sample guard (`MIN_DOCS_FOR_BERTOPIC=10`), UMAP settings (`n_neighbors=15`, `n_components=50`, `min_dist=0.0`, `metric= cosine`). Decisions not explicitly tuned (e.g., HDBSCAN parameters inherited via defaults,[S19] the `nr_topics="auto"` merge schedule) should be regarded as pragmatic defaults. The pipeline logs end-to-end and per-step wall-clock times, the number of documents entering and exiting each stage, and per-topic document counts, enabling sensitivity analyzes (e.g., raising/lowering `TFIDF_THRESHOLD` or `MIN_DOCS_FOR_BERTOPIC`) without altering code semantics.

B.4 LLM-Assisted Interpretation

To automatically assess the semantic coherence of each topic and subtopic, we employed a large language model to generate descriptive summaries capturing the common theme underlying the top-ranked words of each topic. This approach allowed us to infer the latent meaning associated with word clusters beyond simple keyword inspection. The model was prompted with the list of top words for each topic and instructed to articulate the most plausible scientific theme or conceptual domain they represented. All generated descriptions were subsequently reviewed and validated by the research team to ensure alignment with domain knowledge and consistency across the corpus.

B.5 Hierarchical visualization of subtopics

To examine the internal structure of each LDA macro-topic, we trained a dedicated BERTopic-style model on the documents assigned to that macro-topic, using Qwen3-Embedding-8B embeddings, UMAP for dimensionality reduction, and HDBSCAN for clustering.[S17–S19] For each domain, the upper panel provides a semantic map of the resulting subtopic neighborhoods, whereas the lower panel shows a complete-linkage dendrogram built from the corresponding subtopic representations in embedding space. Together, these two views show how fine-grained research niches are arranged locally and how they aggregate into broader conceptual families. Because UMAP is primarily useful for qualitative neighborhood structure, and because dendrogram heights are interpretable within a given figure but should not be compared directly across different macro-topics, cross-domain comparisons are based on branching topology and cluster balance rather than on absolute two-dimensional positions or absolute linkage heights.

The *Catalysis* domain (Figure S.10) is organized around a relatively compact set of mechanistic families. The semantic map highlights subtopics associated with oxygen-reduction electrocatalysts, direct-ethanol-fuel-cell catalysts, bifunctional transition-metal systems for water splitting, ammonia–borane hydrolysis for hydrogen generation, heterogeneous Fen-

ton and electro-Fenton catalysis for wastewater cleanup, nitrophenol reduction, enzymatic biodiesel synthesis, and ammonium-perchlorate decomposition. In the dendrogram, these niches consolidate into a small number of higher-level branches, indicating that catalytic GO research is structured mainly by reaction class. One broad branch is dominated by electrochemical catalysis and fuel-related reactions, another captures heterogeneous and green catalytic routes, and a third gathers oxidation- and cleanup-oriented catalysis. Thus, although specialized subfields exist, the domain remains comparatively consolidated around a few dominant catalytic narratives.

The *Energy Storage & Conversion* domain (Figure S.11) is broader and more heterogeneous, but it is still held together by a strong electrochemical backbone. The map contains clear neighborhoods corresponding to MnO_2-carbon supercapacitor electrodes, Li-ion anodes based on SnO_2–graphene, lithium–sulfur batteries, Pt-free carbon counter-electrodes for dye-sensitized solar cells, MoS_2-based hydrogen-evolution electrocatalysts, capacitive deionization, graphene-reinforced anticorrosion coatings, and graphene-oxide reduction or processing routes. The dendrogram shows that these niches do not form a single uninterrupted continuum; instead, they aggregate into several substantial branches associated with supercapacitors, battery electrodes, electrochemical energy-conversion devices, and adjacent functional materials. This pattern suggests an intermediate level of internal diversification: the domain is not dominated by only one device class, but neither is it dispersed into many unrelated micro-communities.

The *Polymer & Composite Reinforcement* domain (Figure S.12) is organized primarily by host matrix and targeted performance function. The semantic map identifies subtopics related to microwave-absorbing composites and coatings, PLA crystallization and toughening, halogen-free flame-retardant epoxy and polyolefin systems, fly-ash geopolymer composites, aluminum-matrix composites, polymer tribology, phase-change thermal-storage materials, hybrid nanofluid heat transfer, and biodiesel-engine performance. The dendrogram shows that these niches cluster into several recognizable higher-level groupings, including mul-

tifunctional polymer composites, epoxy and flame-engineering systems, metal-matrix and tribological materials, and heat-transfer or process-fluid applications. The resulting hierarchy is neither extremely compact nor highly dispersed. Instead, it reflects an intermediate regime in which a limited number of large material families support substantial internal specialization.

The *Soft Matter & Scaffolds* domain (Figure S.13) is organized mainly by matrix chemistry and material form. The map reveals neighborhoods corresponding to bone-tissue-engineering scaffolds, hydrogel-based adsorbents for dye and heavy-metal removal, self-healing conductive hydrogels for wearable motion sensing, PLA-based biocomposites for sustainable packaging, fiber-surface modification for interfacial adhesion, enzyme immobilization on magnetic nanoparticles, and solar-driven desalination or photothermal evaporation. In the dendrogram, these niches group into a few higher-level families that distinguish regenerative scaffolds and therapeutic hydrogels from cleanup-oriented aerogels and hydrogels, conductive fibers and printed electronics, and wearable smart-textile systems. A notable feature of this domain is that biomedical and environmental soft materials remain partially connected through shared language about hydrated, porous, or flexible matrices. The overall structure is therefore moderately differentiated, with clear major branches but substantial conceptual continuity across applications.

The *Biomedical & Drug Delivery* domain (Figure S.14) displays a more distributed internal organization. The map contains distinct neighborhoods for stimuli-responsive nanocarriers for targeted anticancer delivery, near-infrared-triggered chemo-photothermal therapy, neurodegenerative-disease therapeutics and brain delivery, antimicrobial hydrogels and phototherapies, and electrochemical sensing of drugs or H_2O_2. At the same time, the figure also shows adjacent offshoots linked to dye adsorption, heavy-metal soil remediation, and green ZnO nanomaterials, indicating that this macro-topic borders remediation and ecotoxicological themes. The dendrogram reflects this mixture by separating targeted nanomedicine, regenerative or antimicrobial biomaterials, diagnostic and monitoring platforms, and envi-

ronmentally oriented subbranches into multiple comparable groups. Rather than forming one smooth nanomedicine continuum, biomedical GO research divides into several workflow-centered branches defined by delivery strategy, therapeutic mechanism, sensing function, or bioenvironmental context.

The *Water Treatment & Membrane* domain (Figure S.15) is one of the most clearly mechanism-structured domains. The map shows prominent neighborhoods associated with heavy-metal adsorption, antibiotic and dye removal, arsenic and selenium remediation, uranium extraction with functionalized sorbents, enzymatic or electrochemical decolorization of azo-dye wastewater, ZnO-based photocatalytic dye degradation, photocatalytic self-cleaning membranes for micropollutant removal, CO_2/CH_4 separation in mixed-matrix membranes, and a smaller photocatalytic hydrogen-production niche. In the dendrogram, these subtopics collapse into a few dominant higher-level branches corresponding to adsorption-driven removal, visible-light or advanced-oxidation photocatalysis, and membrane-based separations. Pollutant identity is clearly secondary to treatment mechanism as an organizing principle. The resulting hierarchy is comparatively consolidated, with a small number of dominant mechanistic families and only a few more specialized offshoots.

The *Nanosensors* domain (Figure S.16) is among the most internally differentiated. The map reveals separate neighborhoods for non-enzymatic glucose sensors, non-enzymatic hydrogen-peroxide sensors, electrochemical sensing of toxic phenolics, pesticide-residue detection, simultaneous electrochemical detection panels, ultrasensitive miRNA assays, SERS substrates for food safety, mycotoxin aptasensors, advanced humidity sensors based on QCM, SAW, or flexible technologies, and nanocomposite coatings for sample preparation and extraction. The dendrogram confirms that these niches remain distributed across several mid-sized branches rather than collapsing into only a few dominant ones. These branches track both analyte class and transduction mode, separating electrochemical biosensing, nucleic-acid or aptamer sensing, food-safety detection, contaminant sensing, and materials-assisted extraction workflows. In this case, platform physics and sensing protocol organize the hier-

archy more strongly than the generic label of "sensor," yielding a comparatively fragmented internal structure.

The *Films & Coatings* domain (Figure S.17) combines functional thin-film devices with surface-engineered coatings and transport interfaces. The map contains labeled neighborhoods for advanced anticorrosive epoxy coatings, membrane nanochannels for desalination and ionic separation, CVD graphene growth on Cu and dielectric substrates, skin-mountable pressure and strain sensors, Schottky-diode interface engineering, passively mode-locked fiber lasers, memristive neuromorphic devices, single-atom electrocatalysts, and emergent quantum phases in kagome or moiré heterostructures. The dendrogram organizes these niches into a small number of broad super-branches. One side is dominated by device-physics and electronic-interface themes, including neuromorphic, optoelectronic, and quantum-material directions; the other emphasizes coatings, interfacial transport, graphene growth, and multifunctional surface technologies. This domain is therefore best described as moderately heterogeneous: it contains several clearly distinct technological threads, but these still aggregate into a limited number of higher-level families.

Together, these figures show that GO research does not subdivide uniformly across applications. Some macro-topics, especially *Catalysis* and *Water Treatment & Membrane*, are comparatively consolidated and are dominated by a few mechanistic branches. Other applications, especially *Nanosensors* and *Biomedical & Drug Delivery*, are more distributed internally and contain several comparable branches associated with different targets, device modalities or protocol families. The remaining domains occupy an intermediate regime in which a limited number of high-level divisions coexist with substantial specialization within each branch. The semantic maps therefore provide a qualitative geography of subtopic neighborhoods, whereas the dendrograms make explicit how those neighborhoods aggregate into broader conceptual families, reinforcing the conclusion that each GO application domain exhibits its own characteristic internal hierarchy.

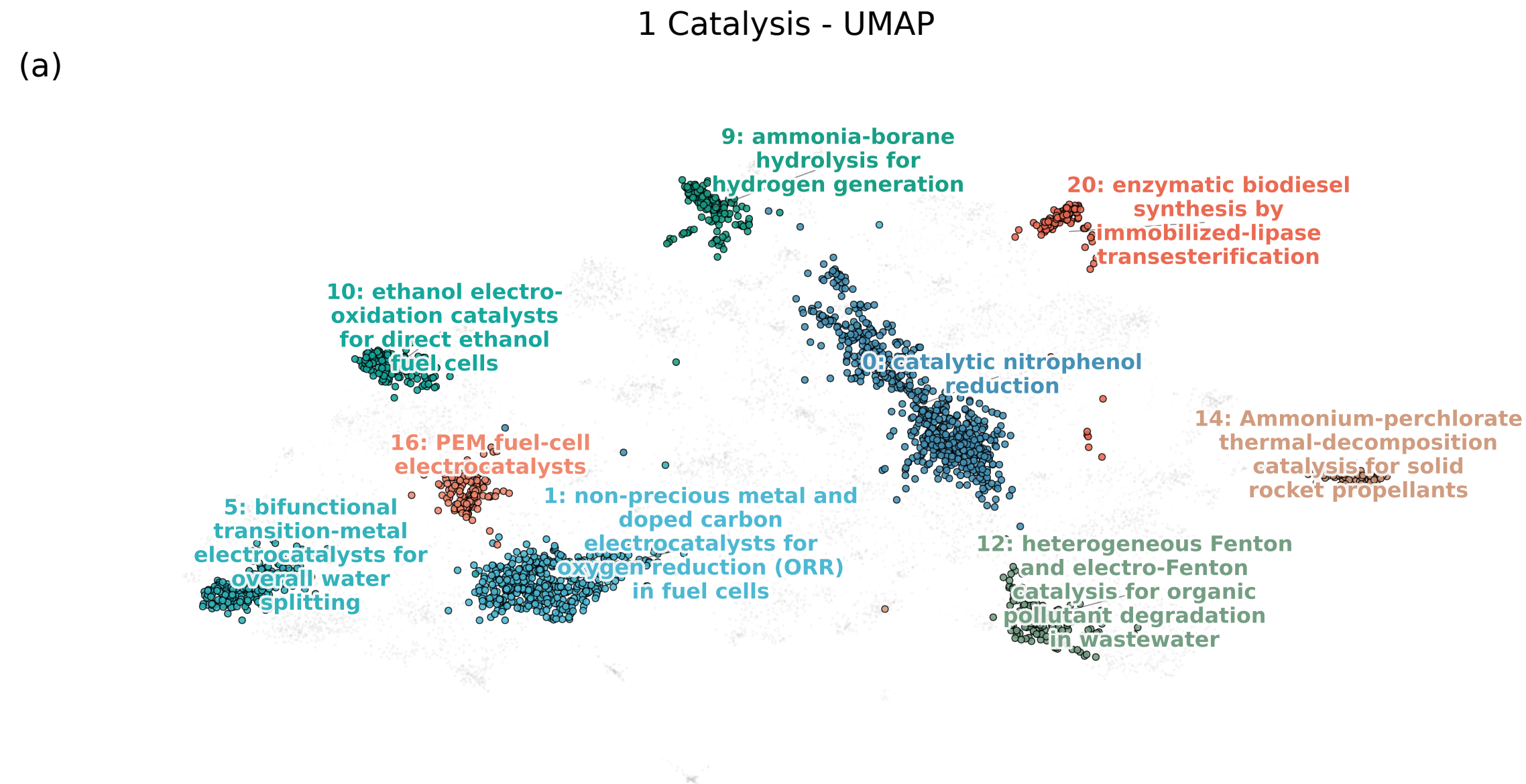

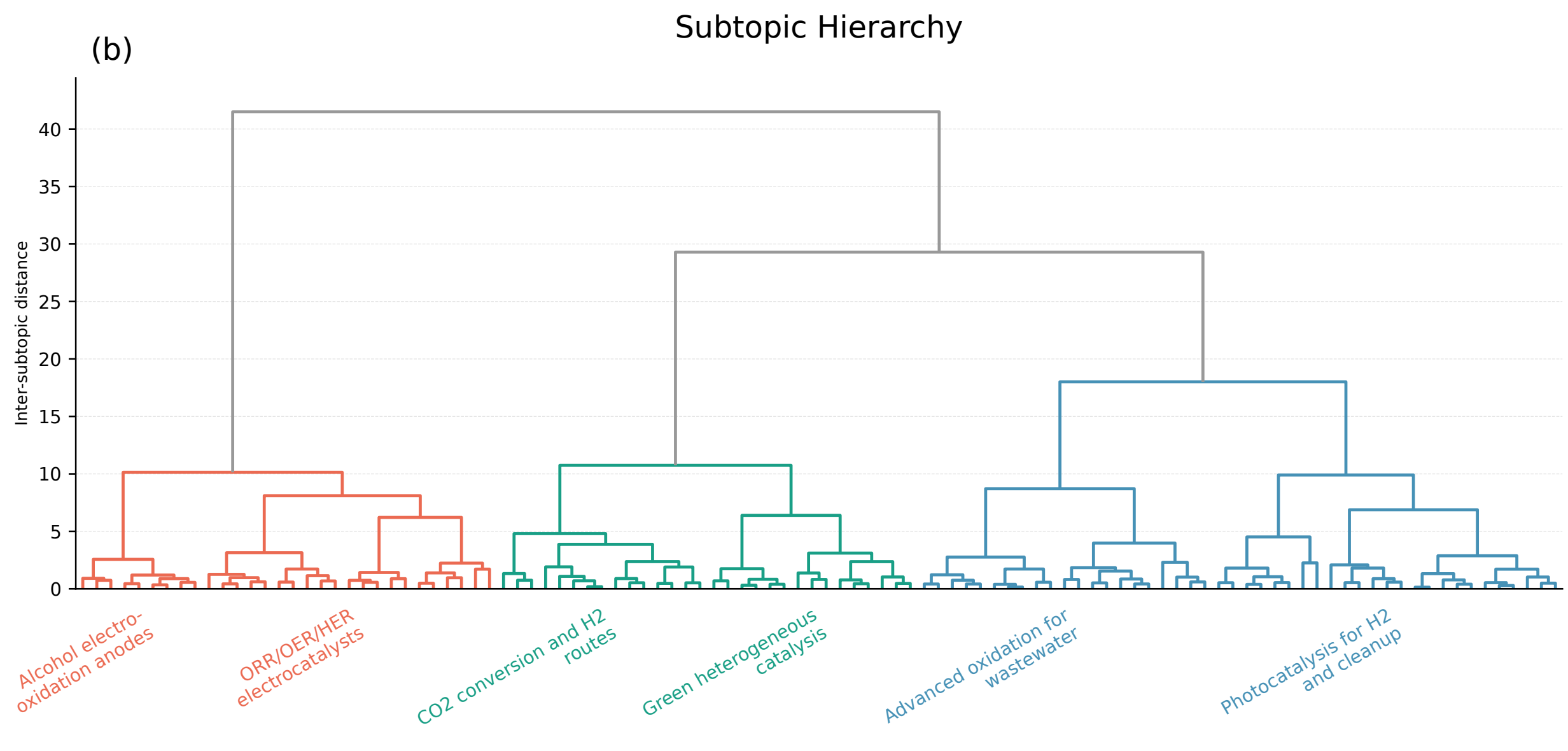

Figure S.10: a) Intertopic distance map for Catalysis. b) Dendrogram of hierarchical clustering for Catalysis.

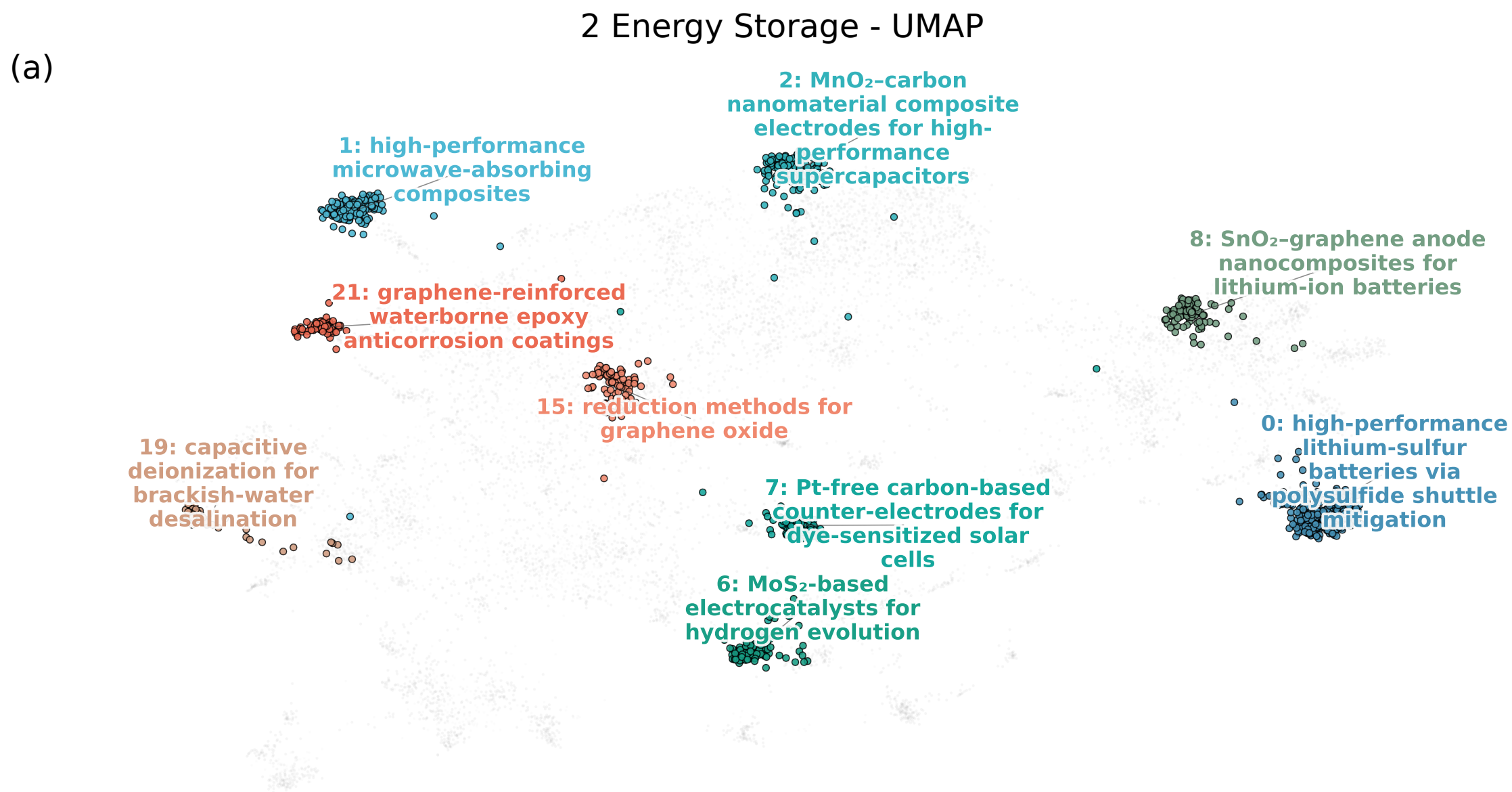

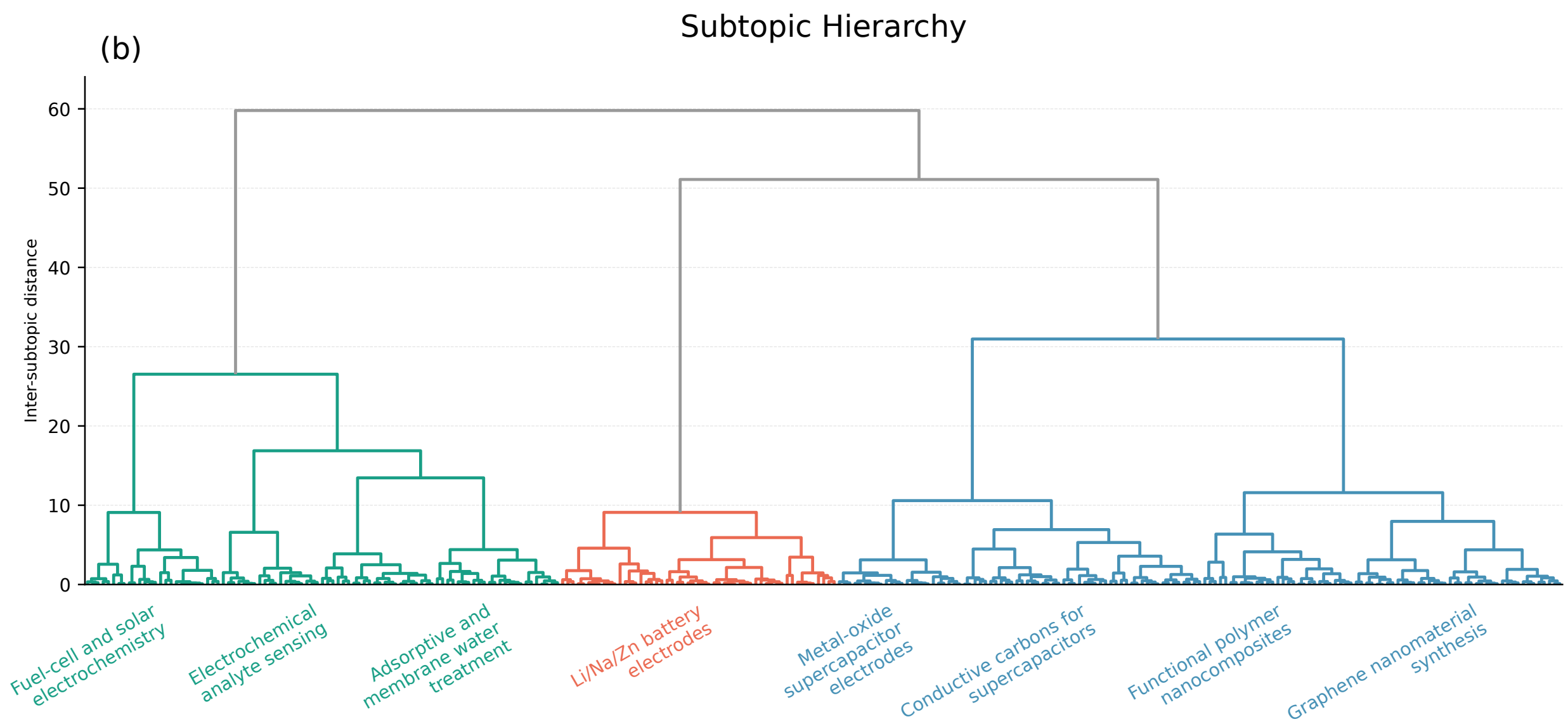

Figure S.11: a) Intertopic distance map for Energy Storage. b) Dendrogram of hierarchical clustering for Energy Storage.

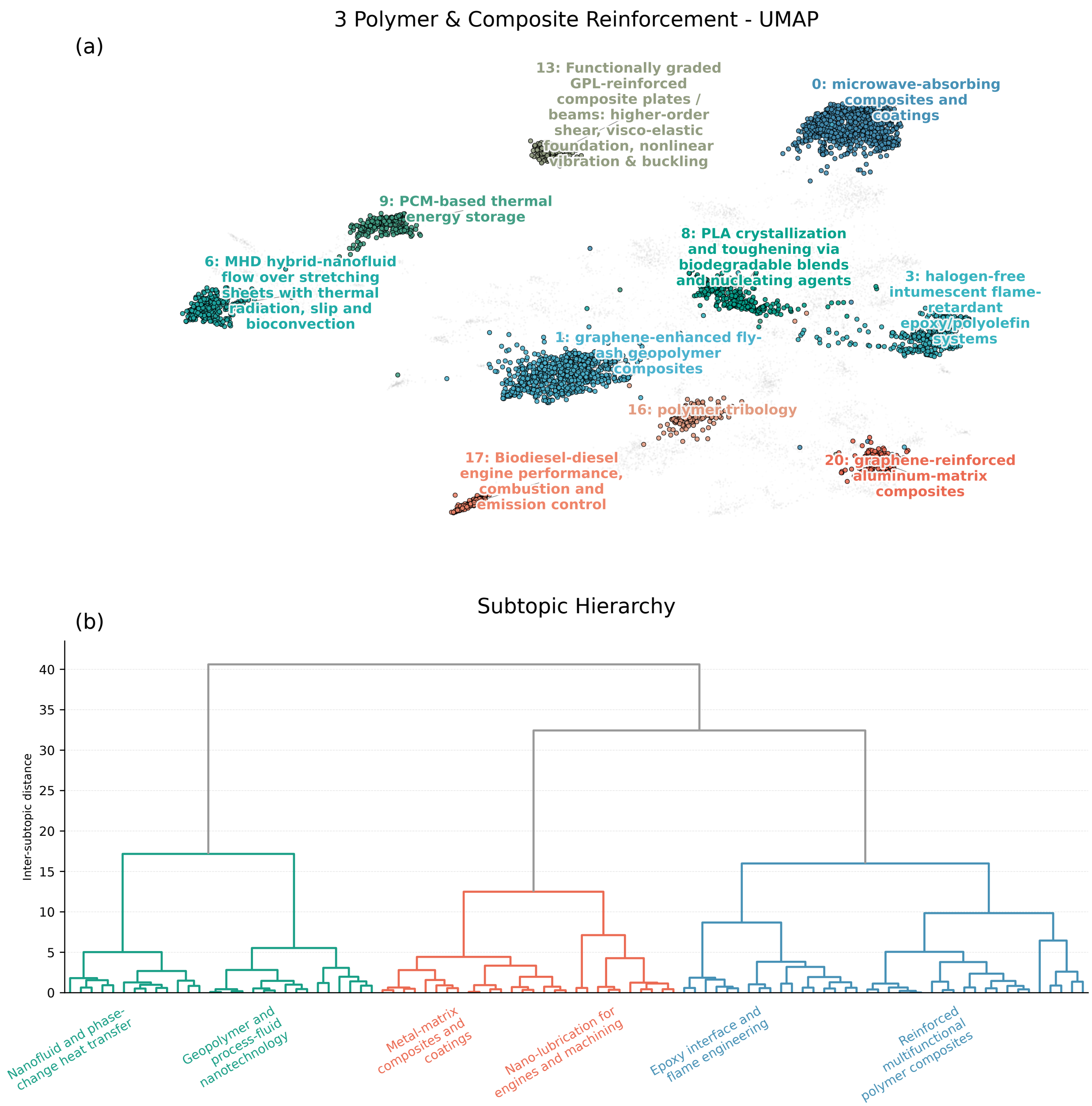

Figure S.12: a) Intertopic distance map for Polymer and Composite Reinforcement. b) Dendrogram of hierarchical clustering for Polymer and Composite Reinforcement.

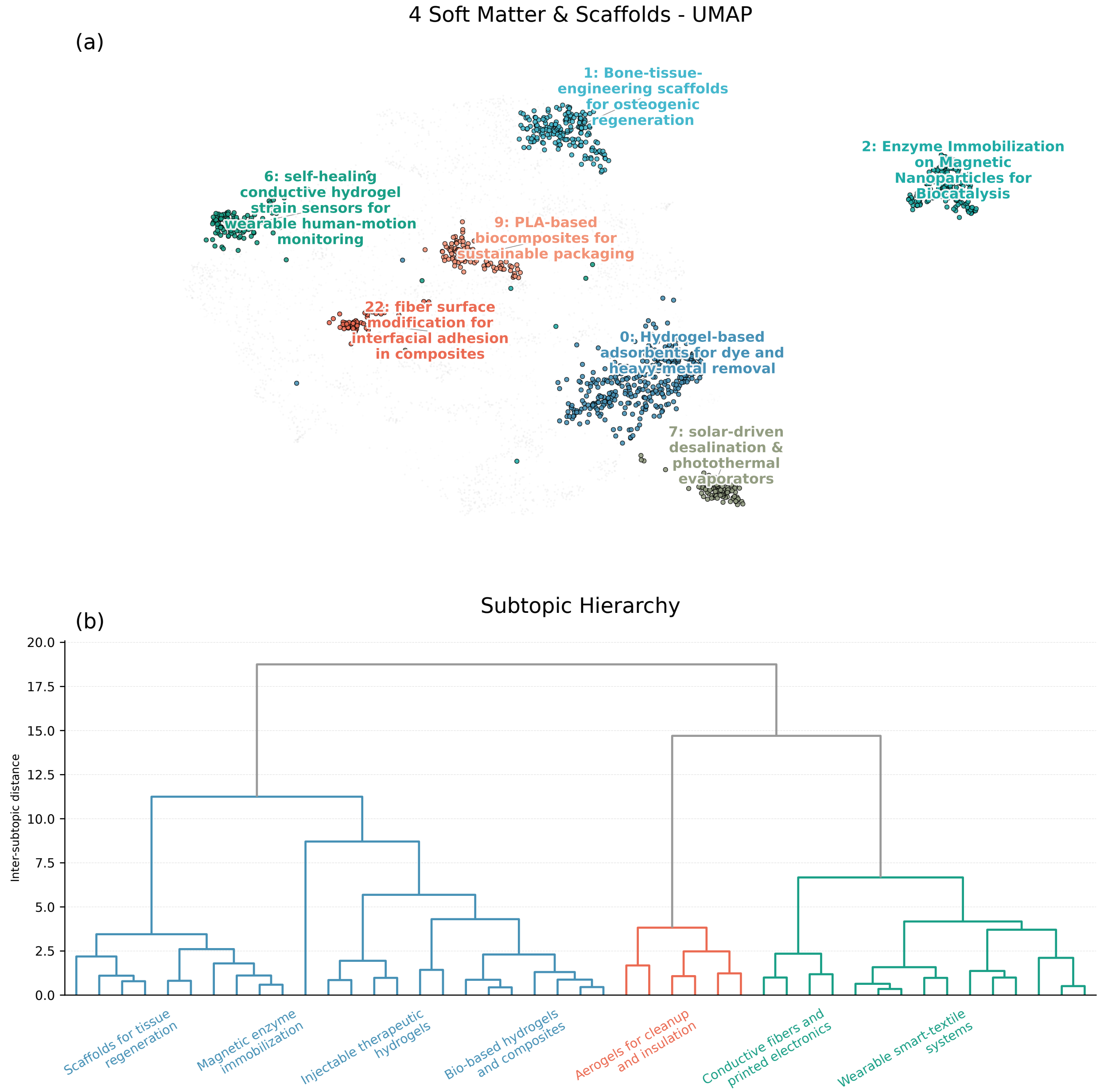

Figure S.13: a) Intertopic distance map for Soft Matter and Scaffold. b) Dendrogram of hierarchical clustering for Soft Matter and Scaffold.

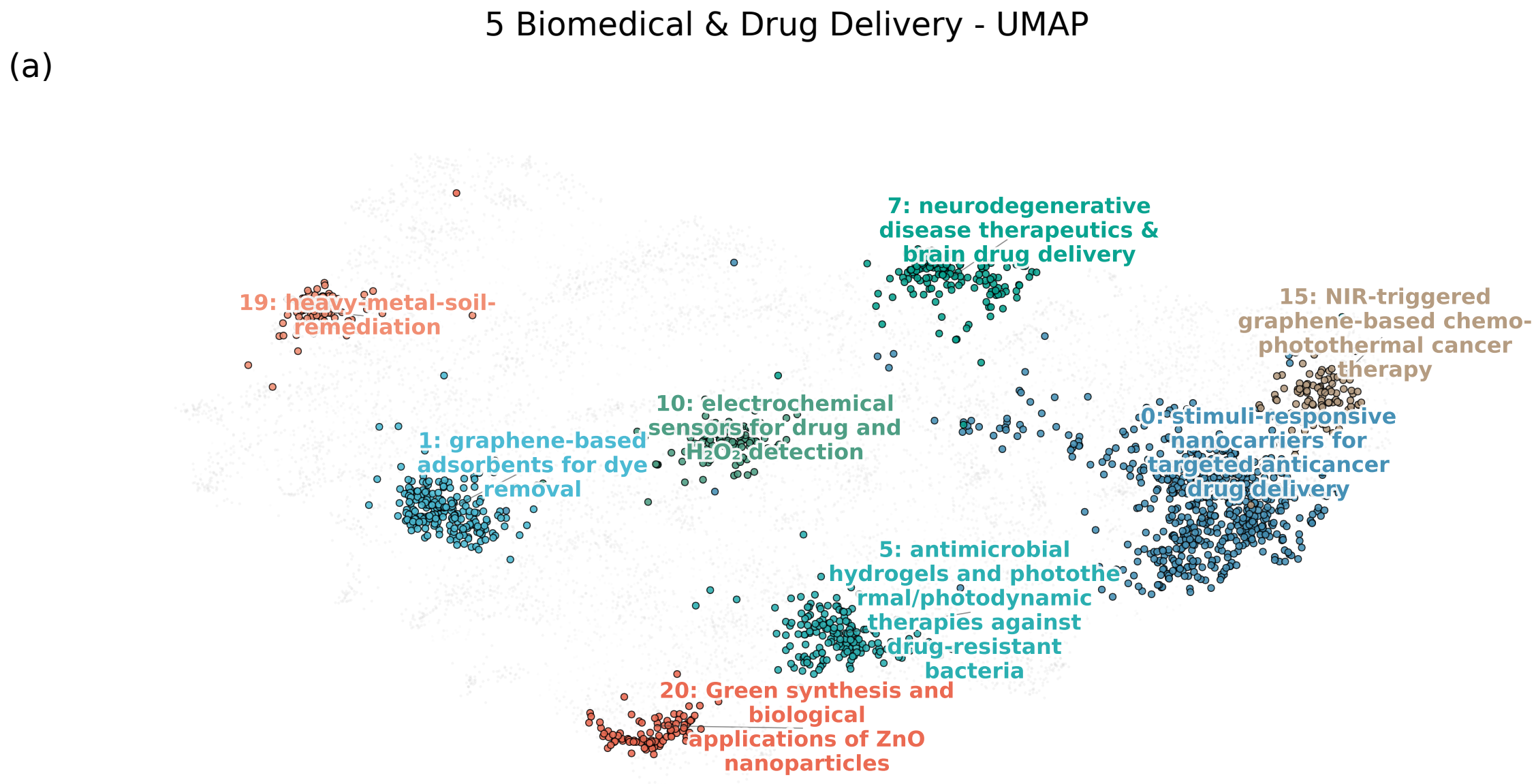

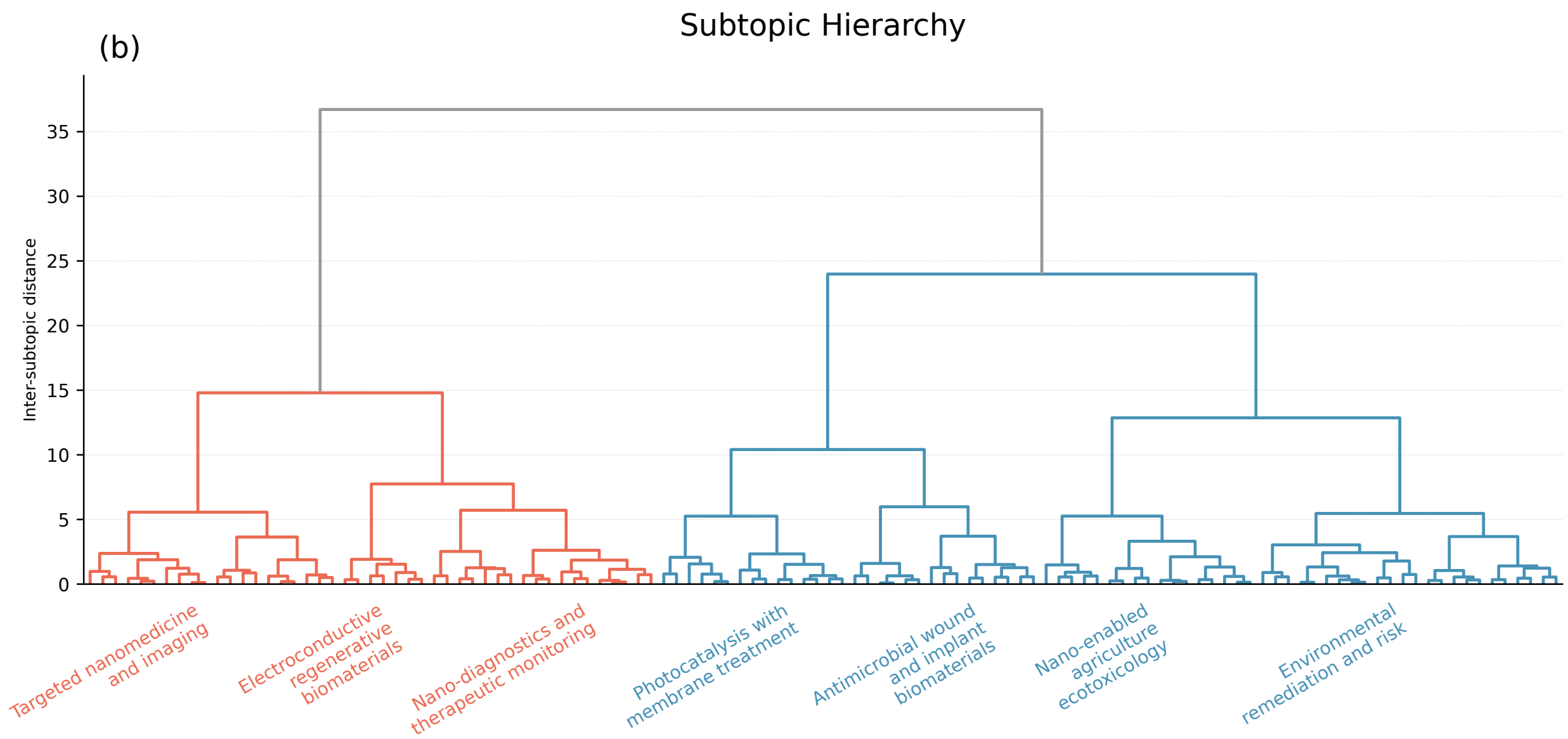

Figure S.14: a) Intertopic distance map for Biomedical and Drug Delivery. b) Dendrogram of hierarchical clustering for Biomedical and Drug Delivery.

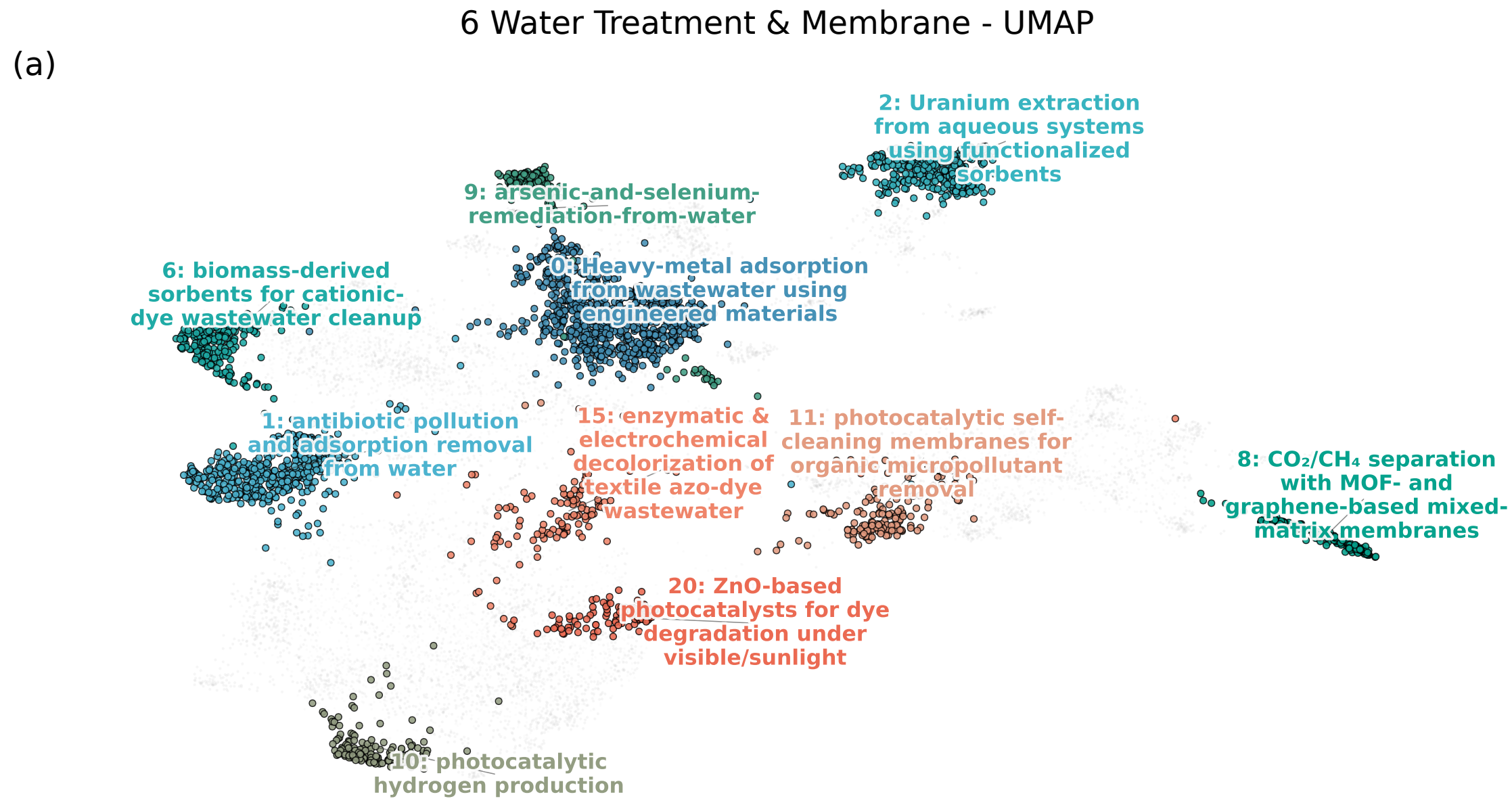

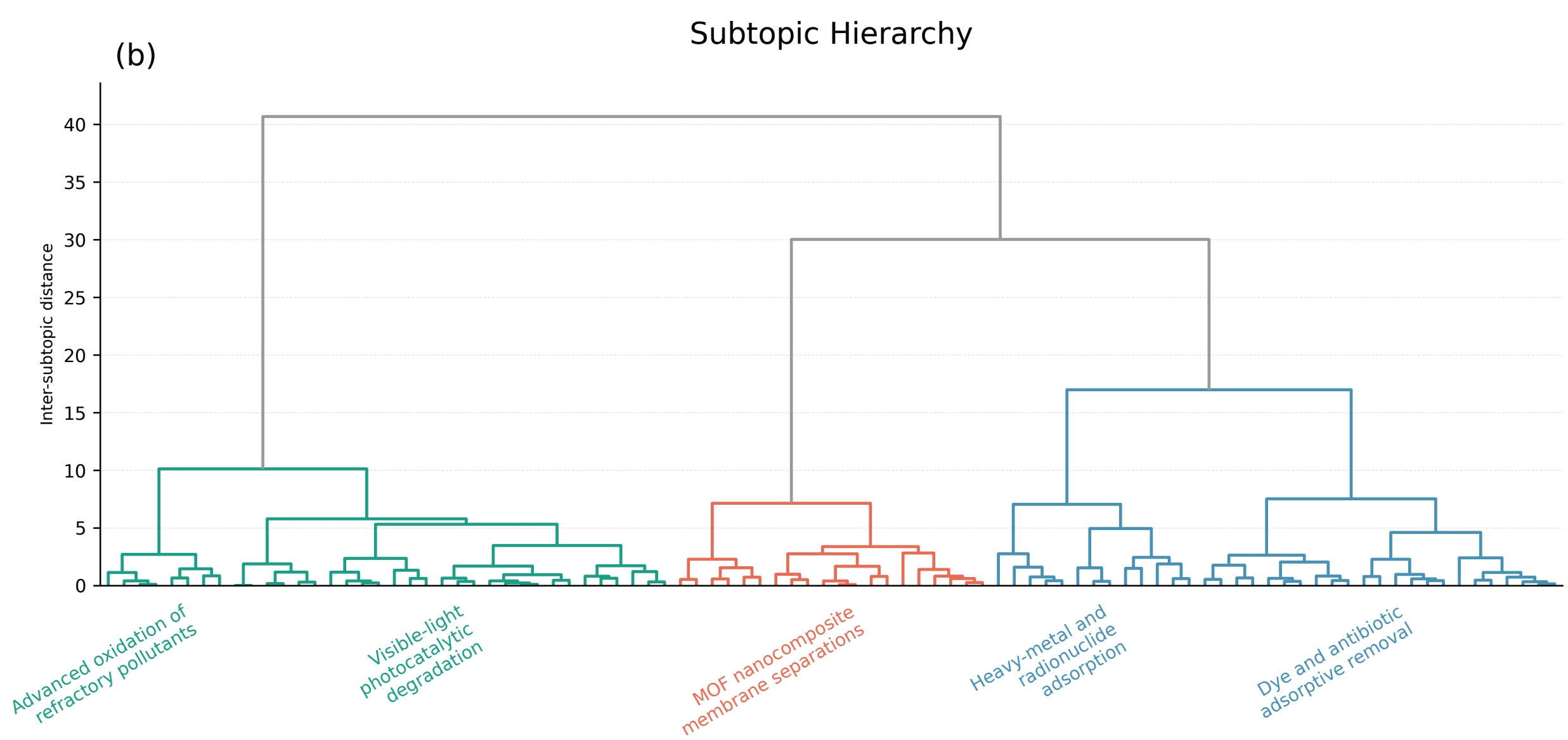

Figure S.15: a) Intertopic distance map for Water Treatment and Membrane. b) Dendrogram of hierarchical clustering for Water Treatment and Membrane.

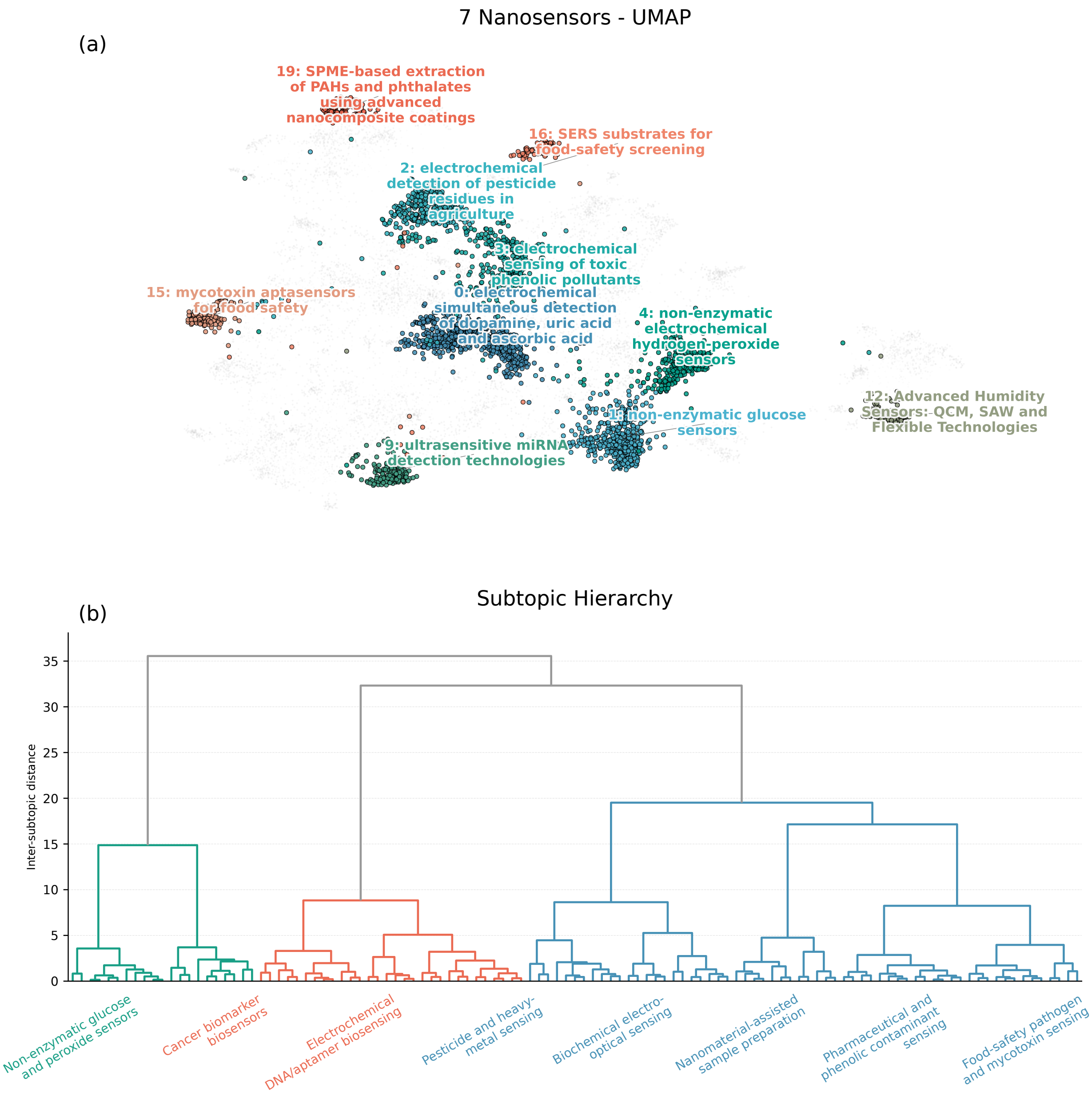

Figure S.16: a) Intertopic distance map for Nanosensors. b) Dendrogram of hierarchical clustering for Nanosensors.

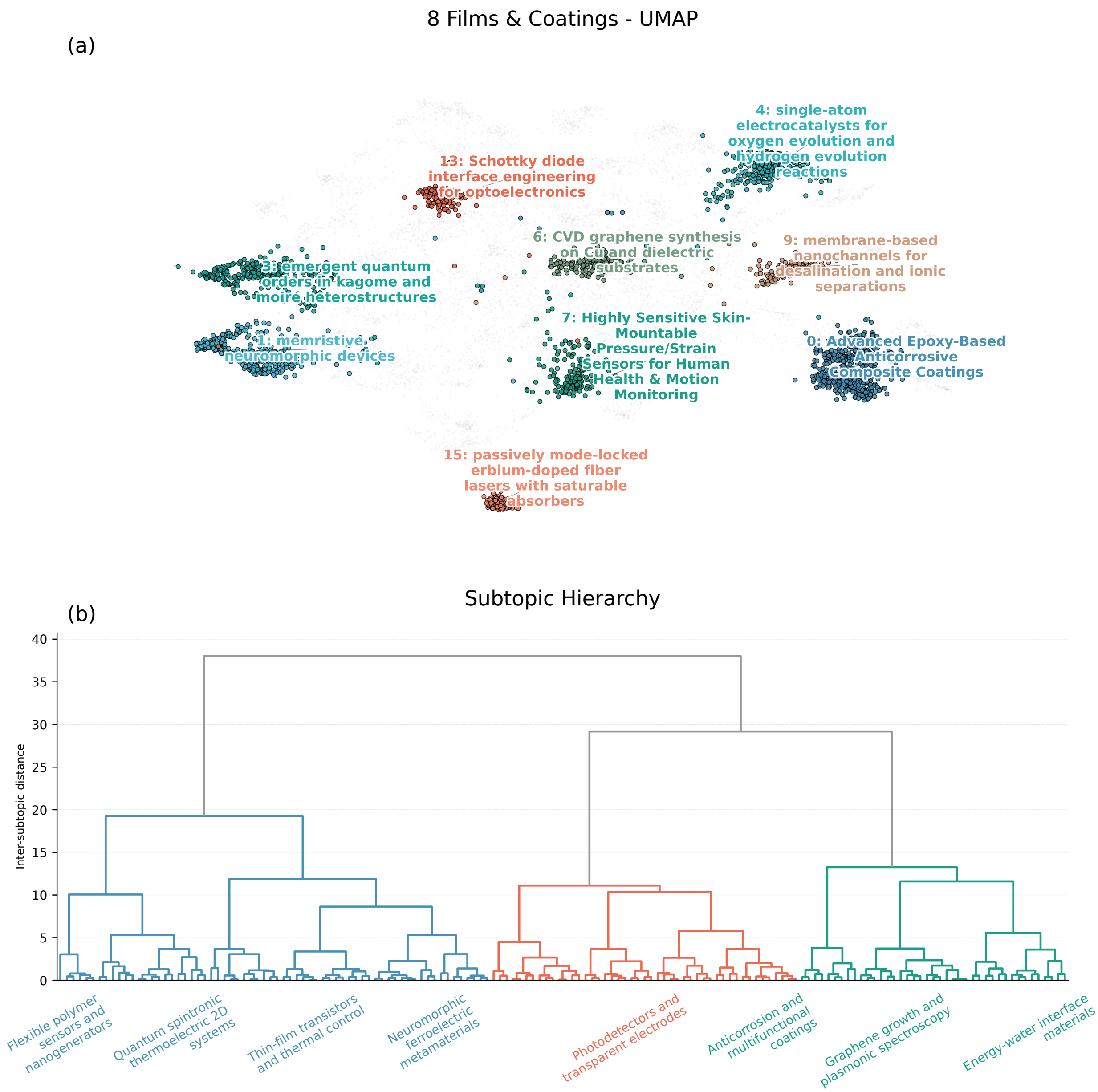

Figure S.17: a) Intertopic distance map for Films and Coatings. b) Dendrogram of hierarchical clustering for Films and Coatings.

C Parameter Extraction and Post-processing

Parameter extraction was performed using a large language model (LLM) accessed via the OpenRouter API,[S20] with kimi-k2-thinking[S21] as the default model, we configured the OpenRouter API to not use endpoints that would keep the data being sent. The model was configured with extended reasoning enabled, allowing it to produce internal chain-of-thought before generating its final structured output. Each inference call followed a standard chat completion format: a fixed system prompt establishing the model's role was provided as the system message, and the preprocessed text of each article was provided as the user message. Before being passed to the model, each article's raw text underwent a token-reduction preprocessing stage. PDF-to-text artifacts such as hyphenated line breaks were corrected, and the references and bibliography sections were removed. The paragraphs were then filtered to retain only the leading abstract-level content and those containing numeric values or domain-specific keywords related to the synthesis conditions, material characterization, and performance metrics. A hard character limit of 60,000 characters was applied as a final truncation step.

The system prompt was engineered to constrain the model to act as a scientific information extractor with strict scope rules (Section C.1). The model was instructed to extract only parameters belonging to the article's own reported experiments, explicitly excluding values cited from the prior literature or stated as general background. Each extracted parameter was required to conform to a defined schema including a canonical `snake_case` name, a list of synonyms as they appear in the text, a role classification (input, condition, material_property, or output), a data type, units, a representative example value copied verbatim from the text, the specific material to which the parameter applies, and a direct evidence sentence; these constraints reduce model hallucinations. The model was additionally instructed to summarize the material production process as a chronological list of synthesis steps. The output was required to be strictly valid JSON with no surrounding prose or markdown.

Inference was executed in parallel to process large collections of articles efficiently. In

standard mode, a thread pool distributed work across multiple workers, with concurrency defaulting to the number of available CPU cores. A high-throughput asynchronous mode was also available, using Python's asyncio event loop with a semaphore to sustain up to 100 concurrent API requests simultaneously and a per-article timeout of five minutes. The raw model output was parsed and normalized programmatically after each call, with error records written for any response that was empty or could not be decoded as valid JSON, ensuring that downstream processing pipelines received a stable, consistent data structure for every article regardless of model compliance.

C.1 LLM Prompt

System Prompt

```
You are an expert scientific information extractor.


Task
Extract experimentally meaningful parameters from the provided article text
↪  and output VALID JSON only.


What counts as a "parameter"
A parameter is any quantity/setting/value that THIS article varies,
↪  controls, defines, or measures, and that could be used in a model,
↪  including:
- Synthesis conditions (e.g., temperature, time, pH, precursor ratios,
↪  concentrations, atmosphere)
- Post-processing/processing conditions (e.g., annealing, drying,
↪  sonication, stirring rate, filtration pressure)
- Material descriptors measured in this work (e.g., surface area, particle
↪  size, zeta potential, thickness)
```

```
- Performance metrics reported for experiments in this work (e.g., capacity,
↪  efficiency, flux, rejection rate)

Critical scope rule (do NOT violate)
ONLY extract parameters that belong to THIS article'S own
↪  experiments/results.
Do NOT extract values that are:
- Cited from prior work ("reported previously", "literature value", "as
↪  reported by X et al.")
- General background/typical values ("typically", "commonly", "in general")
- Purely theoretical/speculative unless the article explicitly uses them as
↪  inputs/outputs for its own study

Output requirements (strict)
- Output JSON ONLY. No markdown, no explanations, no extra keys.
- Use double quotes for all JSON strings. No trailing commas. Ensure valid
↪  JSON.
- Include ALL required fields for every parameter object.
- Prefer extracting fewer parameters over inventing/guessing.

Canonicalization rules
- name: invent a short, machine-friendly canonical name in snake_case (e.g.,
↪  annealing_temperature, initial_ph, salt_concentration).
- synonyms: list short phrases exactly as they appear in the article for
↪  that parameter (can include symbols/abbreviations like "T", "RH",
↪  "TMP").
- De-duplicate: if the same concept appears multiple times, create ONE
↪  parameter entry and add variants to synonyms.
```

```
Role (choose exactly one)
- input: intentional controllable "knob" or recipe variable (precursor identity/ratio, dopant fraction, feed concentration)
- condition: experimental environment/operational condition (temperature, time, pH, pressure, atmosphere, flow rate)
- material_property: measured/characterized property of a material in this work (BET area, thickness, zeta potential)
- output: measured performance/result metric (flux, rejection, yield, efficiency, capacity)

data_type (choose exactly one)
- numeric: numbers, ranges, meansd, scientific notation (store as a string in example_value)
- categorical: discrete labels/classes/material identities (e.g., "N2", "air", "argon", "GO", "PVDF")
- boolean: true/false statements (use "true" or "false" as strings in example_value)
- text: free-form textual values that are not clean categorical labels

units and example_value
- units: if numeric, put the unit string exactly as used (e.g., "C", "h", "min", "mg/L", "m2/g", "bar"); if none is stated, use null.
- For non-numeric data_type, set units to null.
- example_value: ALWAYS a string copied from the text (e.g., "80", "80 C", "0.5 M", "10-20", "1.2  0.1", "argon").
  - If multiple values exist, choose one representative value that appears clearly in the text.
```

```
material field (be very specific)
- material: a SHORT noun phrase identifying the exact material/component the
↪ value is about, using the article's wording when possible.
  - Prefer the nearest explicit material mention in the same
   ↪ sentence/paragraph as the value (e.g., "TiO2 nanoparticles", "GO
   ↪ membrane", "PVDF binder").
  - If the value is for a component of a system, label the component
   ↪ explicitly:
    - Electrochemistry: use "cathode", "anode", "electrolyte", "separator"
     ↪ (or more specific if stated, e.g., "1 M LiPF6 electrolyte").
    - Separations: use "membrane", "feed solution", "permeate", "adsorbent"
     ↪ (or more specific if stated, e.g., "NaCl feed solution", "PA
     ↪ membrane").
    - Catalysis: use "catalyst" (or specific catalyst name), "reactant
     ↪ stream", "support".
  - If the text explicitly states multiple materials/samples and the same
   ↪ parameter/value applies to each, either:
    - Keep ONE parameter object with material set to a concise collective
     ↪ label that appears in the article (e.g., "all samples", "all
     ↪ membranes"), OR
    - Create one object per material only if the article gives per-material
     ↪ evidence/value statements.
- If the same parameter name applies to multiple materials/samples with
↪ DIFFERENT values, you MUST create separate parameter objects:
  - Same name
  - Different material
  - Different example_value and evidence_sentence for each
```

```
- If the article does not specify what material/component a value refers to
↪ (i.e., no clear noun phrase can be attached), set material to
↪ "not_specified".

evidence_sentence
- Provide a short direct exact quote from the article text that includes the
↪ value (and ideally the unit).

materials_production_process
Summarize the overall experimental procedure used to produce the material(s)
↪ in chronological order (synthesis + post-processing).
Include only explicitly stated steps, reagents, and conditions
↪ (temperatures, times, atmospheres, concentrations).
Do NOT invent missing details.
If the article does not specify a materials production process, set
↪ "materials_production_process": null.
If a production process is described, ALWAYS output it as a LIST of objects
↪ (even if there is only one material/process).

Output JSON schema (MUST match exactly)
Note: "materials_production_process" MUST be exactly one of:
- null (if no production process is described anywhere in the article)
- a list of objects (length >= 1), one per material/system, each with keys
↪ {"material","summary","evidence_sentence"}
- material field in "parameters" and "materials_production_process" MUST
↪ match exactly.

Schema example (list form; use this even for a single material):
```

```
{
  "materials_production_process": [
    {
      "material": "...",
      "summary": "...",
      "evidence_sentence": "..."
    }
  ],
  "parameters": [
    {
      "name": "...",
      "synonyms": ["..."],
      "role": "input|condition|material_property|output",
      "data_type": "numeric|categorical|boolean|text",
      "units": "...",
      "example_value": "...",
      "material": "...",
      "evidence_sentence": "..."
    }
  ]
}
```

C.2 Sampling Sufficiency and Stability

The parameter-network summaries were evaluated for sampling sufficiency by repeatedly subsampling articles from the final topic-level extraction sets and comparing the subsampled parameter and edge rankings with the full topic-specific reference networks (Figure S.18). Reference networks were capped at 1,100 articles per topic to keep the comparison

balanced across application domains. The analysis tracked recovery of the top 50 parameters, Jaccard similarity of the top 50 parameter sets, recovery of the top 50 co-reporting edges, and correlation of edge weights relative to the reference network.

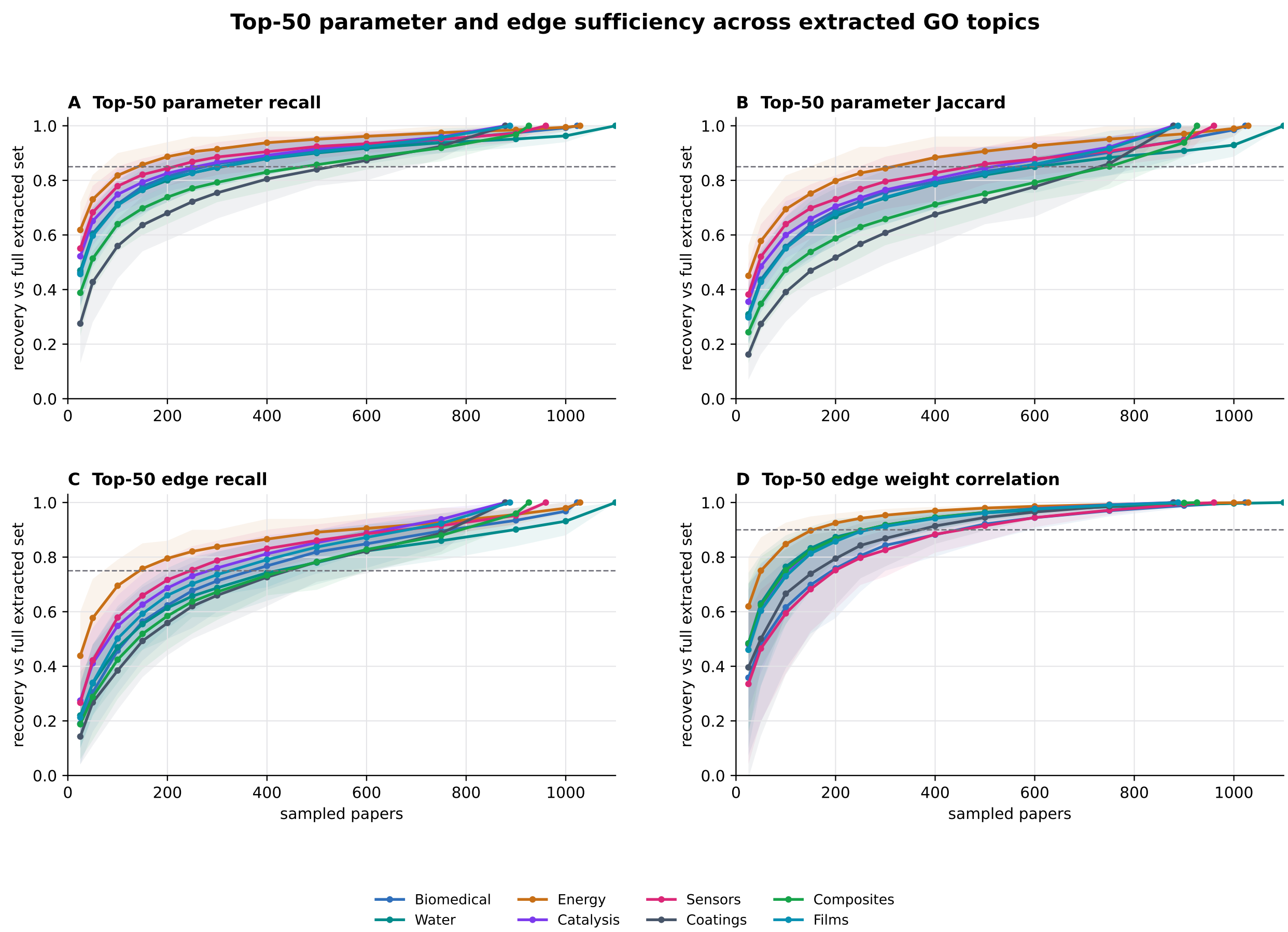

Figure S.18: Sampling sufficiency and stability of topic-level parameter networks. Curves compare subsampled article sets against the full topic-level reference networks using top-parameter recall, top-parameter Jaccard similarity, top-edge recall, and edge-weight correlation.

Across all eight topics, top-50 parameter recall reached 0.85 after approximately 150–600 articles, while top-50 parameter-set Jaccard similarity reached 0.85 after approximately 400–750 articles. Top-50 edge recall reached 0.75 after approximately 150–500 articles, and edge-weight correlation reached 0.90 after approximately 200–500 articles. At 600–750 articles, the top-50 parameter recall range was 0.873–0.975 and edge-weight correlation was 0.965–

0.993, indicating that the highest-frequency parameters and strongest co-reporting edges were stable at the sampling depths used for the topic-level analysis.

C.3 Normalization Post-processing

Compounds and Parameters

During the LLM extraction, we observed that the information extraction task remained challenging. Although the prompt explicitly guided the model to identify and classify the compound related with each parameter, the extraction produced a very large number of distinct material-related terms, reaching tens of thousands of variants (see "Unique Materials" in Figure S.19). This high terminological variability indicates that the model was able to capture material descriptions present in the text, but was not fully able to consolidate them into a stable and consistent set of GO-based material categories. The raw extraction records can preserve material names as written in the source articles, which is useful for traceability but produces a long-tailed vocabulary containing formula variants, abbreviations, composite names, and sample labels. That is one of the reasons for the growth observed with dataset sampling in Figure S.19, i.e., due to the statistical tail, following Zipfs Law, since we are counting unique words rather than the actual proportion in which each word appears. In other words, there are many tail words that are used (extracted) only once. In practice, the LLM can preserve specific name or also may create new names for materials, for example, by concatenating characteristics discussed in the article text, such as GO device 1, GO device 2, rGO sample A, or rGO at pH 6 to label the material. Because the proto-ontology does not impose strongly restrictive constraints on every possible material name, and the LLM operates with a new context memory for each extraction, a standardized normalization of material names during the extraction process could not be consistently achieved.

To address this limitation, we introduced a heuristic normalization step as post-processing to group the highly variable material-related terms extracted by the LLM into broader and more interpretable material classes (see "Merged Unique Materials" in Figure S.19).

We addressed only the cases that could be resolved through deterministic rule-based post-processing operating at the keyword level, using a sequence of heuristics. For example, we considered as GO only the materials described by terms graphene oxide, GO(s), NGO(s), MGO(s), N-GO(s), and M-GO(s). For rGO, we considered reduced graphene oxide, reduced GO(s), RGO(s), R-GO(s), and NRGO(s). These classes were defined based on categories already well established in the graphene oxide literature, such as graphene oxide, reduced graphene oxide, composites, and other related carbon materials. This procedure allowed us to reduce the terminological variability produced during extraction and to obtain a more consistent classification of the materials used in each article, enabling the subsequent analysis of material-category distributions across the LDA topics.

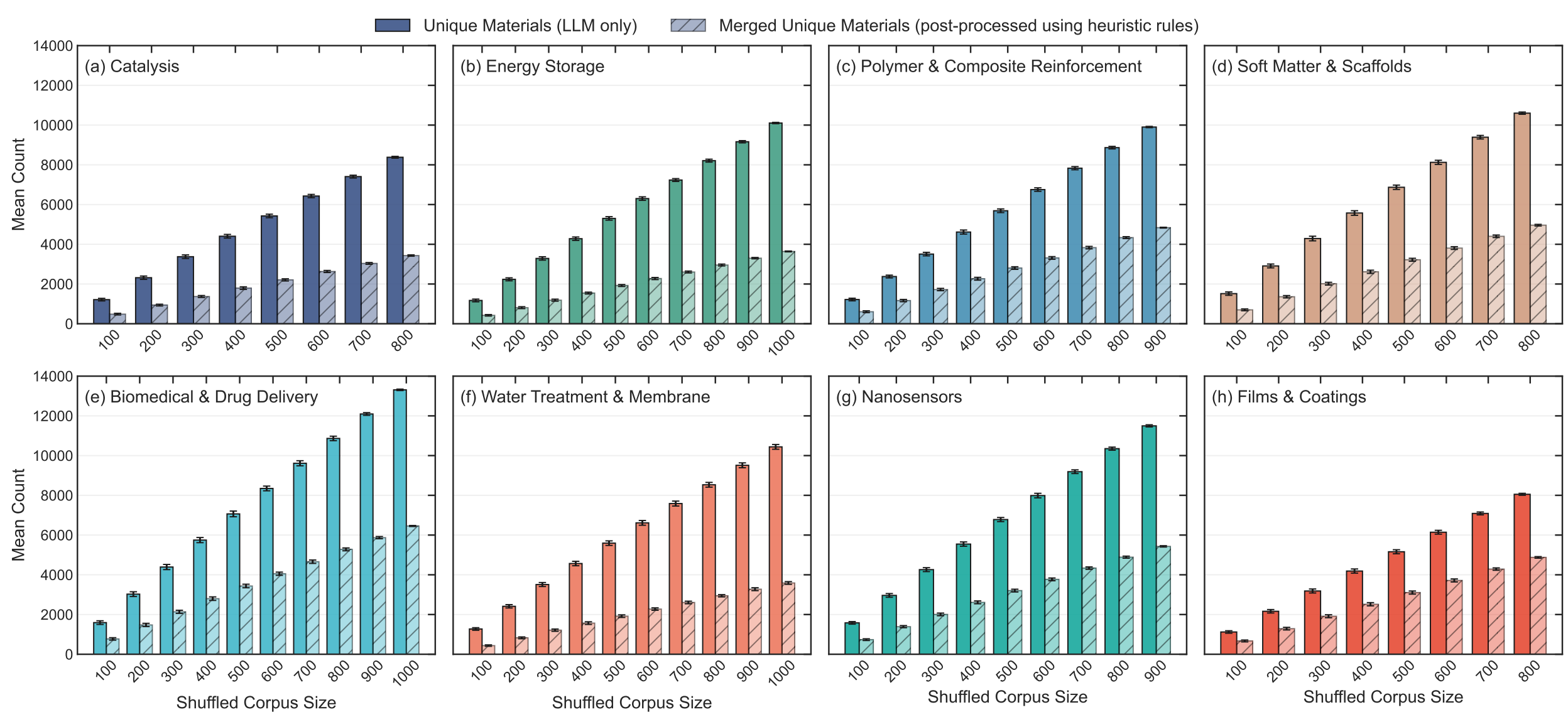

Figure S.19: Sampling behavior of extracted material names before broad class interpretation. The accumulation of unique material strings reflects the long-tailed naming structure of the GO literature and motivates the use of a rule-based heuristic for a deterministic material-class normalization for aggregate summaries.

From the perspective of the computational implementation of the classification heuristics, regular expressions (regex) were employed within a cascade of if-else rules. It is also important to note that the literature itself still preserves a certain degree of ambiguity regarding the material nomenclature. Not every study addresses the number of layers in order to

distinguish multilayer graphene oxide from few-layer graphene oxide or monolayer graphene oxide. Throughout the literature, specific acronyms may also be used to refer to these samples, which further complicates the ability of automatic tools to correctly interpret them. Our current understanding, after conducting this work, is that the proto-ontology embedded in the LLM prompt must be constructed with a high level of specificity in order to properly guide the extraction toward the target property associated with the target class of materials (this aspect is also discussed below).

The same type of behavior is observed for the extracted parameters, as shown in Fig. S.20. In this case, however, it is not possible to apply the same post-processing technique because the space of possible words is substantially larger. This is due to the much greater diversity of synthesis and characterization parameters that may be explored throughout the literature, which is precisely the target of our discovery process. Although different materials may also generate a broad vocabulary (as well as the concatenation of descriptive qualities used by the LLM in some cases to generate names), we already knew beforehand that the literature would primarily involve GO and related carbon-based materials. Consequently, our rule-based normalization may exclude some distinct materials if the extracted LLM-generated name does not explicitly contain carbon-related terminology. The same type of trade-off would not be acceptable for parameters, since discovering them without imposing prior knowledge is exactly the objective of the analysis.

To address this issue, we needed to employ an automatic merging strategy capable of capturing the semantic level of the terms. For this reason, we adopted cosine similarity between embeddings generated from sentence (strings) obtained by concatenating the extracted parameter name with a list of synonyms, which the LLM was also instructed to extract within the same extraction prompt. Cosine similarity (Eq.S.1) measures the angular similarity between two sentence embeddings constructed in this way, with values closer to 1 indicating greater semantic similarity.

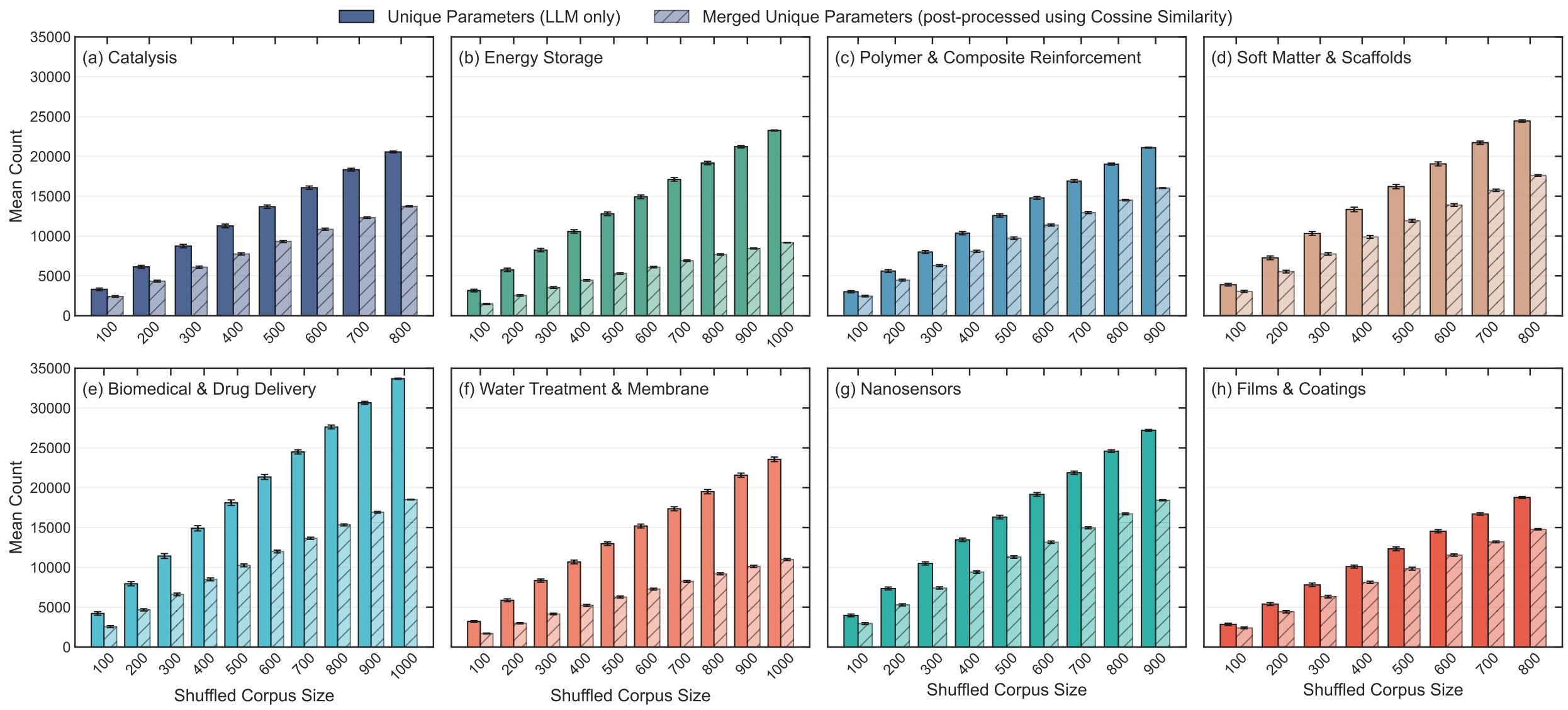

Figure S.20: Sampling behavior of extracted parameter names. The accumulation of unique parameters strings reflects the long-tailed naming of the GO literature and motivates the use of deterministic (cosine similarity) parameter-class normalization for aggregate summaries.

$$\text{CosineSimilarity}(\mathbf{x}, \mathbf{y}) = \frac{\mathbf{x} \cdot \mathbf{y}}{\|\mathbf{x}\| \, \|\mathbf{y}\|} = \frac{\sum_{i=1}^{n} x_i y_i}{\sqrt{\sum_{i=1}^{n} x_i^2} \sqrt{\sum_{i=1}^{n} y_i^2}} \tag{S.1}$$

A similarity threshold of 0.8 was adopted to identify semantically equivalent parameter representations. The most common name of a parameter in a merge list (words that have more than the threshold similarity) was used to name the merged parameter. The embeddings were obtained from pretrained Qwen3-8B-Embedding.[S17]

Values and Units

In addition to the normalization of material names and parameter names, it was also necessary to normalize the numerical values and units associated with the parameters selected for analysis. The extracted value stored in the corresponding field (`example_value`) of the extracted JSON object may include, in addition to the numeric value itself, the physical unit, a reference to the material, or scientific notation in textual form. Occasionally, the physical

unit recorded in the unit field does not correspond to a standard unit for that parameter, either due to extraction errors or inconsistencies in the source article. For example, mg/g was the most common unit, while cases of g/cm or mol/L were less frequent, which could even indicate absorption instead of adsorption. For the construction of Fig.9 (main text) and S.21 (SI), these cases were programmatically removed through a validation filter, without the need for manual intervention on a case-by-case basis. The code extracts numeric values from text strings representing experimental measurements and normalizes their units. It handles ranges by averaging, converts scientific notation (including Unicode superscripts) into standard numeric form, removes textual units, and identifies whether a unit represents a mass-per-mass ratio (e.g., mg/g, g/g). It then filters the dataset to include only positive mass-per-mass values and standardizes them to mg/g by converting values reported in g/g. This process has been done for the three analyzed parameters (adsorption capacity, BET surface area, and specific capacitance). The computational implementation was based in heuristics and regular expression techniques.

In Fig. S.21 the distribution of sorption capacity, BET surface area, and specific capacitance is shown, considering only the outliers for each adsorbent material (without merging). These outliers are defined as values greater than the third quartile plus 1.5 times the interquartile range (IQR)[S22] (i.e., Q3 + 1.5 IQR). It can be observed that many materials are composites containing some form of carbon-based material, especially GO. Extraction-related characteristics also emerge, such as the addition of the word "electrode" to most materials associated with the specific-capacitance parameter. For adsorption capacity, the adsorbate itself sometimes appears alone as the extracted material term (a more detailed analysis of some of these cases is provided in the following performance-evaluation section).

C.4 Extraction Quality and Validation

The parameter-extraction pipeline was checked with automated source-grounding tests designed to detect unsupported values, missing evidence, and severe text-conversion artifacts

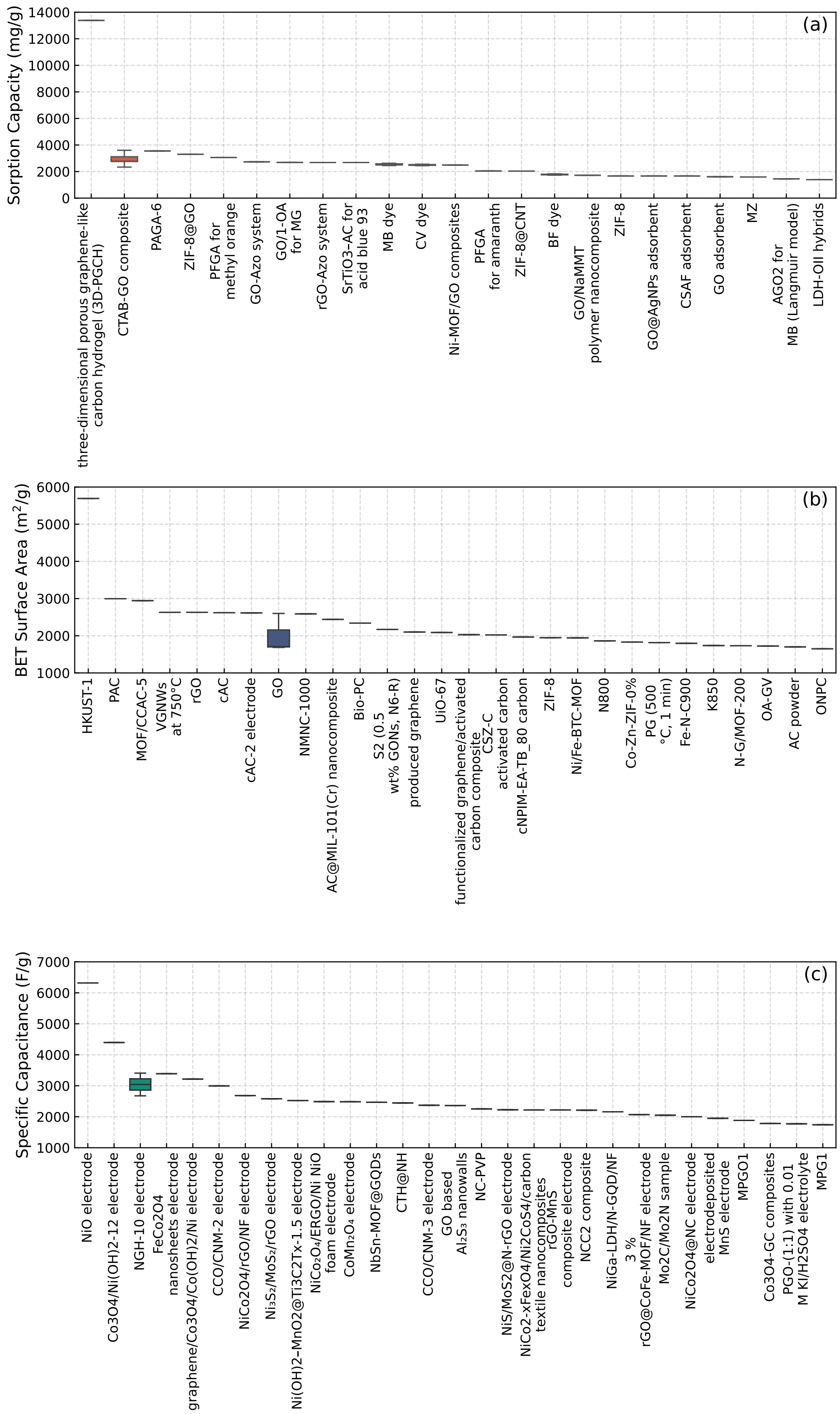

Figure S.21: Distribution by material without class merging for the top 30 highest values of: (a) Sorption Capacity; (b) BET Surface Area; and (c) Specific Capacitance.

before downstream analysis. Each extracted record was normalized and compared against the cleaned source text. A value was counted as grounded when its reported value and unit, after normalization of whitespace, punctuation, Unicode variants, ranges, and scientific notation, could be matched to the source text. An evidence sentence was counted as grounded when the extracted evidence sentence overlapped substantially with the source passage after stopword removal. For numeric rows, filtered numeric coverage was computed after removing rows whose source text or extracted value could not support a reliable numeric comparison.

Across 284,453 extracted parameter rows, 276,149 values were source-grounded (97.1%), 280,181 evidence sentences were source-grounded (98.5%), and 272,235 complete records passed both checks (95.7%). The filtered numeric-coverage check retained 198,873 of 211,469 numeric rows (94.0%). Severe PDF-to-text artifacts were concentrated in a small subset of source files; 166 of 7,507 source files (2.2%) were omitted from the filtered quality-control view. These 166 articles contained severe text extraction issues from the pdf-to-text conversion. We also excluded articles that were reviews of the literature from the analysis. In the coatings, optical, and corrosion subset used for detailed validation, the corresponding complete-record grounding rate was 93.8%, with 95.3% value grounding, 98.3% evidence grounding, and 90.6% filtered numeric coverage. These checks do not replace manual expert curation of every extracted value, but they provide a corpus-scale estimate that the extracted parameter records are predominantly grounded in the source text.

To better understand how the LLMs extract values according to the proto-ontology encoded in the extraction prompt, we selected a subset of adsorption-capacity outliers for manual validation against their corresponding source articles. The Table S.2 presents the selected material/value pairs used in a manual validation together with their respective DOI identifiers. All numeric values and materials are correctly extracted, although the extraction process does not always accurately preserve the corresponding adsorbent–adsorbate pair association. Some values were additionally annotated with comments regarding the extraction process.

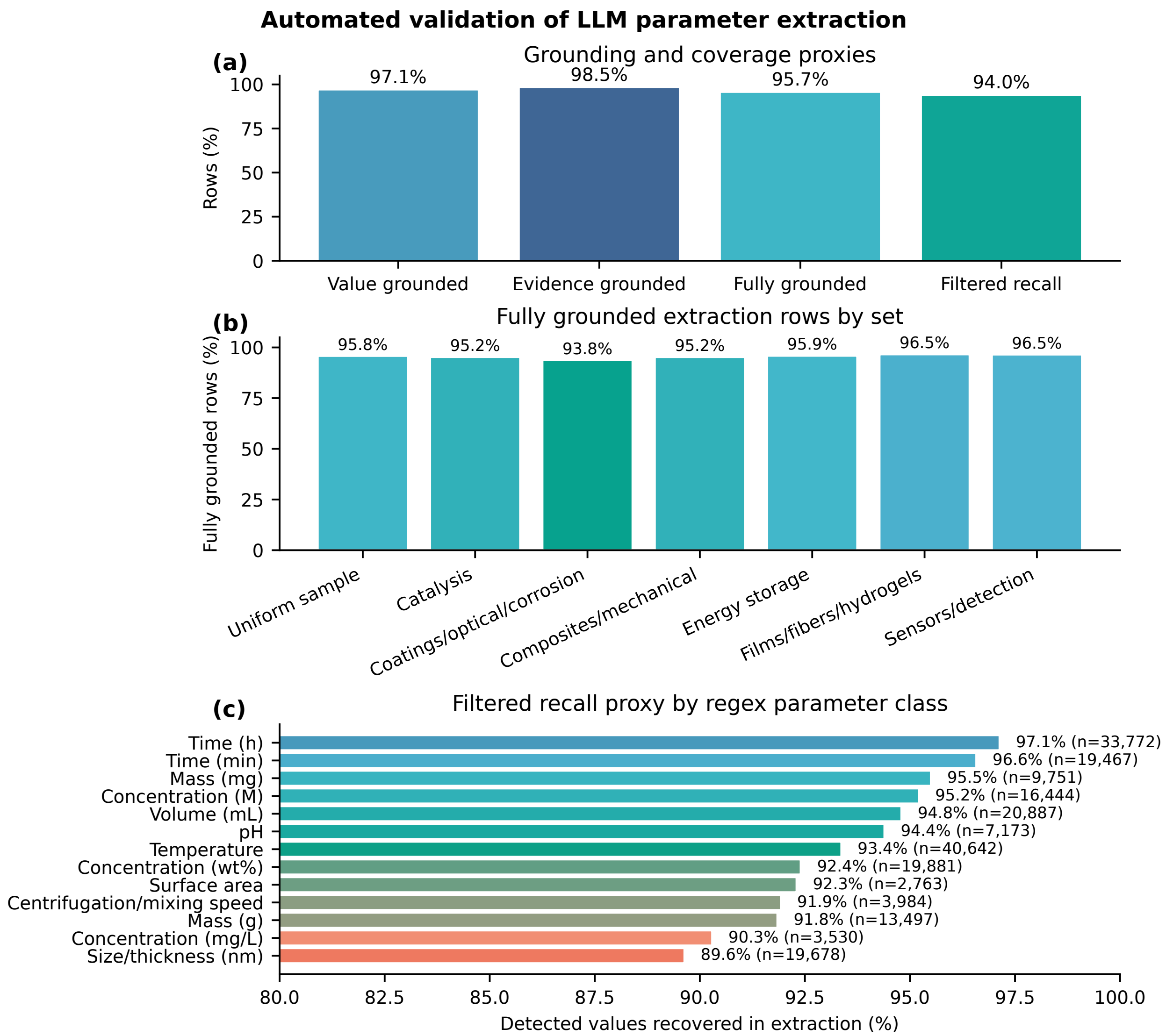

Figure S.22: Automated source-grounding quality control for extracted parameter records. The checks compare extracted values, units, and evidence sentences against cleaned source text and report value grounding, evidence grounding, complete-record grounding, and filtered numeric coverage after excluding severe text-conversion artifacts.

Table S.2: Adsorption capacities extracted from the literature.

Material	Value (mg/g)	DOI
MB on MrGO/TiO2	675.9[1]	10.1016/j.apsusc.2015.11.075
MrGO-2	625	10.1016/j.apt.2018.12.005
MB-LIG system	926	10.1016/j.carbon.2017.08.079
GO/1-OA for ER	1189.1	10.1016/j.cej.2018.04.153
GO/1-OA for MG	2687.56	10.1016/j.cej.2018.04.153
PAGA-6	3550	10.1016/j.cej.2020.125272
MZ	1590.28	10.1016/j.cej.2020.125952
AGO2 for MB at pH 10	634.3[2]	10.1016/j.chemosphere.2020.127736
AGO2 for MB (Langmuir model)	1448.2	10.1016/j.chemosphere.2020.127736
PPD-GO	892.8	10.1016/j.colsurfa.2016.08.084
PPD-GO	980.4	10.1016/j.colsurfa.2016.08.084
PPD-GO	1061	10.1016/j.colsurfa.2016.08.084
CTAB-GO composite	1309[3]	10.1016/j.colsurfa.2018.06.048
CTAB-GO composite	2335[3]	10.1016/j.colsurfa.2018.06.048
CTAB-GO composite	2767[3]	10.1016/j.colsurfa.2018.06.048
CTAB-GO composite	3116[3]	10.1016/j.colsurfa.2018.06.048
CTAB-GO composite	3602[3]	10.1016/j.colsurfa.2018.06.048
MGO@CD-CA-HA	847.4	10.1016/j.colsurfb.2020.111510
GO-AL aerogel	1142.43	10.1016/j.ijbiomac.2019.12.017
GO-AL aerogel	1158.52	10.1016/j.ijbiomac.2019.12.017
GO	714	10.1016/j.jcis.2011.02.064
APAM/GO aerogels	781.5[4]	10.1016/j.jcis.2015.04.042
MV dye	1324[5]	10.1016/j.jcis.2020.06.083
BF dye	1825[5]	10.1016/j.jcis.2020.06.083

Table S.2 continued

Material	Value (mg/g)	DOI
CV dye	2441[5]	10.1016/j.jcis.2020.06.083
MB dye	2454[5]	10.1016/j.jcis.2020.06.083
WTR-SO3H for MB adsorption	833[6]	10.1016/j.jece.2018.04.058
WTR-SO3H for MB adsorption	834[6]	10.1016/j.jece.2018.04.058
GO adsorbent	1335	10.1016/j.jece.2019.103106
mCS/GO-QAS	647[7]	10.1016/j.jece.2020.103820
MPrGO	1105.8	10.1016/j.jhazmat.2019.121942
GO adsorbent	1608	10.1016/j.jwpe.2020.101367
CGS aerogel	623.9	10.1016/j.jwpe.2020.101511
CSAF adsorbent	1666.67[8]	10.1016/j.microc.2019.103966
rGO-Azo system	2682.04[9]	10.1016/j.molliq.2022.119967
GO-Azo system	2730.51[9]	10.1016/j.molliq.2022.119967
GO@AgNPs adsorbent	1666.7	10.1016/j.scitotenv.2019.136111
GO@AgNPs adsorbent	1666.7	10.1016/j.scitotenv.2019.136111
Ni-MOF/GO composites	2489	10.1016/j.ultsonch.2017.06.013
GO-A-Im	900.94	10.1016/j.ultsonch.2017.11.010

1 - The LLM extracted a value of 675.9 mg/g from the sentence: "The q_e reaches 675.9 mg/g when C_0 is 149.6 mg/L, which is larger than that of most of the traditional adsorbents [4,34]". Other values reported in the article are 845.6 mg/g and 467.6 mg/g, both for the adsorbate Methylene Blue (MB) with the adsorbents MrGO/TiO_2 and rGO/TiO_2, respectively.

2 - In one of the tables, the article uses a different nomenclature for the adsorbent: instead of AGO2, it is listed as p(AA)-g-GO. Nevertheless, it is noteworthy that the model correctly

extracted the pH value, based on the original sentence: “With the pH value increasing from 2 to 10, the adsorption capacity of AGO2 for MB continues to rise, and the equilibrium adsorption capacity from 340.5 to 634.3 mg/g”.

3 - The reported results correspond to different conditions for the same adsorbentadsorbate pair, as indicated by the original sentences: “The results demonstrated that the adsorption capacities of the CTAB-GO composite for Congo Red were 2767 mg/g, 3116 mg/g, and 3602 mg/g at 298 K, 308 K, and 318 K, respectively” and “For example, 57, 204, 471, 1309, and 2335 mg/g adsorption capacities were obtained in 25, 50, 100, 300, and 500 mg/L Congo Red solutions, respectively, which suggests that the CTAB-GO composite may be a good candidate for wastewater treatment”.

4 - The LLM extracted a value of 781.5 mg/g. This value appears in the sentence: “The absorption capacity of oven-heated composites (20% GO) is only 449.9 mg/g, while that of freeze-dried aerogels (20% GO) increases to 781.5 mg/g”. The maximum value reported in the article is 1034.3 mg/g for the adsorbent APAM/GO aerogels (anionic polyacrylamide/graphene oxide) and the adsorbate Basic Fuchsin (BF).

5 - The adsorbates were marked on the extracted material. The adsorbent was SAPD (dithiocarbamate-grafted star-like polymer). Although it is not a graphene oxide-based material, the article includes a comparison with other GO-based adsorbents, which justifies its inclusion in 120k corpus.

6 - The adsorbent is WTR (sulfonated waste tire rubber, WTR-SO_3H). The tested adsorbates were Methylene Blue (MB), Methyl Orange (MO), and Tetracycline antibiotics (TC), some of which were considered outliers and are therefore not included here. Although it is not a graphene oxide-based material, the article includes a comparison with other GO-based adsorbents, which justifies its inclusion in 120k corpus. More importantly, the values 834 and 833 correspond to two distinct forms in the adsorption models, as can be seen from the extraction sentences: “the Q_e was calculated to be about 834 mg/g at an initial MB concentration of 1000 ppm and at pH 10, Fig. 5a” and ”applying the Langmuir isotherm equation

(Eq. (3)), the maximum adsorption capacity (Q_m) of the WTR-SO3H was calculated to be 833, 555, and 476 mg/g for MB at pH 10, 7 and 3.25, respectively".

7 - The LLM extracted a value of 647 mg/g due to the sentence: "When the adsorbent concentration was 0.05 g/L, an exceptional removal capacity was reached (647 mg/g, 32%)". The value of 650 mg/g is reported in the abstract and conclusion: "After optimization, at a temperature of 298 K and pH 6, the highest removal capacity (650 mg/g) was reached at a contact time of 30 min, a nanocomposite concentration of 0.4 g/L, and a dye concentration of 600 mg/L".

8 - The adsorbate is Acid Fuchsin (AF). The adsorbent is CSAF (Chitosan/Al_2O_3/Fe_3O_4 nanoparticles). Although it is not a graphene oxide-based material, the article includes a comparison with other GO-based adsorbents, which justifies its inclusion in 120k corpus.

9 - Azo refers to the pesticide Azoxystrobin, which is the adsorbate under study. GO (graphene oxide) and rGO (reduced graphene oxide) are the adsorbents.

C.5 Material Classification Taxonomy

We applied a classification strategy for the material by applying a taxonomy based on a well established ontology of GO-related engineered materials. A two-level operational taxonomy was used. The first level is a structured description of the material production in each scientific article. It classifies if the target material is ghaphene-based, the identity of the graphene-family, the layer/dimensionality, the chemical state, the physical form/architecture, the composition class and component role (see Table S.3). This level preserves distinctions that are scientifically important but often blurred in article titles or database indexing: graphite oxide versus graphene oxide, unreduced GO versus rGO or partially rGO, explicitly stated few-layer GO, graphene nanosheets or nanoplatelets, and graphene-based composites.

We then used these descriptors to assign each target material identified to broader material categories, including GO, rGO, composites, and other carbon-based systems using heuristic rules (see Table S.4). A single extracted target material can be assigned to only

one category. For example, a GO/polymer membrane is counted as a GO-based composite, not as both "GO" and "composite." In each article, one or more material samples (e.g., GO and rGO composites) can be identified in the methodological section. Further, each material sample identified is classified individually and assigned to one mutually exclusive material category (see Table S.4). For example, GO-based composites, rGO-based composites, and graphene composites are distinguished by the graphene-family identity, and they have the same description at the composition identity (i.e., composite). If a article has 4 materials (2GO, 1rGO, 1GO/composite), we will count one for each class (GO non-composite, rGO non-composite and GO-based composite).

The taxonomy is conservative by construction. We do not infer reduction, few-layer structure, doping, or composite architecture unless the source text supports it. GO is assigned when the target material is graphene oxide and no reduction evidence is present. rGO is assigned only when the target is explicitly reduced or partially reduced graphene oxide. Few-layer GO is not treated as a separate family label; instead, it is represented as graphene oxide with few-layer dimensionality when the article states that property. Graphene-based composites are separated from GO- and rGO-based composites using the identity of the graphene-based component. Ambiguous graphene-based cases are kept in an ambiguous category instead of being forced into GO or rGO.

This entry-level design also handles multi-material articles. The article may include GO synthesis, reduction to rGO, formation of an oxide/rGO composite, and comparison to a non-graphene-based control. Those are different target-material entries and are classified separately. The final classification contains 16,995 material entries from 7,569 articles in the eight topics.

The first-step classifications were generated with LLM Kimi K2 Thinking (1T parameters). The model received the full text of the article together with the extracted `material` name, `the material-production process`, and `the evidence sentence` (JSON entries). The prompt instructed the model to classify whether the final target material (not precur-

Table S.3: Graphene-based material taxonomy categories.

**Dimension**	**Categories**
Graphene-based material	Graphene-based or not
Graphene family identity	Graphene; graphene oxide; reduced graphene oxide; partially reduced graphene oxide; graphite oxide; few-layer graphene; multilayer graphene; graphene nanoplatelet; graphene nanosheet; graphene quantum dot; graphene nanoribbon; porous graphene; holey graphene; graphene nanomesh; graphene derivative; unspecified graphene-based material.
Layer structure and dimensionality	Monolayer; bilayer; few-layer; multilayer; zero-dimensional; one-dimensional; two-dimensional; three-dimensional assembly; unspecified structure/dimensionality.
Chemical state or modification	Pristine; oxidized; reduced; partially reduced; heteroatom-doped; co-doped; covalently functionalized; noncovalently functionalized; polymer-grafted; edge-functionalized; defect-engineered; vacancy-engineered; intercalated; crosslinked; hydrogenated; fluorinated; aminated; carboxylated; sulfonated; unspecified chemical state/modification.
Physical form and architecture	Sheet; flake; nanosheet; platelet; nanoribbon; quantum dot; nanoscroll; fiber; yarn; powder; dispersion; ink; film; article; membrane; coating; laminate; foam; sponge; aerogel; xerogel; hydrogel; porous network; scaffold; monolith; unspecified form/architecture.
Composition class	Single-component graphene-based material; binary composite; ternary composite; multicomponent composite; hybrid material; heterostructure; graphene matrix composite; graphene-filled matrix; graphene-supported material; graphene-coated material; unspecified composition class.
Component role	Active material; matrix; filler; reinforcement; support; scaffold; conductive additive; conductive network; current collector; electrode; coating; barrier; membrane; adsorbent; catalyst; cocatalyst; sensor transducer; delivery carrier; thermal management additive; lubricant additive; unspecified role.

Table S.4: Operational taxonomy for classifying GO-related materials

**Operational category**	**Inclusion criteria**	**Representative terms**
GO non-composite	Family identity is graphene oxide; composition is not a composite class	graphene oxide, GO
GO-based composite	Family identity is graphene oxide and composition is binary, ternary, multicomponent, hybrid, supported, coated, or filled-matrix composite	GO/polymer, GO/metal oxide, GO/LDH, GO membrane composite, GO-supported catalyst
rGO non-composite	Family identity is reduced graphene oxide or partially reduced graphene oxide; composition is not a composite class	rGO, reduced GO, reduced graphene oxide, partially reduced GO
rGO-based composite	Family identity is reduced graphene oxide or partially reduced graphene oxide; composition is binary, ternary, multicomponent, hybrid, supported, coated, or filled-matrix composite	rGO/MnO_2, Pd/rGO, rGO/polymer, rGO electrode composite
Graphene non-composite (non-GO)	Family identity is graphene, few-layer graphene, multilayer graphene, graphene nanosheet, nanoplatelet, nanoribbon, quantum dot, porous graphene, holey graphene, nanomesh, or graphene derivative; composition is not a composite class	graphene, few-layer graphene, graphene nanosheets, graphene nanoplatelets, graphene quantum dots
Graphene composite (non-GO)	Family identity is graphene, few-layer graphene, multilayer graphene, graphene nanosheet, nanoplatelet, nanoribbon, quantum dot, porous graphene, holey graphene, nanomesh, or graphene derivative; composition is binary, ternary, multicomponent, hybrid, supported, coated, or filled-matrix composite	graphene/polymer, GNP/epoxy, graphene quantum dot composite, graphene-coated substrate
Graphene-based composite, family unspecified	The target is explicitly graphene-based and composite, but the article evidence does not support assignment in GO, rGO, or non-GO graphene-family identity.	graphene-based composite, carbon/graphene hybrid where family identity is not specified
Graphene-based, family and composition unspecified	Ambiguous cases use the field "unspecified" in all respective dimensions	device, mixed material lists
Non-graphene-based target	The extracted target material has no explicit graphene-family component.	metal oxides, catalysts, polymers, electrolytes, substrates, controls, devices

sors or reagents) is graphene-based or not; to use only labels from the predefined taxonomy (Table S.3); and to return a valid JSON for each entry related to material production. We then performed a second, less granular categorization by grouping the materials identified in the first step into operational categories (Table S.4).

The manual validation was then performed with a gold set with 80 articles and 180 entries with details about the material production, stratified as 10 articles from each of the eight LDA topics. Each entry was reviewed against target-material name, production-process summary, evidence sentence, DOI/title metadata, and full-article context when needed. Manual labels were used as the gold standard for the validation tables and figures shown below.

Across the seven material-taxonomy fields used in the first step categorization (Table S.3), we calculated the precision, recall and F1-score using:

$$\text{Precision} = \frac{TP}{TP + FP}$$

$$\text{Recall} = \frac{TP}{TP + FN}$$

$$F_1 = 2 \cdot \frac{\text{Precision} \cdot \text{Recall}}{\text{Precision} + \text{Recall}}$$

where TP denotes true positives, corresponding to parameters correctly identified by both the manual annotation and the LLM; FP denotes false positives, corresponding to parameters incorrectly extracted by the LLM compared to the manual annotation; and FN denotes false negatives, corresponding to parameters present in the manual annotation but missed by the LLM. The label-level averaged precision was 0.962, recall was 0.964, and F1 was 0.963. The initial graphene-based/non-graphene-based target decision made by the LLM had TP = 154, TN = 26, FP = 0, and FN = 0. Finally, we calculated the precision, recall, and F1 score of material categorizations when the operational taxonomy represented in Table S.4 was used. This broader categorization was useful to establish correlations with

the LDA-generated topics and the parameter communities. The validation results for this step are shown in Table S.5.

Table S.5: Broad-category validation against the gold set. The material categories are mutually exclusive at the material description level and are evaluated one-versus-rest.

**Validation category**	**Support**	**Precision**	**Recall**	**F1**
GO non-composite	26	0.923	0.923	0.923
GO-based composite	56	0.932	0.982	0.957
rGO non-composite	6	1.000	0.833	0.909
rGO-based composite	29	0.966	0.966	0.966
Graphene non-composite (non-GO)	14	1.000	0.929	0.963
Graphene composite (non-GO)	18	1.000	0.944	0.971
Graphene-based composite, family unspecified	4	1.000	1.000	1.000
Graphene-based, family and composition unspecified	1	1.000	1.000	1.000
Non-graphene-based target	26	1.000	1.000	1.000
Weighted average	180	0.962	0.961	0.961

The residual disagreements were concentrated in the specificity of decisions rather than in the graphene-based/non-graphene-based boundary. The manual audit was especially useful in multi-material articles. For example, when it is reported the production of hematite nanoparticles, graphene nanosheets (GNS), and Al/hematite/GNS thermite,[S23] the gold labels exclude hematite from the graphene-family category while retaining the graphene nanosheet and graphene-based thermite entries. When it is reported the production of Pd/NRGO catalysts, in the gold labels they are classified as nitrogen-doped rGO-based catalyst materials rather than generic GO.[S24] Another example is the separation of a GO/LDH hybrid from a calcined rGO/Ni/MMO hybrid,[S25] rather than collapsing both into one undifferentiated "GO composite" class. These examples illustrate why entry-level final-target classification is necessary for this corpus.

C.6 Parameter Co-occurrence Community Construction

Parameter communities were constructed from a single global co-reporting network spanning the eight GO application domains. Candidate nodes were defined as the union of the top 125 parameters by independent-article frequency within each application topic, giving 558 unique parameter nodes after merging duplicate names across topics. Parameters were counted once per article, and topic labels were used only to balance the node universe and to interpret the final communities, not to assign community labels.

For each parameter pair i, j, a binary article–parameter matrix was used to compute the independent-article co-reporting count n_{ij}, the marginal article counts n_i and n_j, lift, normalized pointwise mutual information (NPMI), and a hypergeometric upper-tail enrichment p value. The association statistics were defined as

$$\text{lift}_{ij} = \frac{n_{ij}/N}{(n_i/N)(n_j/N)}, \tag{S.2}$$

and

$$\text{NPMI}_{ij} = \frac{\log\left(\frac{n_{ij}N}{n_i n_j}\right)}{-\log\left(\frac{n_{ij}}{N}\right)}, \tag{S.3}$$

where N is the number of independent articles in the analysis. Hypergeometric p values were corrected using the Benjamini–Hochberg procedure. Edges were retained in the enriched association graph only when they satisfied all four criteria: support $n_{ij} \geq 3$ articles, lift ≥ 1.5, NPMI ≥ 0.03, and FDR $q \leq 0.05$. Retained edges were assigned the weighted score

$$w_{ij} = \text{NPMI}_{ij} \log(1 + n_{ij}) \log_2(\text{lift}_{ij}) \sqrt{-\log_{10}(q_{ij})} \tag{S.4}$$

The parameter communities were discovered from a weighted co-reporting network in which nodes represent normalized parameter names and edges represent statistically en-

riched co-reporting within independent articles. The method identifies groups of parameters that tend to appear together in the same experimental records, forming communities that correspond to recurring reporting programs rather than isolated high-frequency terms.

The resulting significant graph contained 558 nodes and 15,699 enriched edges. To obtain a readable graph for community detection and visualization, the graph was sparsified by retaining the top 12 weighted associations for each node and then adding the strongest remaining edges where needed to preserve a minimum degree of two when possible. The sparsified graph contained 4,697 weighted edges and formed one connected component. Weighted Louvain community detection was run at resolution 1.6 across 25 random seeds; the reported partition used seed 9 and produced 26 communities with modularity 0.714. Seed-to-seed stability was high, with mean pairwise adjusted Rand index of 0.880 across the Louvain runs. Parameters outside the retained node universe were marked as `outside_graph` in downstream summaries.

Table S.6: Final parameter-association community-construction diagnostics.

Quantity	Value
Candidate node rule	Top 125 parameters per topic, unioned globally
Unique parameter nodes	558
Enriched-edge filters	$n_{ij} \geq 3$, lift ≥ 1.5, NPMI ≥ 0.03, FDR $q \leq 0.05$
Enriched edges before sparsification	15,699
Sparsification rule	Top 12 weighted associations per node plus minimum-degree support
Weighted edges after sparsification	4,697
Connected components	1
Louvain resolution	1.6
Louvain seeds tested	25
Reported seed	9
Detected communities	26
Modularity	0.714
Mean pairwise adjusted Rand index	0.880

Figure S.23 reports the most common parameters in representative communities, providing the parameter-level vocabulary behind the community labels used in the main text.

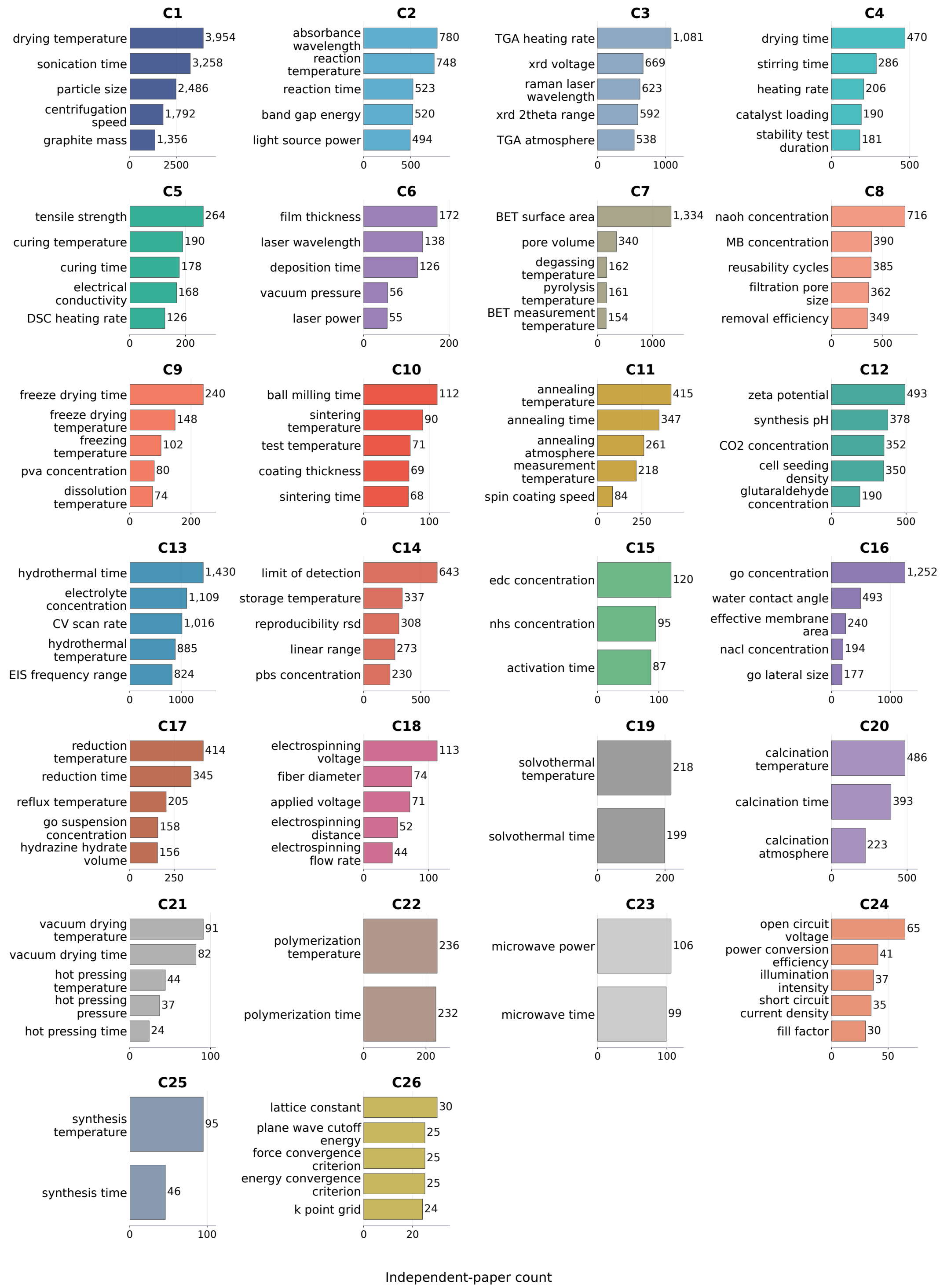

Figure S.23: Most common parameters in representative parameter communities from the final global parameter-association partition.

C.7 Communities stability across materials categories

The material stratification followed the same classification framework used for the material-counting analysis. Each individual material entry was assigned to only one material class. However, because a single paper can report multiple distinct materials, a paper could contribute to more than one material-specific group when it reported materials belonging to different classes. For example, a paper separately reporting GO, rGO, and a GO/polymer composite could contribute to each of the corresponding material groups. The resulting material-stratified parameter communities are shown in Figure S.24.

The communities provide a direct visual assessment of how parameter-community organization varies among material classes. Parameters related to the production of materials such as "drying temperature" and "sonication time" appear regardless of the stratified material. On the other hand, it is possible to observe material-specific parameters such as "reduction temperature" appearing in specific material classes (e.g., rGO composites and non-composite).

In addition, quantitative differences between the networks must be interpreted cautiously because the resulting communities depend not only on the underlying parameter co-occurrence relationships, but also on dataset size and hyperparameters of the algorithm used (see section C.6 for details on the hyperparameters and optimization procedure). This issue is particularly important because the complete-topic datasets and their corresponding material-specific subsets differ substantially in size. For example, the material groups associated with Catalysis contained only 7.4%–23.5% of the number of papers in the complete Catalysis dataset. Smaller groups consequently have less statistical power to retain parameter connections. We observe that, after stratification, the communities remain qualitatively similar, while showing differences in their relative frequencies and dominance across material classes.

For this reason, we complemented the reconstructed community analysis with a quantitative comparison of the underlying parameter co-occurrence patterns before applying any con-

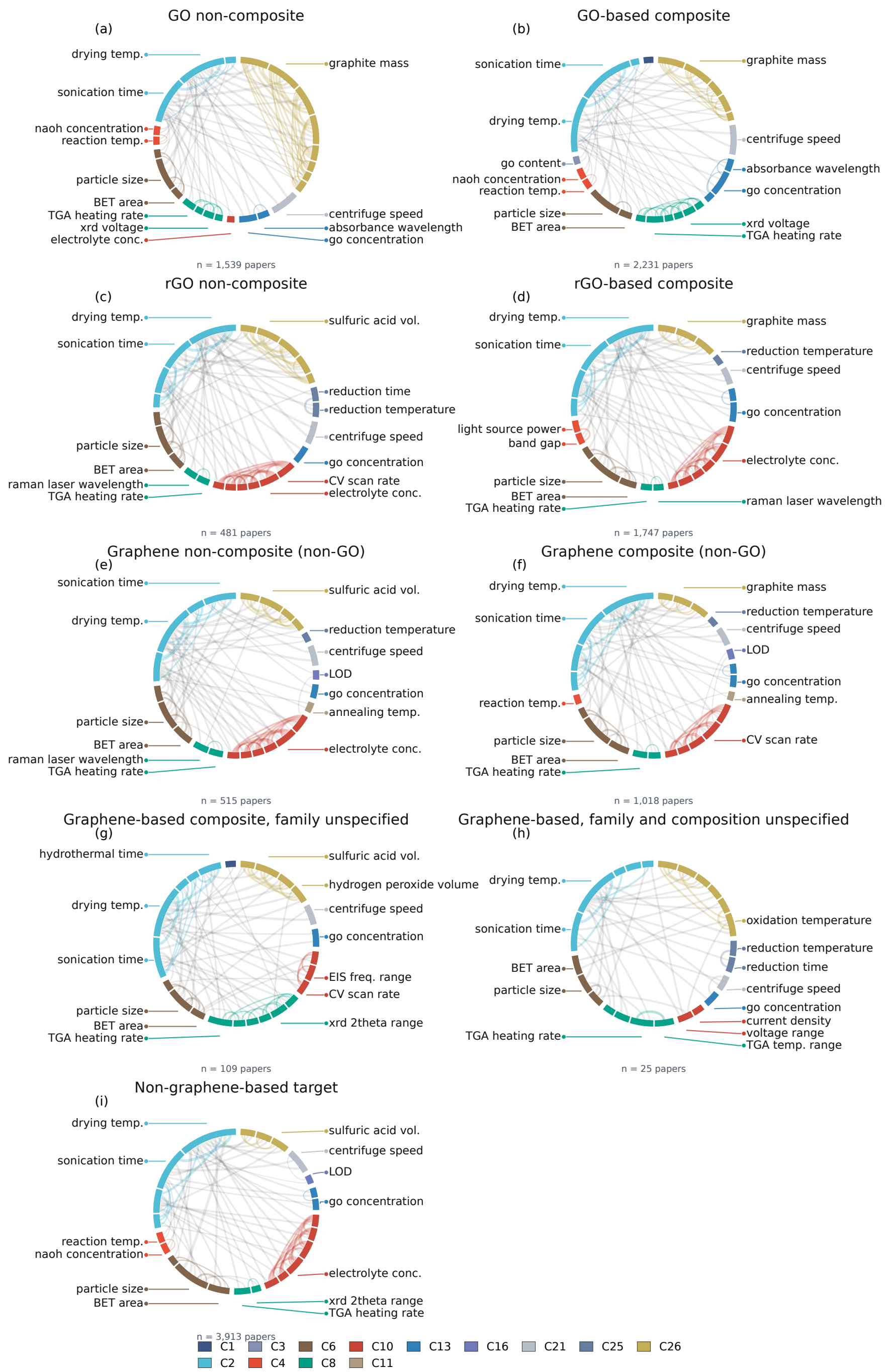

Figure S.24: Parameter communities after stratification by material class. The stratification follows the same material-class framework used for material counting. Individual material entries are assigned to a single class, while papers reporting multiple distinct material classes may contribute to more than one material-specific group.

nection threshold, statistical edge-selection procedure, edge pruning, or community-detection algorithm. This comparison evaluates whether the relative patterns of parameter pairs reported together differ across material classes without introducing additional dependence on the network-construction procedure. For each application topic and material class, we constructed a parameter co-occurrence matrix using a fixed universe of 558 parameters. For a certain group g relative to a combination of a certain topic i and a certain material class j, N_g denotes the number of papers in that group. For each pair of distinct parameters i and j, the co-occurrence value was defined as

$$C_{ij}^{(g)} = \frac{n_{ij}^{(g)}}{N_g},$$

where $n_{ij}^{(g)}$ is the number of papers in group g in which both parameters i and j were reported. Thus, $C_{ij}^{(g)}$ represents the fraction of papers in a group reporting a given parameter pair. Using fractions rather than raw counts allows co-occurrence patterns from groups of different sizes to be compared on the same scale. Each group was represented by a (558×558) symmetric co-occurrence matrix. The diagonal elements, corresponding to self-pairs, were excluded from the analysis. Retaining the upper triangular portion of each matrix resulted in

$$\frac{558 \times 557}{2} = 155{,}403$$

possible pairs of distinct parameters. The upper triangle was then flattened into a co-occurrence vector,

$$\mathbf{c}^{(g)} = \left(C_{12}^{(g)}, C_{13}^{(g)}, \ldots, C_{557,558}^{(g)}\right).$$

The same parameter ordering and, consequently, the same ordered list of 155,403 parameter pairs were used for every application topic, material class, and matched random group. Therefore, each position in the resulting vectors represented the same parameter

pair in every comparison. Parameter pairs that were not co-reported in a given group were assigned a co-occurrence value of zero and retained in the vector. For each material class, its co-occurrence vector was compared with the vector calculated from all papers in the corresponding application topic. Similarity was quantified using cosine similarity:

$$\text{Cosine similarity} = \frac{\mathbf{c}^{(\text{material})} \cdot \mathbf{c}^{(\text{topic})}}{\|\mathbf{c}^{(\text{material})}\| \, \|\mathbf{c}^{(\text{topic})}\|}.$$

A cosine similarity of 1 indicates identical relative co-occurrence patterns across all parameter pairs in the material-specific group and the complete application topic. Lower values indicate increasing differences in the relative prominence of parameter-pair associations. No minimum-count threshold, statistical edge-selection procedure, edge pruning, or community-detection algorithm was applied before these comparisons. The analysis therefore evaluates the complete underlying co-occurrence pattern rather than only the subset of connections retained after network construction. We calculated these co-occurrence similarities for all eight application topics and nine material classes. Figure S.25 presents the resulting cosine similarities together with the number of papers that contribute to each combination of topic and material. The number of papers is shown because similarity estimates from smaller subsets are expected to be more variable and should therefore be interpreted together with sample size.

C.8 Manual validation

A ground-truth (GT) dataset was manually constructed from 40 graphene oxide (GO) articles, comprising five articles from each of eight application-based topics. For each article, all relevant numerical parameter records were manually annotated. Each annotation included the parameter name, parameter value, and supporting evidence text. Because the GT dataset was created by annotating relevant textual occurrences, it could contain more than one annotation for the same underlying experimental record when that information was

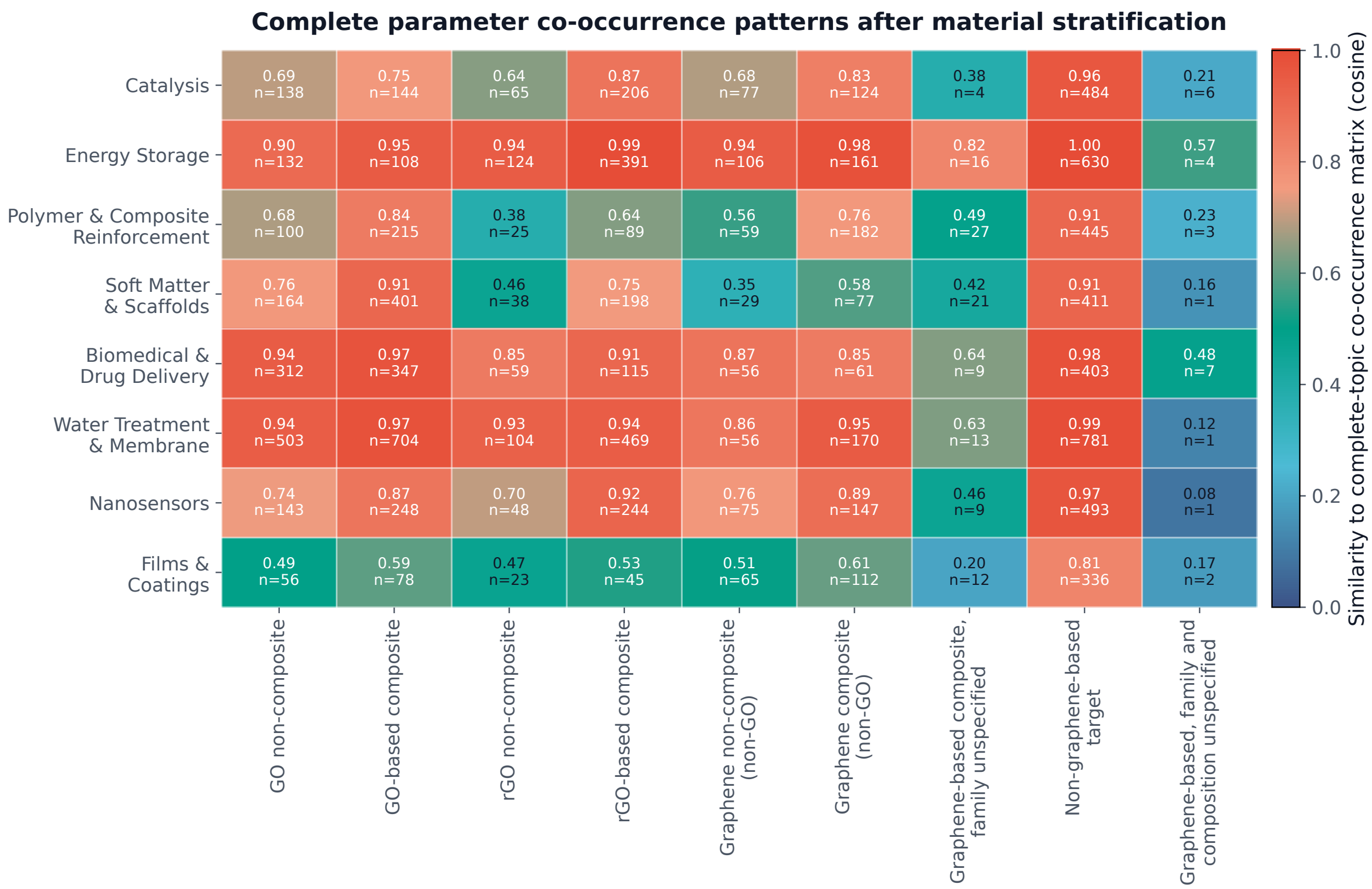

Figure S.25: Similarity of parameter co-occurrence patterns for topics after material stratification. For each application topic and material group, a complete parameter co-occurrence matrix was constructed using the fraction of papers reporting each parameter pair together. Cosine similarity compares each material-specific matrix with the corresponding complete-topic matrix; values closer to 1 indicate more similar overall co-occurrence patterns. Each cell reports the cosine similarity and the number of contributing papers (n). All 155,403 possible pairs among the 558 parameters were included without edge filtering, pruning, or community detection. Material classes are mutually exclusive for individual material entries, but paper-level groups may overlap when a paper reports materials from multiple classes. The “non-graphenic target entry present” group therefore does not represent exclusively non-graphene papers.

reported repeatedly within an article (e.g., in the Experimental section and subsequently in the Results and Discussion). The complete occurrence-level GT dataset is provided on Github.

As defined in the LLM prompt (section C.1), the model was instructed to extract one non-redundant record for each parameter–value pair from an article. Consequently, before calculating the performance metrics, the GT dataset was converted to a reference dataset. Repeated annotations were consolidated when they referred to the same parameter name, parameter value, article, and underlying experimental record. This procedure ensured that the reference dataset used for validation represented the same unit of extraction as the LLM output: a unique scientific record rather than every textual mention of that record. The validation dataset was then generated by comparing the deduplicated GT records with the records extracted by the LLM.

Matching was performed at the record level on the basis of the parameter name and associated value. Evidence text was retained as provenance information to allow traceability to the original article, but was not used as a matching criterion. This choice accounts for legitimate differences in evidence-span selection: manual annotations generally retained the complete sentence, whereas the LLM could retain a shorter span that was sufficient to support the same parameter–value record. The resulting validation dataset identifies true positives, false positives, and false negatives and was used to calculate precision, recall, and F1 score. This evaluation is aligned with the non-redundant dataset used in the downstream co-occurrence and CO_2-uptake prediction analyses. The final validation dataset (VD) for which the performance metrics were calculated is on GitHub (with a README file).

D Predictive Model for CO_2 Adsorption Capacity

D.1 Dataset Construction

A dataset of CO_2 equilibrium adsorption capacity was constructed to train a supervised regression model. Two literature-derived structures were used at different stages of the workflow. LDA topics and BERTopic subtopics were used first to scout articles likely to contain CO_2 uptake data. This search screened subtopic labels and representative terms for CO_2-, carbon-dioxide-, adsorption-, capture-, uptake-, separation-, porous-carbon-, MOF-, and sorbent-related language, followed by source-level validation of candidate XML/PDF text and tables; the retained subtopics are listed in Table S.7. Some retained articles were assigned to non-Water-Treatment LDA topics because LDA was performed at the full-document level and therefore reflected the broader material or application context. For example, porous-carbon CO_2 capture articles can appear within the Energy Storage macro-topic because they share synthesis, activation, nitrogen-doping, surface-area, and electrochemical-carbon vocabulary with supercapacitor and electrode studies.

Table S.7: Subtopics represented in the CO_2 adsorption modeling dataset. The document count corresponds to the size of the subtopic before row-level curation.

LDA topic	Subtopic index	Subtopic label	Documents
Catalysis	18	CO_2 capture on amine-functionalized MOF and porous-carbon adsorbents	129
Catalysis	1	Non-precious ORR electrocatalysts	523
Catalysis	104	Hydrogen spillover mechanisms in carbon- and MOF-based storage materials	25
Energy Storage	56	Porous carbon adsorbents for CO_2 capture	152
Biomedical & Drug Delivery	1	Graphene-based adsorbents for dye removal	233
Water Treatment & Membrane	71	Biomass-based CO_2 capture and adsorption	41
Water Treatment & Membrane	8	CO_2/CH_4 separation with MOF- and graphene-based mixed-matrix membranes	225
Water Treatment & Membrane	55	Photocatalytic CO_2-to-CH_4 reduction over engineered brookite-TiO_2	56

The CO_2 adsorption data were extracted with a Python workflow that combined LLM-assisted parameter extraction, topic-guided document discovery, source-table parsing, and figure digitization. XML/PDF parameter extraction used `moonshotai/kimi-k2-thinking` through an OpenRouter/OpenAI-compatible API. Source XML tables were parsed with Python regular expressions, pandas, NumPy, and `xml.etree.ElementTree`; when uptake values were only available as plotted isotherms, candidate PDF pages were rendered as PNG images using `pdftoppm`, calibrated with manually recorded plot bounds and axis ranges, and digitized with OpenCV color masks by sampling trace positions at predefined pressures and converting pixel coordinates to pressure and capacity by linear interpolation. Rows were retained only when source-level checks confirmed material identity, CO_2 adsorption capacity with recoverable units, adsorption pressure, adsorption temperature, and low-pressure measurement conditions; capacities were converted to mg/g and pressures to bar before downstream preprocessing and regression modeling.

The dataset was reduced to include only instances in which the adsorbent belonged to a family containing GO or graphene, according to the `material_taxonomy` classification (see Table S.4). The possible values are GO non-composite, GO-based composite, rGO non-composite, rGO-based composite, graphene composite, and graphene non-composite, as shown in Figure S.26. The complete dataset comprises 37 distinct DOIs, corresponding to 698 unique instances distributed across six families of graphene-based materials. A CSV file with the dataset is provided on Github.

D.2 Model Construction

The prediction problem can be physically interpreted as follows: the features `pressure` and `temperature` address external thermodynamic conditions that are controlled during the experiment; the features `BET surface area` and `pore volume` describe morphological properties of the tested adsorbent material; and the categorical feature `material_taxonomy`

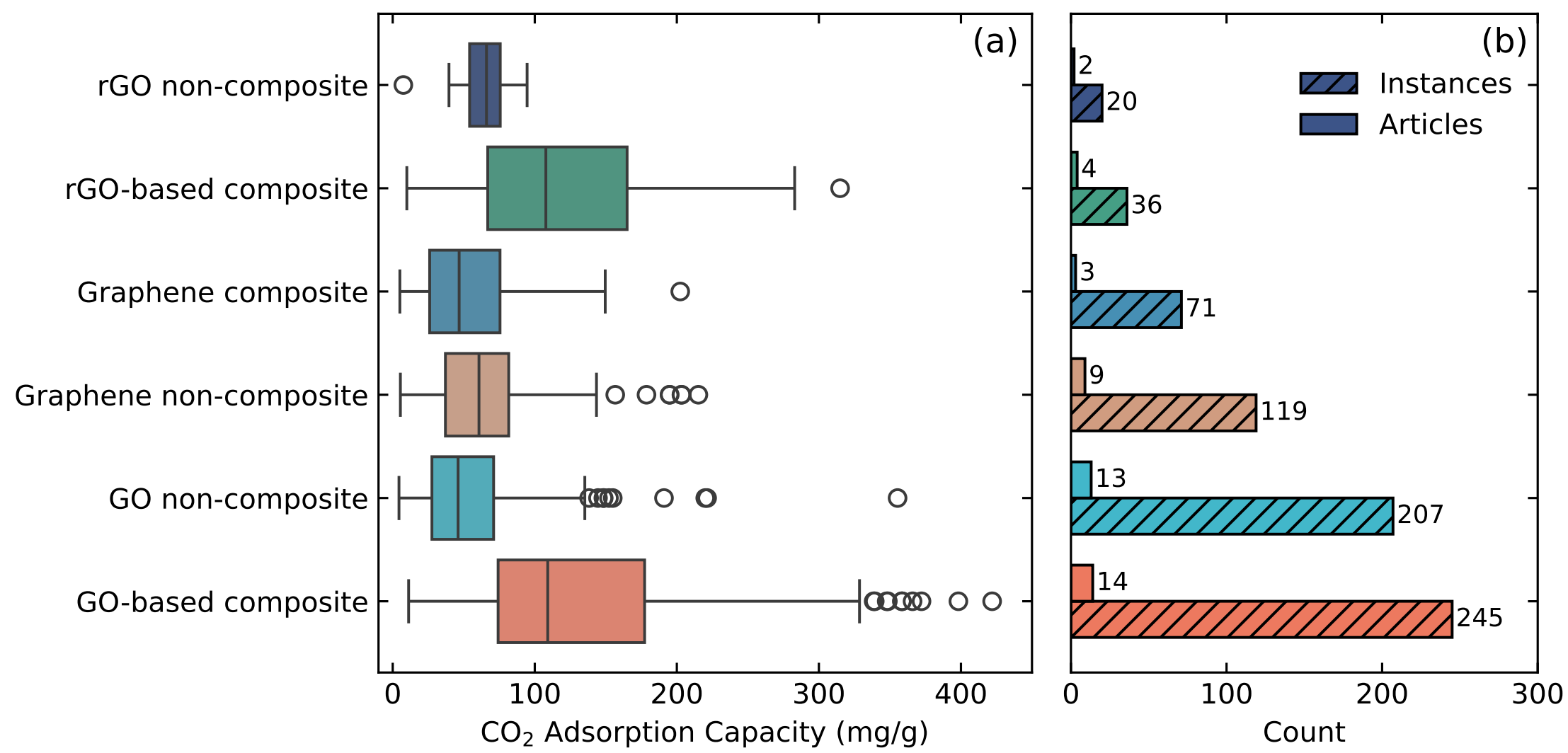

Figure S.26: CO_2 adsorption capacity dataset. (a) Distribution of adsorption capacity by material family. (b) Distribution of articles and instances.

encodes the chemical properties of the tested adsorbent material. This categorical variable was incorporated into the model using one-hot encoding.

The final model used 5 descriptors as shown in Table S.8:

Table S.8: Feature definitions, units, and value ranges.

Feature	Unit	Min	Median	Max	Non-null Count
Pressure	bar	0.05	0.70	1.00	698
Temperature	K	273	298	423	698
BET surface area	$m^2\,g^{-1}$	10	205	2640	654
Pore volume	$cm^3\,g^{-1}$	0.03	0.46	3.08	458
Material subfamily	categorical		6 classes		698

The regression model was an Extra-Trees regressor[S26] from the Scikit-Learn Python library, using 500 trees (`n_estimators`) and all other hyperparameters set to their default values, with a fixed random seed of 42. Random Forest (from `sklearn`) and Gradient Boosting (from `xgboost`) were also tested, with similar results.

D.3 Model Evaluation

The machine learning model was evaluated using three train-test split strategies: 5-fold random-split cross-validation, leave-one-DOI-out cross-validation, and temporal holdout (training $\leq$ 2018; test $>$ 2018). In all cases, the performance metrics were calculated as follows. R^2 measures the proportion of the variance in the observed data that is explained by the predictive model:

$$R^2 = 1 - \frac{\sum_{i=1}^{n}(y_i - \hat{y}_i)^2}{\sum_{i=1}^{n}(y_i - \bar{y})^2} \tag{S.5}$$

RMSE (Root Mean Squared Error) quantifies the square-root average magnitude of prediction errors, giving higher weight to large deviations:

$$\text{RMSE} = \sqrt{\frac{1}{n}\sum_{i=1}^{n}(y_i - \hat{y}_i)^2} \tag{S.6}$$

MAE (Mean Absolute Error) represents the average absolute difference between predicted and observed values:

$$\text{MAE} = \frac{1}{n}\sum_{i=1}^{n}|y_i - \hat{y}_i| \tag{S.7}$$

MAD (Mean Absolute Deviation) represents the average absolute deviation of the data values from their mean:

$$\text{MAD} = \frac{1}{n}\sum_{i=1}^{n}|y_i - \bar{y}_i| \tag{S.8}$$

STD (Standard Deviation) represents the standard deviation of the data values around

their mean:

$$\text{STD} = \sqrt{\frac{1}{n}\sum_{i=1}^{n}(y_i - \bar{y}_i)^2} \tag{S.9}$$

In the equations above, y_i denotes the observed (true) value for sample i, $\hat{y}_i$ denotes the corresponding model-predicted value, $\bar{y}$ represents the mean of the observed values, and n is the total number of samples.

Table S.9 presents the individual results obtained using the leave-one-DOI-out cross-validation methodology. In this procedure, the machine learning model is trained on all instances from 36 articles and tested on all instances from a single held-out article. This procedure is repeated such that each article is used exactly once as the test set.

Table S.9: Performance metrics for the ExtraTrees-based predictive model across individual DOI-based test sets. n_{test} denotes the number of test samples, while R^2, MAE, and RMSE represent the coefficient of determination, mean absolute error, and root mean squared error, respectively.

DOI	n_{test}	Year	R^2	MAE (mg/g)	RMSE (mg/g)
10.1039/c3ee23421e	51	2013	0.850	24.77	36.97
10.1016/j.jcou.2021.101666	20	2021	0.807	19.21	23.75
10.1016/j.psep.2021.02.014	7	2021	0.775	13.18	17.96
10.1016/j.colsurfa.2021.126640	8	2021	0.750	20.03	25.32
10.1039/c3ra42022a	107	2013	0.614	19.90	25.39
10.1016/j.jcou.2022.102371	9	2022	0.541	21.17	27.76
10.1016/j.micromeso.2019.109761	22	2019	0.527	45.77	53.19
10.1016/j.jcou.2015.12.001	5	2015	0.414	21.06	24.78
10.1016/j.ijhydene.2016.05.162	7	2016	0.278	17.15	20.73
10.1016/j.micromeso.2014.08.012	6	2014	0.269	37.84	40.44
10.1016/j.fuel.2019.03.011	8	2019	0.099	42.03	63.84
10.1016/j.ces.2023.119572	7	2023	0.098	10.73	12.83
10.1039/c6ra13590k	12	2016	0.077	32.75	45.46
10.1039/c4ra09314c	114	2014	-0.062	19.19	25.94
10.1016/j.jiec.2016.01.032	6	2016	-0.074	4.15	4.74
10.1016/j.cej.2014.11.037	112	2014	-0.140	18.63	22.57
10.1016/j.jiec.2019.02.016	2	2019	-0.239	93.28	103.82
10.1016/j.jece.2021.105839	10	2021	-0.323	57.08	74.58
10.1016/j.ijhydene.2017.12.070	3	2018	-0.392	152.71	166.71
10.1016/j.jpowsour.2016.08.042	6	2016	-0.542	65.07	81.28
10.1016/j.carbon.2014.09.042	14	2014	-0.545	50.34	59.40
10.1016/j.jallcom.2020.157534	13	2020	-0.743	26.29	31.06
10.1016/j.jiec.2014.12.021	31	2014	-2.185	34.14	41.35
10.1021/acs.est.7b03308	7	2017	-2.606	152.26	163.07
10.1016/j.ijhydene.2014.05.059	8	2014	-2.827	49.01	52.16
10.1021/am402661t	53	2013	-6.571	52.86	62.89
10.1016/j.scitotenv.2021.151522	2	2021	-8.216	97.58	103.52
10.1016/j.cej.2020.124022	3	2020	-9.917	17.17	18.02
10.1016/j.matchemphys.2021.124788	11	2021	-10.640	126.04	138.31
10.1016/j.micromeso.2020.110786	18	2020	-12.630	51.59	54.37
10.1016/j.jcou.2017.05.018	3	2017	-12.632	19.01	23.58
10.1016/j.jssc.2018.07.022	3	2018	-28.674	45.06	49.63
10.1016/j.memsci.2016.04.029	5	2016	-34.380	23.87	27.74
10.1016/j.psep.2023.12.041	2	2023	-64.233	116.58	122.60
10.1016/j.ijbiomac.2018.12.071	1	2018	–	15.71	15.71
10.1016/j.apsusc.2018.08.062	1	2018	–	22.18	22.18
10.1016/j.seppur.2023.123448	1	2023	–	63.08	63.08

References

(S1) Blei, D. M.; Ng, A. Y.; Jordan, M. I. Latent Dirichlet Allocation. *Journal of Machine Learning Research* **2003**, *3*, 993–1022.

(S2) Grootendorst, M. BERTopic: Neural Topic Modeling with a Class-based TF-IDF Procedure. *arXiv preprint arXiv:2203.05794* **2022**,

(S3) Crossref REST API. `https://api.crossref.org/`, Accessed: 2025-09-09.

(S4) Web of Science Core Collection. `https://www.webofscience.com/`, Accessed: 2025-09-09.

(S5) Selenium WebDriver. `https://www.selenium.dev/documentation/webdriver/`, Accessed: 2025-09-09.

(S6) Elsevier Developer Portal. `https://dev.elsevier.com/`, Accessed: 2025-09-09.

(S7) Springer Nature API Portal. `https://api.springernature.com/`, Accessed: 2025-09-09.

(S8) Wei, H.; Kong, L.; Chen, J.; Zhao, L.; Ge, Z.; Yang, J.; Sun, J.; Han, C.; Zhang, X. Vary: Scaling up the Vision Vocabulary for Large Vision-Language Models. *arXiv preprint arXiv:2312.06109* **2023**,

(S9) Wei, H.; Liu, C.; Chen, J.; Wang, J.; Kong, L.; Xu, Y.; Ge, Z.; Zhao, L.; Sun, J.; Peng, Y.; others General OCR Theory: Towards OCR-2.0 via a Unified End-to-end Model. *arXiv preprint arXiv:2409.01704* **2024**,

(S10) Smith, R. An Overview of the Tesseract OCR Engine. 2007 Ninth International Conference on Document Analysis and Recognition (ICDAR). 2007; pp 629–633.

(S11) Bird, S.; Klein, E.; Loper, E. *Natural Language Processing with Python*; O'Reilly Media, 2009.

(S12) Miller, G. A. WordNet: A Lexical Database for English. *Communications of the ACM* **1995**, *38*, 39–41.

(S13) Van Rossum, G.; Drake Jr., F. L. Python 3 Reference Manual. Python Software Foundation, 2009; `https://docs.python.org/3/`.

(S14) Salton, G.; Buckley, C. Term-Weighting Approaches in Automatic Text Retrieval. *Information Processing & Management* **1988**, *24*, 513–523.

(S15) ehek, R.; Sojka, P. Software Framework for Topic Modelling with Large Corpora. Proceedings of the LREC 2010 Workshop on New Challenges for NLP Frameworks. Valletta, Malta, 2010; pp 45–50.

(S16) R"oder, M.; Both, A.; Hinneburg, A. Exploring the Space of Topic Coherence Measures. Proceedings of the Eighth ACM International Conference on Web Search and Data Mining (WSDM). 2015; pp 399–408.

(S17) Zhang, Y.; Li, M.; Long, D.; Zhang, X.; Lin, H.; Yang, B.; Xie, P.; Yang, A.; Liu, D.; Lin, J.; Huang, F.; Zhou, J. Qwen3 Embedding: Advancing Text Embedding and Reranking Through Foundation Models. *arXiv preprint arXiv:2506.05176* **2025**,

(S18) McInnes, L.; Healy, J.; Saul, N.; Groberger, L. UMAP: Uniform Manifold Approximation and Projection. *Journal of Open Source Software* **2018**, *3*, 861.

(S19) Campello, R. J. G. B.; Moulavi, D.; Sander, J. Density-Based Clustering Based on Hierarchical Density Estimates. Advances in Knowledge Discovery and Data Mining (PAKDD). 2013; pp 160–172.

(S20) OpenRouter OpenRouter API. `https://openrouter.ai/`, 2025; `https://openrouter.ai/`, Accessed: 2025-02-19.

(S21) et all, K. T. Kimi K2: Open Agentic Intelligence. 2026; `https://arxiv.org/abs/2507.20534`.

(S22) Tukey, J. W. *Exploratory Data Analysis*; Addison-Wesley Publishing Company: Reading, MA, 1977.

(S23) Fertassi, M. A.; Belkhiri, S.; Touidjine, S.; Hamdane, A.; Kechit, H.; Boulkadid, M. K.; Khimeche, K. Enhancing the thermal decomposition of composite modified double base propellant using Al/hematite and Al/hematite/graphene thermites. *Reaction Kinetics, Mechanisms and Catalysis* **2023**, *136*, 31273149.

(S24) Abasabadi, R. K.; Khodadadi, A. A.; Mortazavi, Y. Effects of nitrogen-containing functional groups of reduced graphene oxide as a support for Pd in selective hydrogenation of cinnamaldehyde. *Research on Chemical Intermediates* **2021**, *47*, 14291446.

(S25) Yang, Z.; Ji, S.; Gao, W.; Zhang, C.; Ren, L.; Tjiu, W. W.; Zhang, Z.; Pan, J.; Liu, T. Magnetic nanomaterial derived from graphene oxide/layered double hydroxide hybrid for efficient removal of methyl orange from aqueous solution. *Journal of Colloid and Interface Science* **2013**, *408*, 2532.

(S26) Geurts, P.; Ernst, D.; Wehenkel, L. Extremely randomized trees. *Machine Learning* **2006**, *63*, 342.
